# The BEEHAVE_ecotox_ Model—Integrating a Mechanistic Effect Module into the Honeybee Colony Model

**DOI:** 10.1002/etc.5467

**Published:** 2022-10-04

**Authors:** Thomas G. Preuss, Annika Agatz, Benoit Goussen, Vanessa Roeben, Jack Rumkee, Liubov Zakharova, Pernille Thorbek

**Affiliations:** ^1^ Bayer Monheim am Rhein Germany; ^2^ Institute for Biological Analytics & Consulting Roßdorf Germany; ^3^ Syngenta, Bracknell Berkshire United Kingdom; ^4^ BASF SE Limburgerhof Germany

**Keywords:** Ecotoxicology, honeybee, pesticides, population modeling, risk assessment

## Abstract

Mechanistic effect models are powerful tools for extrapolating from laboratory studies to field conditions. For bees, several good models are available that can simulate colony dynamics. Controlled and reliable experimental systems are also available to estimate the inherent toxicity of pesticides to individuals. However, there is currently no systematic and mechanistic way of linking the output of experimental ecotoxicological testing to bee models for bee risk assessment. We introduce an ecotoxicological module that mechanistically links exposure with the hazard profile of a pesticide for individual honeybees so that colony effects emerge. This mechanistic link allows the translation of results from standard laboratory studies to relevant parameters and processes for simulating bee colony dynamics. The module was integrated into the state‐of‐the‐art honeybee model BEEHAVE. For the integration, BEEHAVE was adapted to mechanistically link the exposure and effects on different cohorts to colony dynamics. The BEEHAVE_ecotox_ model was tested against semifield (tunnel) studies, which were deemed the best study type to test whether BEEHAVE_ecotox_ predicted realistic effect sizes under controlled conditions. Two pesticides used as toxic standards were chosen for this validation to represent two different modes of action: acute mortality of foragers and chronic brood effects. The ecotoxicological module was able to predict effect sizes in the tunnel studies based on information from standard laboratory tests. In conclusion, the BEEHAVE_ecotox_ model is an excellent tool to be used for honeybee risk assessment, interpretation of field and semifield studies, and exploring the efficiency of different mitigation measures. The principles for exposure and effect modules are portable and could be used for any well‐constructed honeybee model. *Environ Toxicol Chem* 2022;41:2870–2882. © 2022 Bayer AG & Sygenta, et al. *Environmental Toxicology and Chemistry* published by Wiley Periodicals LLC on behalf of SETAC.

## INTRODUCTION

Honeybees are considered important pollinators, providing a vital ecosystem service (European Food Safety Authority [EFSA], 2013). Bees are potentially exposed to plant protection products when foraging in agricultural landscapes. Therefore, the risk to bees is assessed within the environmental risk assessment of pesticides. However, social insects such as honeybees have complex interactions both within their colonies and with their environment. This complexity can pose a challenge when predicting impacts from pesticide exposure on colony dynamics based only on information about inherent toxicity to, and exposure of, individuals from laboratory tests. Modeling offers a powerful tool to capture these complex interactions. For the environmental risk assessment of pesticides, modeling approaches are increasingly applied, for example, to reduce uncertainty (EFSA Panel on Plant Protection Products and their Residues, 2014).

An extensive review of existing colony models was conducted by Becher et al. (2013). Since then, several models have been published (EFSA et al., 2021), such as the Baveco model, the US Environmental Protection Agency (USEPA) model, the Croft model, or the BEEHAVE model (Baveco et al., 2016; Becher et al., 2014; Croft et al., 2018; Kuan et al., 2018). These models have different levels of realism and varying degrees of simplification of the complex dynamics within a colony and the interactions with its environment. It is important that the level to which a model should be simplified depend on its purpose (Grimm & Railsback, 2005; Railsback & Grimm, 2012). In addition, several approaches exist to describe the relationship between exposure and toxicity, such as dose–response models, toxicokinetic/toxicodynamic models, and methods for predicting exposure (EFSA Panel on Plant Protection Products and their Residues et al., 2018; Organisation for Economic Co‐operation and Development [OECD], 2006).

Model purposes generally fall into three categories, demonstration (designed to explore ideas and theories), understanding (aimed at exploring how different components of a system interact), and prediction (focus on numerical accuracy). When model applications lead to unhelpful advice, it is often because a model has been used for a purpose for which it was not designed (Grimm, Johnston, et al., 2020). For a realistic honeybee risk assessment, a model needs to be aimed at prediction, that is, it should have emergent properties regarding exposure and effects on different cohorts within the colony (EFSA, 2015; USEPA, 2012). The BEEHAVE model is aimed at prediction and includes many interactions within the colony and between the colony and the landscape (Becher et al., 2014).

When considering exposure of bees to pesticides, it is important to be aware that field exposure is heterogenous. Not all cohorts and individuals of these cohorts experience the same exposure levels. Some toxic effects are cohort specific (EFSA, 2013). Plant protection products are applied in a nonsynchronous pattern across the landscape and vary in their concentrations on the plant, and in water, pollen, nectar, and any dust generated from drilling seed (Chauzat et al., 2006; Gierer et al., 2019; Krahner et al., 2021; Kyriakopoulou et al., 2017; Schmolke et al., 2018). Therefore, an ecotoxicological model for bees needs to be able to simulate various multiple exposure routes and account for contact and oral exposure (EFSA, 2015). Moreover, exposure varies because different cohorts of bees feed on different food sources. Brood and nurse bees get their protein mainly via pollen (young larvae are fed on brood food secreted by the hypopharyngeal gland of nurse bees), whereas foragers almost exclusively consume honey and nectar (Haydak, 1970; Seeley, 1995; Winston, 1991). The exposure pathway through contaminated water, for example, puddles or guttation droplets, has also been raised as a concern in recent discussions (EFSA, 2013). Bees have been found to forage for water on hot days to cool the colony (Johansson & Johansson, 1978; Lindauer, 1954, 1955; Nicolson, 2009; Southwick & Heldmaier, 1987). In addition, water is collected after cold and rainy days to dilute honey (Nicolson, 2009).

In 2015, the EFSA evaluated the BEEHAVE model for its use in honeybee risk assessment (EFSA, 2015). Although there were some concerns regarding the use of the current version for the risk assessment of pesticides, the EFSA has used the model to quantify the specific protection goals for honeybee risk assessment (EFSA et al., 2021). The main criticisms of the BEEHAVE model were insufficient validation and lack of an ecotoxicological module. In the meantime, validation studies have been published covering a broad range of studies in different regions (Agatz et al., 2019; Schmolke et al., 2020). So far, the BEEHAVE model has been successfully used by several research groups to improve the understanding of how pesticide effects impact colony resilience without an explicit exposure–effect module (Becher et al., 2014; Henry et al., 2017; Rumkee et al., 2015; Thorbek, Campbell, Sweeney, & Thompson, 2017; Thorbek, Campbell, & Thompson, 2017). However, different approaches and algorithms were used to simulate various types of toxic effects on different cohorts. These validation studies and sensitivity analyses highlighted the type of effects that have the largest impact, when in the year the colony is most vulnerable, and which cohorts are the most vulnerable for impacts at the colony level (Henry et al., 2017; Rumkee et al., 2015; Thorbek, Campbell, Sweeney, & Thompson, 2017; Thorbek, Campbell, & Thompson, 2017). However, an ecotoxicological module that can link exposure from the agricultural landscapes with information on ecotoxicological effects is still lacking (EFSA, 2015). Ideally, this module could be parametrized with standard regulatory bee and residue studies (EFSA, 2015). This way, exposure and effects can be quantitatively linked by using information from established standard study designs, such as the bee acute toxicity tests for oral and contact exposure (OECD, 1998a, 1998b), the bee larval toxicity test for repeated exposure (OECD, 2016), and pesticide residue studies (Croft et al., 2018; OECD, 2009).

The BEEHAVE model is an ideal candidate for the development of such an ecotoxicological module. This model is currently the only freely available honeybee model that already interlinks colony dynamics with the surrounding landscape (EFSA et al., 2021). Furthermore, it was developed in a modular fashion; the three linked modules are the colony model, the foraging model, and a varroa model (Becher et al., 2014). A modular approach allows one to exchange different aspects as new knowledge becomes available, enabling the model to be kept up to date and facilitating collaboration of experts from different fields (Forbes et al., 2020; Roeben et al., 2020).

We present an ecotoxicological module (BEEHAVE_ecotox_) that can realistically predict the risk of pesticides for a specific use pattern. This is achieved by mechanistically linking exposure in different compartments of the landscape with forager behavior. The module also considers the subsequent route of foraging products through the colony, which determines how other cohorts are exposed and hence potentially affected by the pesticide. It also considers the storage of honey in multiple compartments. The module differentiates between capped and uncapped honey stores, which are used to ripen and store the honey in the hive (Eyer et al., 2016; Seeley, 1995; Winston, 1991).

The module was designed to be parameterized with standard laboratory and higher tier honeybee studies as well as residue studies and is able to apply dose–response relationships with a mechanistic link between exposure and effects. The aim of the new module development was to keep the bee biology as implemented in the original BEEHAVE model (Becher et al., 2014) and only adapt it where necessary to enable a better representation of exposure and effects.

Because the BEEHAVE model has already been extensively validated (Agatz et al., 2019; Becher et al., 2014; Schmolke et al., 2020), the focus of the present study was to implement, analyze, and validate the additional functionality of representing exposure to and effects from plant protection products.

## MATERIALS AND METHODS

### The model

The BEEHAVE_ecotox_ model was implemented in the freely available agent‐based modeling environment NetLogo, Ver 5.3.1 (Wilensky, 1999). It is based on the BEEHAVE_BeeMapp2016 version of BEEHAVE (2016).

The original BEEHAVE model (Becher et al., 2014) was developed to study the impact of multiple stressors on honeybee colonies and is built on an extensive review of available bee models and bee literature (Becher et al., 2013). The structure of BEEHAVE consists of three modules, a colony module, a foraging module, and a varroa module, which allows to explore how different factors inside and outside the hive interact to shape the colony dynamics. A full description of the original BEEHAVE model can be found in Becher et al. (2014). Since 2014, other submodels have been developed, for instance, BEESCOUT accounts for diverse changes in landscapes (Becher et al., 2016) and BEEHAVE‐Weather (BEEHAVE, 2016) allows for easy creation of weather input for model simulations.

**Figure 1:**
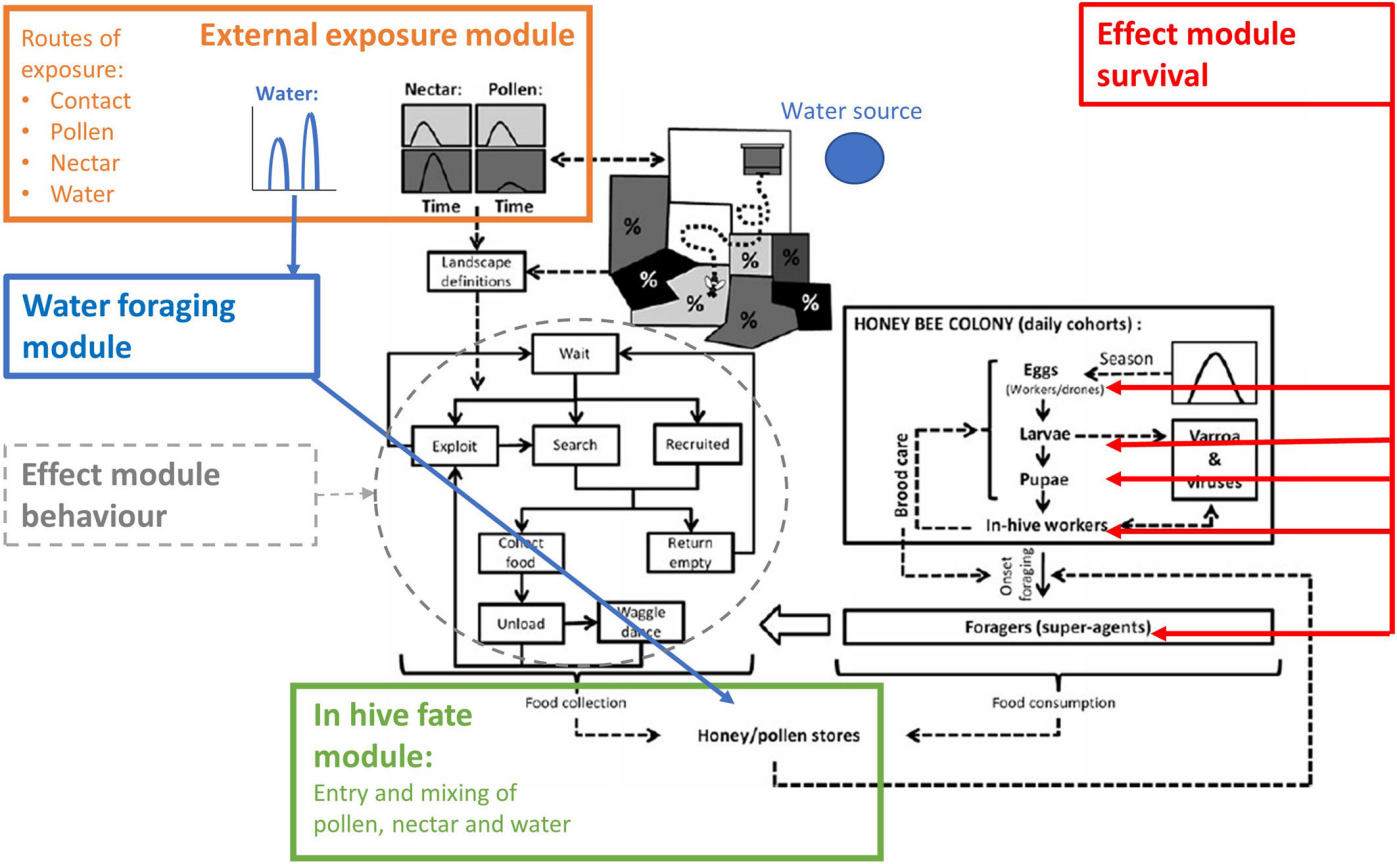
Flow‐chart for the main additions made to the original BEEHAVE model (Becher et al., 2014). Black: original model. Orange: landscape exposure module includes contact exposure and exposure via collected pollen, nectar, and water. Blue: water foraging module. Green: in‐hive exposure module accounts for incoming forage and mixing within the colony. Red: effect module for survival includes dose–response relationships for contact and oral exposure of different cohorts. Gray: effect module for behavior, which is not implemented in the present model version.

The purpose of BEEHAVE_ecotox_ is to mechanistically integrate exposure and ecotoxicological procedures into the BEEHAVE model, which can be parameterized with standard ecotoxicological regulatory studies and exposure (residue) studies, such as the bee acute toxicity tests for oral and contact exposure (OECD, 1998b, 1998a), the bee larval toxicity test for repeated exposure (OECD, 2016), and pesticide residue studies (Croft et al., 2018; OECD, 2009). The BEEHAVE_ecotox_ model introduces minor additions to BEEHAVE by incorporating an external exposure module, an in‐hive fate module, a water‐foraging module, and an effect module (Figure 1). For practicality, these changes are incorporated in different procedures throughout the colony and foraging modules of the original BEEHAVE. In the present study, we only give an overview of the overall structure and processes of the model. In addition, the verification, sensitivity analysis, and validation are described. A detailed model description of BEEHAVE_ecotox_ is provided in the Supporting Information, Appendix A1, following the Overview, Design concepts, Details (ODD) protocol for describing individual‐based models (Grimm et al., 2006, 2010; Grimm, Johnston, et al., 2020; Grimm, Railsback, et al., 2020). The model code is provided in the Supporting Information, Appendix A7. The additions in the code have been separated as far as possible from the original BEEHAVE code and are marked with “ETOX,” and new variables are assigned “ETOX” as prefix.

#### Process overview and scheduling

The description of the processes and their scheduling is focused on the newly added procedures (detailed in the Supporting Information, Appendix A1). The BEEHAVE_ecotox_ model incorporates three main additions to the original BEEHAVE implementation: water‐foraging, setting experiment starting conditions, and processes to track the pesticide fate for foragers and the hive.

The BEEHAVE_ecotox_ model can track the contact doses of the foragers and the collected pesticides in pollen, nectar, and water as they pass from foragers through the hive. Subsequently it can predict the ensuing effects on each colony's bee cohort and the foragers. In the model, the time step is 1 day. The added procedures run daily. At the beginning of the day, the pesticide concentration in nectar and pollen is calculated. Subsequently, if the conditions for foraging are met, the foragers repeatedly collect nectar and pollen and are exposed to the pesticide via contact, nectar, and water. Unless the pesticide has immediate knock‐down effects, the pesticide is brought back to the hive, where the cohorts are subsequently exposed. At the end of the day, doses received by the individuals are summed, and the subsequent effects are calculated. Finally, the ecotox‐specific plots are updated on the graphical user interface (GUI).

Apart from the possibility of simulating exposure and effects on survival, functionality to start the simulation on any day of the year with a predefined colony structure was added. This is particularly useful to simulate colonies from semifield or field studies.

#### External exposure module

The external exposure module incorporates the concentration of pesticides in the bee‐relevant matrices such as nectar, pollen, and water. This is achieved by taking into account the start and duration of exposure (e.g., via a semifield study with the tunnel setup), the application rate, and the dissipation of the pesticide in the whole plant (DT50), which are given as input parameters on the GUI.

The concentration of the pesticide (µg/kg) in nectar and pollen should be calculated before the model simulation and used as input parameters. In the present study, we used the 90th percentile residue/unit dose (RUD; mg/kg; EFSA, 2013) to recalculate a concentration in nectar and pollen based on the application rate (kg/ha). Should additional data become available, the RUDs for nectar and pollen can be revised later without the need to change anything in the ecotoxicological module of the BEEHAVE model.

When foragers forage on pollen, nectar, or water, they receive an oral dose of the pesticide. The oral dose of the foragers is an emergent property of the model and a function of the number of trips, flight distance, concentration in the respective matrices, and their actual consumption. Foragers can also be exposed via contact on the day of application. The contact exposure is calculated based on application rate (kg/ha) and RUD for foliar insects from the EFSA Bird and Mammal Guidance Document (EFSA, 2009) to convert the received dose into mg/kg of insects. This value of the RUD is conservative because most of the foliar insects are smaller than bees. Smaller insects have a larger surface area to volume ratio, which can lead to higher exposure concentrations. The calculated dose (mg/kg) is then multiplied by forager weight (0.1 g) to calculate the dose/bee in µg. Depending on the substance, contact exposure could lead to immediate knock‐down or mortality of the foragers. In this case, the foragers do not return to the hive. Therefore, immediate forager mortality can be activated or deactivated on the GUI. If immediate forager mortality is deactivated, foragers bring back the pesticide into the hive through the collected nectar, pollen, and water and can die at the end of the day. Collected pesticide is then processed by the in‐hive fate module and leads to exposure of in‐hive adult bees and larvae.

#### Water‐foraging module

The water‐foraging module is incorporated in the foraging module of BEEHAVE and follows the same steps as foraging for nectar and pollen. This module can be switched on or off depending on the research question. Due to a lack of experimental data, this module has not been rigorously validated and thus is turned off in the default model settings. In general, the first step is the calculation of water demand. Water can be needed either to cool the hive or to dilute the honey for feeding (Johansson & Johansson, 1978; Lindauer, 1954, 1955; Nicolson, 2009; Southwick & Heldmaier, 1987). The required water for cooling is calculated as a function of the daily environmental temperature, hive measurements, and material characteristics. The calculations are based on the approach of Lindauer (1954) and are described in more detail in the Supporting Information, Appendices A1 and A8. The amount of water needed for honey dilution is calculated based on the anticipated daily honey consumption and the necessity to increase the water content in honey from 20% to 50% (Hooper, 2010; Knopper et al., 2016). The number of foragers required to cover the water demand to cool or dilute is defined before each foraging round. Water foragers then search for water sources and share information about their location in a way similar to the communication of nectar location by nectar foragers. Handling time, duration, cost of both successful and unsuccessful foraging flights, number of trips made each day, and background risk are recorded by the module. The water source is characterized by the volume available during a day and by the concentration of the pesticide if present. The concentration in the water contributes to the forager oral exposure. Ten percent of the pesticide concentration in water is added to the oral exposure of the forager carrying it (Gary & Lorenzen, 1976). The other 90% contributes to the pesticide concentration in honey during its dilution and subsequently to the oral exposure of the in‐hive cohorts through feeding.

#### In‐hive fate module

The in‐hive fate module simulates the entry and distribution of the pesticide in the hive. Honeybees mature honey by evaporating water from the nectar for up to 5 days and then storing the concentrated honey in capped honey cells (Seeley, 1995; Winston, 1991). Because consumption is driven by energetic content rather than volume of nectar, and to avoid having to simulate the evaporation process, the pesticide concentration in nectar is converted into µg/kJ (detailed conversion is described in the Supporting Information, Appendix A1, 7.2. Updates). The maturation is represented in BEEHAVE_ecotox_ by dividing the honey stores into five compartments (from day 0 to day 4). Brought‐in nectar enters the day 0 store and what is not consumed by the end of the day enters the day 1 store. What is left after day 1 enters the day 2 store, which is continued until the end of day 4, when it enters the capped honey store. By this procedure the dose is transferred from one daily compartment to the next until it reaches the capped honey stores. In reality, nectar is often consumed from matured honey cells, which leads to dilution of the pesticide entering with fresh nectar after application (Winston, 1991). However, to follow a conservative approach, the bees were given preference for fresh honey. The bees will first meet their energetic needs by feeding from the day 0 store, and only when this store is empty do they start to feed on the store of day 1, then day 2, and so on. In this way, exposure is maximized on the day of application and in the immediate aftermath whereas prolonged low exposure occurs in the honey stores. When the nectar enters the stores, it had to be decided how much it is mixed within each store, for example, by adding more subcompartments. The modeling study by Rumkee et al. (2017) explored how mixing nectars with different pesticide concentrations in nectar cells on the day of application affected responses. These authors found that under most conditions, full mixing leads to the worst‐case scenario. Therefore, stores were not subdivided further in the BEEHAVE_ecotox_ model. Colonies have much smaller pollen stores compared with nectar. Therefore, the pollen store has not been compartmentalized, and pesticide brought into the hive via pollen immediately contributes to the concentration in pollen across the whole hive. When bees consume the nectar and pollen, the active ingredient is removed from the stores, which is referred to as “biological dissipation” (Rumkee et al., 2017). For this reason, chemical dissipation from the honey should not be simulated, unless data are available that allows for separation of biological and chemical dissipation. Thus, the default setting does not assume chemical dissipation after the nectar enters the hive.

#### Effect module

The effect module for survival covers mortality as a result of exposure for the different cohorts. Whereas foragers can be exposed to pesticides outside of the hive, the in‐hive bees and larvae can only be exposed via food. The effect module also incorporates the possibility of considering the filtering effect of the nurse bees. For some pesticides, nurse bees have been found to pass less of the pesticide concentration to the larval food, via hypopharyngeal secretions than they have taken up (Böhme et al., 2018; Davis & Shuel, 1988). This “filter function” can be activated on the GUI if supporting data are available. Both the contact and the oral toxicity are implemented via dose–response relationships from standard regulatory studies. The effect module uses the slopes and median lethal dose (LD50) values of standard acute contact, oral, chronic oral, and larval studies as inputs and is therefore simple to adapt for other compounds.

To calculate the input parameters LD50 and the slope of the dose–response relationship for adult bees, the honeybee acute oral (OECD, 1998a) and chronic oral tests (OECD, 2017) can be used. Test data need to be translated into daily exposure and total oral dose to be used to assess daily survival. This is straightforward for the acute oral test in which a single dose is given within 6 h and the bees are observed for 48 h. In the present model, the dose–response relationship after 48 h is used. For the chronic oral test, the bees are exposed continuously over 10 days, so scaling to daily mortality is needed. Between parameters from acute and chronic exposure, the preference is given to the most conservative dose–response relationship (for more details see the Supporting Information, Appendix A10).

The contact exposure parameters can be calculated from honeybee acute contact tests (OECD, 1998b). Test data are scaled to daily exposure contact dose and daily effect, which is straightforward because the bees are exposed only to a single dose and observed for 48 h.

To calculate the input parameters LD50 and the slope of the dose–response relationship for larvae, the honeybee larval toxicity test with repeated exposure (OECD, 2016) can be used. For model input, an estimation of daily exposure and daily effect is required. Therefore, the mortality at the end of 7 days (larval phase) is scaled to a daily mortality. If the mode of action indicates an effect in a development stage after the first 7 days, as with fenoxycarb, survival data after 22 days can be used. Although pupae do not feed, larval mortality affects their numbers as well.

### Model testing

#### Verification of the code

The code was thoroughly checked during model development. Similar to the original BEEHAVE, the plots on the interface were used to monitor the model behavior, for example, by tracing the fate of a pesticide in nectar, pollen, and water into the hive store. The dose–response relationships implemented in the effect model were verified separately in Excel spreadsheets (Supporting Information, Appendix A3). Special attention was also given to the consistency of units because the model applies both parameter values common for ecotoxicological and bee population studies. The complete code was scrutinized independently by two co‐authors not involved in the original model development and coding.

#### Sensitivity analysis

A sensitivity analysis was conducted following the Becher et al. (2014) approach assessing the sensitivity of the model output to changes in the newly implemented parameters (in total 19). Both the sensitivity analysis and the model validation were conducted for the two common toxic standards with different modes of action (see the *Model validation* section for details). The settings and detailed results can be found in the Supporting Information, Appendices A4 and A5.

#### Model validation

For the validation of BEEHAVE_ecotox_, semifield studies with the tunnel setup (“tunnel studies”) were chosen. Tunnel studies were chosen for validation because they combine controlled exposure and relatively free foraging (confined within the tunnel) and thus were deemed best for validating effect sizes (OECD, 2014; European and Mediterranean Plant Protection Organization, 2010; Pistorius et al., 2012). In addition, these study types provide detailed information in terms of adult and brood mortality and colony composition (adults, brood, nectar/honey, and pollen stores). For the validation, two tunnel studies from Germany were selected, which were conducted in July to September 2012 and July to August 2016 (Bayer, 2019; Syngenta, 2012). Each study was conducted with a control and a different substance, representing two different modes of action: dimethoate and fenoxycarb. In both studies, three (dimethoate) and four (fenoxycarb) hives were each placed separately in tunnels with flowering *Phacelia* for 9–14 days for the exposure phase, before being moved to untreated monitoring sites with a total observation time of 43–53 days (corresponding to two brood cycles after the start of exposure).

The organophosphorus insecticide dimethoate is an anticholinesterase that affects adult bees through both contact and oral exposure (Christen et al., 2019). Fenoxycarb acts as a juvenile hormone analog and affects molting. Therefore, it is mainly toxic to brood, particularly seen as pupal mortality (Aupinel et al., 2007). The two different modes of action were chosen to test whether BEEHAVE_ecotox_ predicts the initial effects, but also subsequent impacts on colony dynamics. Both substances were applied as spray application on *Phacelia* sp. at peak flowering with 0.4 kg a.i./ha for dimethoate and 0.3 kg a.i./ha for fenoxycarb. Exposure via water was not included because the bees were given clean water in the tunnels.

The BEEHAVE_ecotox_ was set up to match the conditions of the respective tunnel study. The model was initialized to match the forage availability in the tunnel settings, application rates of the respective substances, and the hives' conditions (including colony structure: numbers of adult bees and brood, and levels of honey and pollen stores). Furthermore, the relocation of the hives into and out of the tunnel was simulated. The ecotoxicological data for both substances were derived from the study reports from contact, acute and chronic oral, and larval studies (data and details in Supporting Information, Appendix 10).

To validate the output of the model, the simulated status of the colonies and their stores (mean values of 10 model runs) were compared with the colony assessment data from the tunnel studies. The results were analyzed by the absolute numbers of adult bees and brood and in relation to control. Specifically, to estimate the magnitude of effect predicted by BEEHAVE_ecotox_ (treatment with a pesticide relative to the control), the model predictions were compared with the simulation runs with the same starting colony structure and food store conditions but without the application of the pesticide. A detailed report on the model validation can be found in the Supporting Information, Appendix A4.

## RESULTS

The sensitivity analysis showed that the concentration of the pesticide in nectar and pollen (external exposure module) and the LD50 for adult bees and larvae (effect module) had the largest influence on the population dynamics of adult bees (for more details, see the Supporting Information, Appendix A2).

The main purpose of the validation was to test whether BEEHAVE could predict the initial effect sizes and long‐term impact those initial effects had on colony dynamics for two highly bee‐toxic pesticides with different modes of action, namely, acute mortality (dimethoate) and brood mortality (fenoxycarb).

### Colony strength relative to control

**Figure 2:**
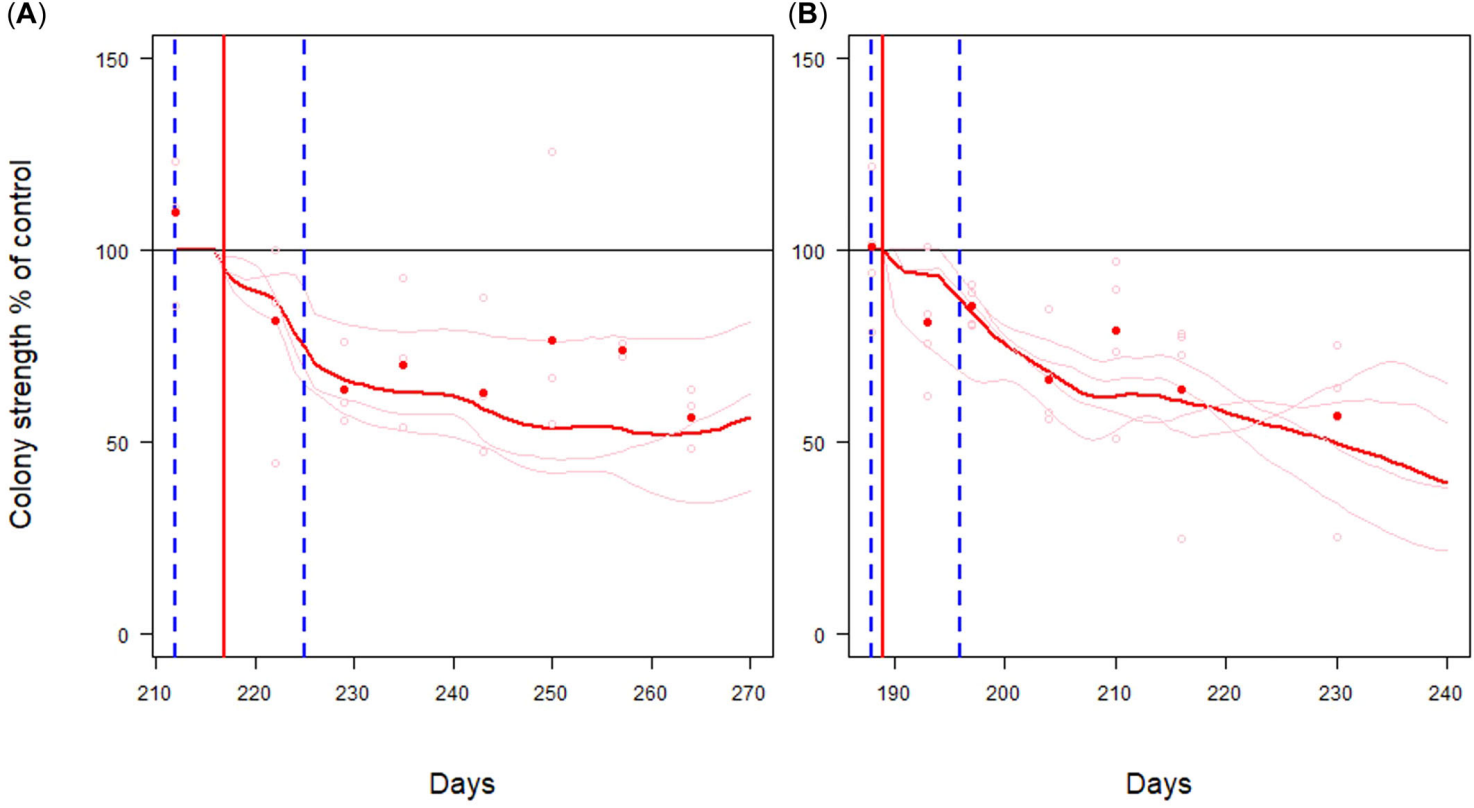
Relative effect sizes in percentage of control (100%‐line) of colony strength (i.e., sum of all cohorts: eggs, larvae, pupae, inhive bees, foragers) in the experiments with dimethoate (**A**) and fenoxycarb (**B**). Red solid points are mean empirical data of the tunnel experiment; red lines are mean model predictions. Pink points are empirical data of the tunnel experiment for each of three (dimethoate) and of four (fenoxycarb) separate hives; red lines are model predictions for each separate hive averaged over 10 runs. Dashed vertical blue lines are delineating the start and the end of the tunnel phase (exposure phase). Vertical red lines indicate the day of the application.

The validation against two semifield studies showed that the BEEHAVE_ecotox_ model captured the initial effects on colony strength (total numbers of adult bees and brood) and the subsequent colony dynamics well for both dimethoate and fenoxycarb despite their very different modes of action (Figure 2). The model predicted the relative magnitude of effects at colony level directly after application, as well as the long‐term reduction in colony strength at the untreated monitoring site during the postexposure phase for both substances. Furthermore, the lack of recovery of the colonies within the observed study duration of 43–53 days was predicted by the model.

For dimethoate, the model was able to predict the relative reduction in colony strength of approximately 20% directly after application in comparison with the control (Figure 2A). The long‐term decline in colony strength after exposure was also captured and slightly overpredicted. At the end of the observation period (two brood cycles after pesticide application), the model prediction of 48% reduction in colony strength was in line with the empirical data (44%). Results were similar for fenoxycarb (Figure 2B). Although the model slightly underestimated the initial reduction in colony strength relative to the control, the long‐term reduction in colony strength in the postexposure phase matched the empirical data well (Figure 2B). The model prediction of 50% colony strength reduction was conservative compared with the 44% recorded at the end of the study.

### Colony strength—absolute numbers

**Figure 3:**
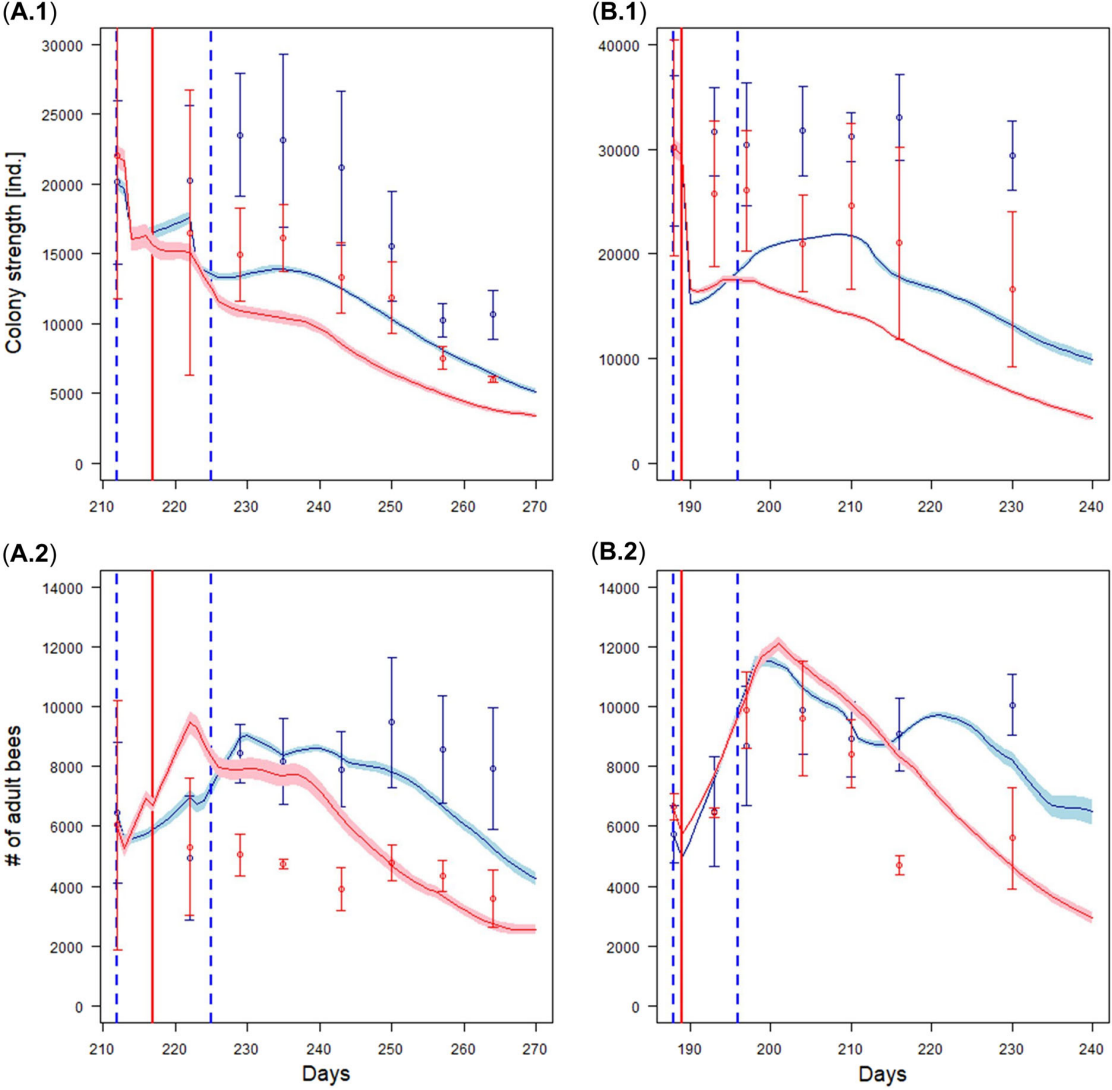
Colony dynamics in number of bees for dimethoate (**A**) and fenoxycarb (**B**). Plots A.1 and B.1 show the colony strength (all cohorts) over time, A.2 and B.2 show the number of adult bees. Dots are the empirical data from the semifield study with the standard deviation; lines are the predicted values as the mean of three (dimethoate) and four (fenoxycarb) beehives with the 95% confidence interval (*n* = 10). Blue dots (lines) are control and red dots (lines) are pesticide treatment. Dashed vertical blue lines are delineating the start and the end of the tunnel exposure phase. Vertical red lines are the day of the application.

Although the relative effect sizes for colony strength were captured well, the absolute numbers were less accurate (compare Figures 2 and 3). The lack of accuracy was mainly caused by the control dynamics not being captured in full, especially the brood dynamics (see the Supporting Information, Appendix A4). Thus, BEEHAVE_ecotox_ underpredicted the control colony strength for both studies (Figure 3A.1 and B.1), and this was reflected in the treatment simulations, where BEEHAVE_ecotox_ also underpredicted the colony strength of the dimethoate and fenoxycarb treatments (Figure 3).

The colony dynamics after the tunnel exposure phase were captured better albeit conservatively by BEEHAVE_ecotox_. Thus, BEEHAVE_ecotox_ predicted the lack of recovery of both the dimethoate and fenoxycarb treatment colonies (Figure 3A.1 and B.1).

### Adult bees—Absolute numbers

The number of adult bees in controls was matched better by BEEHAVE_ecotox_ than the overall numbers for the colony strength (Figure 3A.2 and B.2). For the dimethoate treatment, although the number of adult bees in and immediately after the tunnel exposure phase was overpredicted, the long‐term effects leading to a steady decline were matched well (Figure 3A.2). The predicted effects of the fenoxycarb treatment on the number of adults were more accurate than for dimethoate and overall matched well except for a single time point (Figure 3B.2). The predicted increase in the number of adult bees after the tunnel exposure phase followed by a decrease matched the study well (Figure 3B.2).

**Figure 4:**
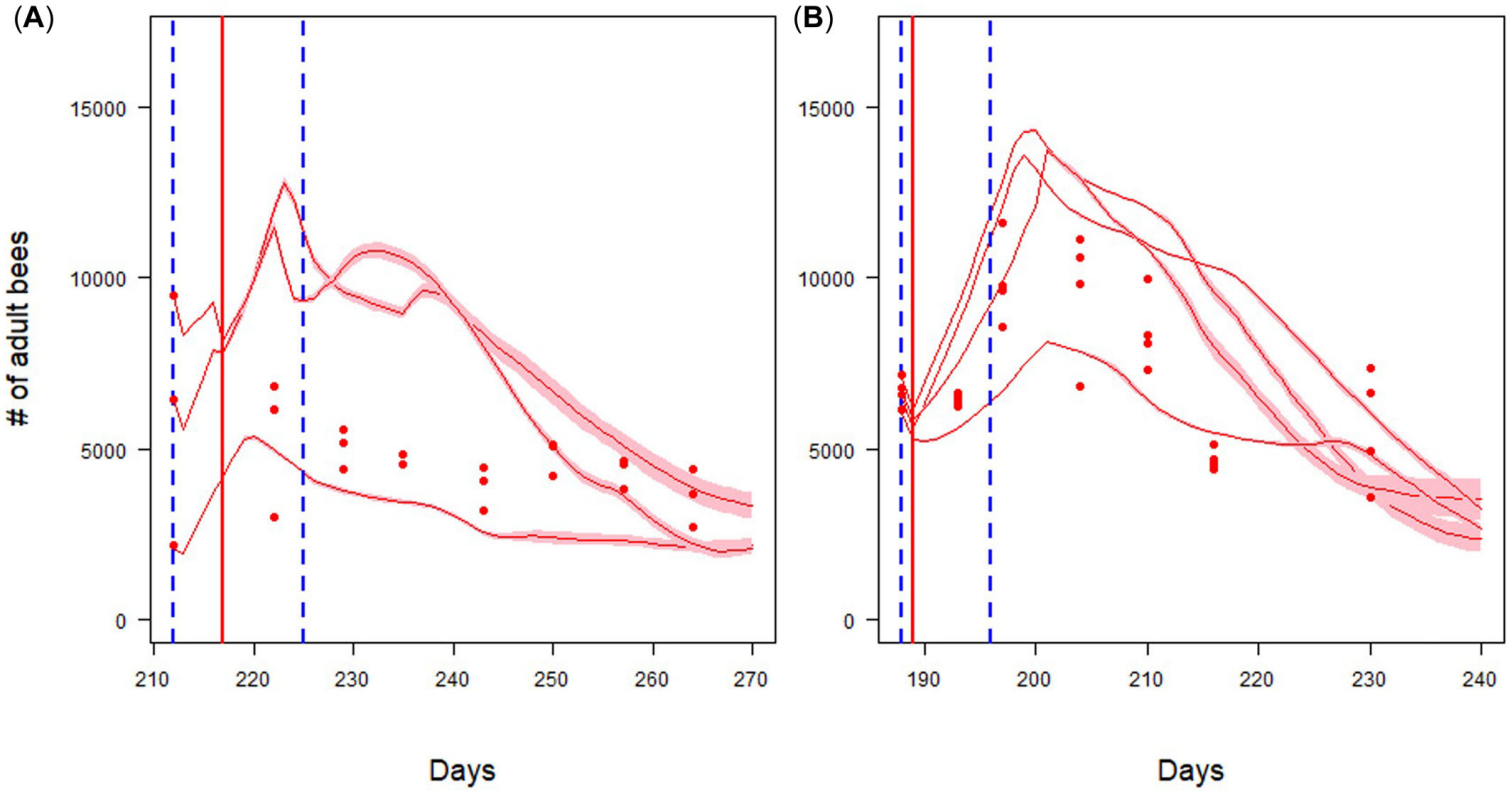
Number of adult bees over time of three individual hives of the treatment with dimethoate (**A**) and of four individual hives in the treatment with fenoxycarb (**B**). Dots are the empirical data from the semifield study; lines are the mean predicted values with the 95% confidence interval (*n* = 10). Dashed vertical blue lines are delineating the start and the end of the tunnel exposure phase. Vertical red lines are the day of the application.

A more detailed analysis showed that the overprediction of the absolute numbers of adult bees in the dimethoate simulations was found in two of the three hives, but not in the third (Figure 4A), showing that there is natural variability between replicates in studies and also between replicates in the simulations. For fenoxycarb, all four simulated colonies showed matching dynamics to those measured in the semifield study (Figure 4B). A more detailed description of the validation can be found in the Supporting Information, Appendix A4.

## DISCUSSION

The BEEHAVE_ecotox_ model was developed, verified, and validated to realistically assess the risk of pesticides applied to bee‐attractive flowering crops present to foraging bees. The model mechanistically links exposure in different compartments in the landscape with the foraging behavior of bees. The model was successfully parameterized using standard regulatory studies for two bee‐toxic pesticides. The sensitivity of BEEHAVE_ecotox_ to 19 parameters was analyzed to assess the influence on the bee colony dynamics. Finally, the model was validated with two semifield tunnel studies. Thereby, BEEHAVE_ecotox_ closes a critical gap for the application of BEEHAVE in the risk assessment for bees previously identified by the EFSA (2015, 2021).

In BEEHAVE_ecotox_, potential exposure to a pesticide is simulated via nectar, pollen, water, and contact, and the model predicts effects on all cohorts. This was done by combining several additions to BEEHAVE that have been developed since BEEHAVE was first published: landscape aspects (Becher et al., 2016), lethal and sublethal effects on different cohorts (Rumkee et al., 2015; Thorbek, Campbell, Sweeney, & Thompson, 2017), effects of bee behavior on exposure and “biological dissipation” (Rumkee et al., 2017), and validation studies (Agatz et al., 2019; Schmolke et al., 2020). However, with a mechanistic link between exposure and effects, cohort‐specific exposure does not need to be imposed as conducted so far (Henry et al., 2017; Rumkee et al., 2015; Thorbek, Campbell, Sweeney, & Thompson, 2017; Thorbek, Campbell, & Thompson, 2017). Instead, it emerges from the model in combination with the environmental scenario (Rico et al., 2016). The emergent property of the mechanistic model helps us to understand and predict how pesticides and multiple stressors impact colonies in a system‐based approach (EFSA et al., 2021). Another important point was the addition of a water‐foraging module to enable simulation of exposure from guttation droplets and puddles in the field. Moreover, the model can distinguish between fast‐ and slow‐acting pesticides. At high levels of exposure, a fast knock‐down effect would make it less likely for foragers to return to the hive with contaminated nectar and pollen due to immediate knock‐down/mortality. On the other hand, at the same level of exposure, slower acting chemicals are more likely to reach in‐hive bees and brood through contaminated forage.

The methods to estimate exposure in the environment for contact and in nectar, pollen and water were taken from EFSA guidance documents (EFSA, 2009, 2013; FOCUS Ground Water Work Group, 2014). In the model, the foragers collect nectar, pollen, and water, which are then distributed via the in‐hive fate module so that all cohorts are exposed according to their dietary requirements for nectar and pollen. The fate module simulates “biological dissipation” (sensu Rumkee et al., 2017). Therefore, chemical dissipation inside the colony should not be included in risk assessment scenarios unless data are available. The data should allow the separation of biological and chemical dissipation. The effect module translates endpoints from standard ecotoxicological studies (see OECD, 1998a, 1998b, 2009, 2016, 2017) into dose–response relationships, which work with the same entities and units as BEEHAVE. Because BEEHAVE_ecotox_ and ecotoxicological study results have different units and timescales, it is important that the exposure and toxicity parameters of the studies be transformed beforehand to match the model requirements.

The honeybee ecotox module was implemented within BEEHAVE, resulting in the BEEHAVE_ecotox_ model, but due to its modular approach, the module can be integrated into other mechanistic honeybee models. Because the BEEHAVE model has already been extensively validated (Agatz et al., 2019; Becher et al., 2014; Schmolke et al., 2020), the focus in the present study was on validating the added ecotoxicological functionality, that is, the predicted effect sizes on the different honeybee cohorts. The validation case studies with two bee‐toxic pesticides with contrasting modes of action (dimethoate: acute adult toxicity and fenoxycarb: brood toxicity) demonstrated that when these exposure and effect modules were combined with realistic environmental scenarios, BEEHAVE_ecotox_ predicted the initial relative effect sizes well. The model also conservatively captured the substantial long‐term effects on colony dynamics and the lack of recovery of the colonies.

Variability in BEEHAVE was lower than in the experimental data, as shown already in previous validation studies (Agatz et al., 2019; Schmolke et al., 2020). This lower variability is expected and desirable for a mechanistic model in which parameters were selected to represent a typical hive and thus predict the central tendency.

The BEEHAVE_ecotox_ model captured both the relative effect sizes and the lack of colony recovery well if starting conditions of the hive for control and treatment simulations were identical (Figure 2). The absolute numbers of eggs, larvae, and pupae were less accurately predicted for both control and treatments (Figure 3). This deviation is also one of the driving factors for deviating colony strength predictions. There were three main reasons for this. First, the area and availability of forage in tunnel studies is much less than what freely foraging bees in the field would encounter. This leads to an acute shortage of especially pollen in the BEEHAVE colonies. It appears that BEEHAVE colonies react more drastically to this pollen shortage than real colonies. Schmolke et al. (2020) also found that the pollen budget in the original BEEHAVE does not represent realistic consumption; they obtained better predictions with pollen consumption from the USEPA (2014) collated data. In real colonies, foragers only consume a negligible amount of pollen. However, in BEEHAVE, in‐hive bees, foragers, and winter bees consume the same average amount of pollen (Becher et al., 2014), which may have exacerbated the pollen shortage in the simulations relative to the tunnel studies. Although BEEHAVE may overestimate the pollen demands of the colony, BEEHAVE_ecotox_ aimed to change as little as possible of the bee ecology in the original BEEHAVE model. Because the BEEHAVE_ecotox_ underpredicted the colony strength, it was deemed fit for purpose for risk assessment because of its conservatism. Additional analyses on the effect of forage availability can be found in the Supporting Information, Appendix 9.

Second, BEEHAVE is sensitive to the initial conditions of the hives (Agatz et al., 2019; Schmolke et al., 2020), especially to the age structure of the colonies. In the tunnel studies, there is some uncertainty in the pre‐exposure assessments because it is not possible to estimate the age of the bees and, therefore, whether they are in‐hive bees or foragers. Model runs were based on an even age distribution, which might have skewed the results. The publication by Rumkee et al. (2015) highlighted that BEEHAVE is sensitive to the balance between brood and in‐hive bees. In the model, each in‐hive bee can only handle three brood cells. Consequently, when there are too few in‐hive bees, the brood is terminated, and egg‐laying is reduced. In real hives, there may be more plasticity so that the transition from nurse bee to forager is delayed, or nurse bees can care for more than three brood cells when necessary. The brood termination and pollen budgets in BEEHAVE_ecotox_ and BEEHAVE can be improved when more data became available. Appendix 9 in the Supporting Information provides more analyses of the effects of age structure and in‐hive/forager ratio.

Third, the landscape composition and forage availability in the postexposure phase of the studies were not described in detail. Thus, these parameters had to be estimated, with some degree of uncertainty, which might have contributed to further skewing of the results (see also the Supporting Information, Appendix 9). Nonetheless, the purpose of the validation was to test whether the exposure, fate, and effect modules in the BEEHAVE_ecotox_ could predict initial effect sizes and subsequent dynamics of the colonies. This was successfully and conservatively captured. The BEEHAVE_ecotox_ water‐foraging module, which allows for assessment of the risk of guttation droplets for systemic pesticides or puddles, could not be validated because suitable data were not available.

Different validation studies, as well as sensitivity analyses, of BEEHAVE have shown that environmental scenarios have a large impact on initial pesticide effects and colony resilience (Agatz et al., 2019; Becher et al., 2014; Henry et al., 2017; Rumkee et al., 2015; Schmolke et al., 2020; Thorbek, Campbell, Sweeney, & Thompson, 2017; Thorbek, Campbell, & Thompson, 2017). Even though scenarios for validation will have to be adapted to match the experimental conditions, a set of environmental scenarios based on stakeholder agreement is crucial for a standardized use in risk assessment. These scenarios should include weather, nectar and pollen availability, size, and fragmentation of the landscape. Another important factor is the beekeeping practice, which can reduce or increase the resilience of the honeybee colonies (see Abi‐Akar et al., 2020; Schmolke et al., 2020). The ESFA has recently assessed a large number of control colonies from field studies conducted across Europe, applying the original BEEHAVE model (EFSA et al., 2021). They proposed a strategy to standardize the calculations of some of the above‐mentioned environmental factors affecting colony dynamics. Application and further development of the presented strategies might be useful for the development of standardized simulation scenarios. To use a model to inform decision support, it is important to understand the domain of applicability. Thus, when comparing models for decision support, it is crucial to take the purpose of the models into account; that is, does it fall into the category of demonstration, understanding, or prediction (Grimm, Johnston, et al., 2020). For honeybee risk assessment, a model that can predict exposure, effects, and colony dynamics under field conditions with a good level of accuracy is needed. Although different bee models are available, their purpose is not always within the scope of the risk assessment. Besides the BEEHAVE model, the VarroaPop+Pesticide model (based on BEEPOP; DeGrandi‐Hoffman et al., 1989; Kuan et al., 2018; Minucci et al., 2021) has been continuously developed and is the only colony model with validation efforts to rival those that have gone into BEEHAVE (see Minucci et al., 2021). Minucci et al. (2021) validated the VarroaPop+Pesticide model against colony feeding studies. They demonstrated that overall effects were captured well, but effects were underestimated at low exposure levels. The authors concluded that this was likely caused by unknown toxic effects; however, it could also have resulted from the model having higher resilience than real colonies. In colony feeding studies, such as the ones Minucci et al. (2021) used for validation, the pesticide concentration and consumption are known, and effects can be enforced. However, for freely foraging colonies, the exposure will emerge from an interaction between concentration in the treated crop, the relative attractiveness of the treated forage compared with other forage sources, foraging activity, and how the pesticide is distributed through the colony. To our knowledge, only the BEEHAVE_ecotox_ module can predict in‐hive exposure and ensuing effects on all cohorts.

## CONCLUSIONS

The BEEHAVE model was constructed in a modular fashion with the landscape, colony, varroa & virus, and foraging modules. The ecotoxicological module was added to BEEHAVE to mechanistically predict pesticide exposure to and effects on all cohorts. The validation against tunnel studies showed that BEEHAVE_ecotox_ captures the initial effects sizes and long‐term effects on colony dynamics well. The ecotoxicological modules are portable, and the principles and algorithms can also be applied in other honeybee colony models. We recommend that any colony model to be used for honeybee risk assessment should be validated (as BEEHAVE and BEEHAVE_ecotox_ have been) against field studies, colony feeding studies, and tunnel studies. Only by comparing such validations can the model that best fits the risk assessment problem formulation be identified.

## Supporting Information

The Supporting Information is available on the Wiley Online Library at https://doi.org/10.1002/etc.5467.

## Disclaimer

Thomas G. Preuss and Vanessa Roeben work for Bayer. Jack Rumkee works for Syngenta. Pernille Thorbek works for BASF SE. Annika Agatz, Benoit Goussen, and Liubov Zakharova work for the Institute for Biological Analytics & Consulting. Bayer, BASF SE, and Syngenta produce and sell agrochemicals. All authors have an interest in getting BEEHAVEecotox accepted for regulatory purposes.

## Author Contributions Statement

**Thomas G. Preuss**: Conceptualization; Data curation; Investigation; Validation; Project administration; Writing—original draft; Writing—review & editing. **Pernille Thorbek**: Conceptualization; Methodology; Project administration; Writing—original draft; Writing—review & editing. **Jack Rumkee**: Methodology; Validation; Visualization; Writing—review & editing. **Annika Agatz**: Methodology; Project administration; Writing—review & editing. **Liubov Zakharova**: Project administration; Validation; Writing—review & editing. **Vanessa Roeben**: Project administration; Validation; Visualization; Writing—original draft; Writing—review & editing. **Benoit Goussen**: Writing—review & editing. All authors contributed critically to the draft and gave final approval for publication.

###  

This article has earned an Open Materials badge for making publicly available the digitally shareable data necessary to reproduce the reported results. The data are available at https://github.com/ibacon-GmbH-Modelling/BEEHAVEecotox. Learn more about the Open Practices badges from the Center for Open Science: https://osf.io/tvyxz/wiki.

## Supporting information

This article includes online‐only Supporting Information.

Supporting information.

Supporting information.

Supporting information.

Supporting information.

Supporting information.

Supporting information.

Supporting information.

Supporting information.

Supporting information.

Supporting information.

Supporting information.

## Data Availability

All data generated and analyzedduring the present study are included in either the articleitself or the Supporting Information.
