# Supplementary material for: The BEEHAVEecotox Model—Integrating a Mechanistic Effect Module into the Honeybee Colony Model
Source: Environ Toxicol Chem. 2022 Oct 4;41(11):2870–82. doi: 10.1002/etc.5467 (PMC9828121; doi:10.1002/etc.5467)
Supplement: Supplementary file 1 — Supporting information. [file ETC-41-2870-s002.pdf]

APPENDIX 1 - BEEHAVE$_{ECOTOX}$ MODEL

The description of the model is following the ODD protocol (Overview, Design concepts and Details) by Grimm et al. (Grimm et al., 2006, 2010, 2020). The BEEHAVE$_{ecotox}$ model is based on the BEEHAVE model (BEEHAVE_BeeMapp2016; available at http://beehave-model.net/download/) and introduces new procedures to the original model to account for the exposure and effect of plant protection products. The description of the model is complimentary to the ODD protocol published with the original version of BEEHAVE (Becher et al., 2014) and only describes the applied changes. BEEHAVE$_{ecotox}$ was implemented in NetLogo, version 5.3.1 (Wilensky, 1999).

1. Purpose

Bees are considered important pollinators providing a vital ecosystem service (EFSA, 2013). As such, bees are potentially exposed to plant protection products when foraging in agricultural landscapes. Here, modelling can offer a powerful tool to investigate the complex interactions of these social insects within their colony and with their environment. Different honeybee colony models are available of which the BEEHAVE model has been extensively reviewed by EFSA (Becher et al., 2014; EFSA, 2015). The BEEHAVE model is a honeybee colony model exploring how various stressors, including varroa mites, virus infections, but also changes in landscape structure and dynamics, affect managed colonies of honeybees. BEEHAVE has also been used to model effects of plant protection products on different cohorts but so far it lacked a mechanistic integration of exposure and effect.

Therefore, the purpose of the BEEHAVE$_{ecotox}$ model is to mechanistically integrate exposure and ecotoxicological procedures into the BEEHAVE model, which can be parameterised with common ecotoxicological regulatory studies and exposure (residue) studies, such as the bee acute toxicity tests for oral and contact exposure (OECD, 1998a, 1998b), the bee larvae toxicity test for repeated exposure (OECD, 2016), and pesticide residue studies (OECD, 2009; Croft et al., 2018). This module introduces minor changes to the BEEHAVE model; specifically, the addition of water foraging, and a simple time-based compartmentalised storage procedure for nectar to allow the incorporation of required ecotoxicological processes into BEEHAVE.

2. Entities, state variables and scales

The ecotoxicological module is an addition to the BEEHAVE model (Becher et al., 2014) and as such, all entities, state variables, and scales from the original BEEHAVE model are included. The description is focussed on the added entities, state variables, and scales which are marked with the prefix ETOX. Original BEEHAVE entities, state variables, or scales are only mentioned when necessary to describe the new ones.

The new state variables were added to the colony and the foraging modules of the original BEEHAVE to simulate exposure. This exposure emerges depending on what matrix the pesticide is found in (nectar, pollen, water, or contract) and foraging activity. The added variables track the pesticide concentration in carried nectar [µg ai/kJ], pollen [µg ai/g], and water [µg ai/g] load as well as a mass of this load. The pesticide concentration in nectar is expressed in mass per energy as consumption of nectar is controlled by energy content instead of total volume. With this approach evaporation of water from nectar does not need to be modelled. Other new state variables track the consumption of the exposed nectar, received dose via contact or oral route and determine the sensitivity to the exposure. The dose and sensitivity variables are used in determining the route-specific mortality based on dose-response relationships, which are calculated separately for oral and contact exposure and, thus, have additive effects on the individuals.

2.1. Colony model state variables

Within the colony model, the following state variables have been added to all bee cohorts (in-hive adults, pupae, larvae, and eggs) to model exposure via a daily dose received by contact or oral routes of exposure:

- *ETOX_PPPContactDose* and *ETOX_PPPOralDose*, which track the dose received via contact or oral exposure by the individual on a particular day.
- *ETOX_rdm_survival_contact* and *ETOX_rdm_survival_oral*, which determine the sensitivity of individuals to contact or oral exposure. Values between 0 and 1 are randomly assigned to each individual.
- *ETOX_consumed*, which tracks the daily consumption of PPP exposed nectar [kJ/day].

2.2. Foraging model state variables

foragerSquadron were assigned additional variables to describe the exposure and water foraging. A detailed overview can be found in Table S1.1. Furthermore, water foraging was added as an activity of the foragers (see Table S1.2). The variables of the flower patches were also extended to account for exposure and water foraging (Table S1.3).

Table S1.1. New state variables of the foragerSquadrons

State Variable	Definition [unit/possible values]	Comment
ETOX_PPPNectarDose	Total mass of pesticide [µg] within the forager's carried nectar load.	
ETOX_F_PPPNectarConc	Concentration [µg ai/kJ] of pesticide within carried nectar load.	
ETOX_PPPPollenDose	Total mass of pesticide [µg] within the forager's carried pollen load.	
ETOX_F_PPPPollenConc	Concentration [µg ai/g] of pesticide within carried pollen load.	
ETOX_knownWaterPatch	The last patch visited as a water forager.	
ETOX_WF	Is the forager a water forager?	Boolean
ETOX_PPPWaterConc	The concentration [µg ai/g] of water load carried within the honey stomach of the foragers.	

Table S1.2. New activities for the foragers in the foraging model

Activity	Definition
bringingWater	Successful water foragers, bringing back water.
unsucessfulWater	Unsuccessful water forager, returning empty.
water foraging	Forager is foraging for water.

Table S1.3. New state variables of the flower patches

State Variable	Definition [unit/possible values]	Comment
ETOX_P_PPPNectarConc	Concentration [µg ai/kJ] of pesticide in the nectar of the flower patch.	
ETOX_P_PPPPollenConc	Concentration [µg ai/g] of pesticide in the pollen of the flower patch.	
ETOX_WaterConc_Fullday	Concentration [µg ai/ml] of pesticide in water sources available all day.	This refers to water sources such as ponds and puddles within patch.
ETOX_WaterVolume_Fullday	Volume of water [ml] within sources available all day.	
ETOX_HandlingTimeWater	Time required for foragers to extract water from the patch.	
ETOX_mortalityRisk_water	Risk of foraging for water on this patch.	In terms of time taken to travel to and from the patch.
ETOX_tripDurationWater	The time required to travel to and from the patch.	
ETOX_waterVisitsToday	A counter for the number of visits to this patch for water foraging.	
ETOX_flightcostWater	Energetic cost for travel to and from this patch.	

3. Process overview and scheduling

The main changes to BEEHAVE are the addition of water foraging and processes added to track the contact doses of the foragers and the collected pesticides in pollen, nectar and water as they pass from foragers through the hive and to predict the ensuing effects on each colony bee cohort and the foragers.

Time is presented as ticks, where one day equals one tick. The added procedures run daily: At the beginning of the day, the pesticide concentration in nectar and pollen is calculated. Subsequently, if the conditions for foraging are met, the foragers repeatedly collect nectar,

pollen and water, and therefore also the pesticide and bring them to the hive. At the end of the day, doses received by the individuals are summarized and the subsequent effects are calculated. Finally, the ecotox-specific plots are updated on the graphical user interface.

3.1. Colony model process overview and scheduling

Apart from the possibility to simulate exposure and effects, functionality to start the simulation on any day with a predefined colony structure was added. This is particularly useful for validation where the colony and study initial conditions need to match.

Table S1.4. New processes in the scheduling of the colony model.

Procedure	Description	Run by
InitialConditionsProc	Set up the hive at the start of the experiment.	Setup
CreateCohortsProc	Set up the colony in a specific way based on data for a specific day.	Setup
TExposure_at_patch_ETOX	Calculate pesticide concentration in nectar and pollen in the patch over time.	DailyUpdateProc [run by Go]
TupdateInternalExposureNectar_ETOX*	Calculate the doses received by individuals within the colony through feeding as well as the removal of nectar from the ecotox-specific honey stores.	Go
Tplot_exposureandeffects_ETOX	Plot ecotox-specific plots.	Go

*This process is a part of both colony and foraging models.

3.2. Foraging model process overview and scheduling

The main new functionality keeps track of collected residues of pesticides, accounts for contact exposure, and simulates the exposure the forager experiences from feeding on the honey stores.

Table S1.5. New processes in the scheduling of the foraging model.

Procedure	Description	Run by
TflightCostupdate_ETOX	Calculate energy and time spent on the foraging trip.	Foraging_flightCosts_flightTimeProc [run by ForagingRoundProc]

Table S1.6. New procedures run as a part of TupdateInternalExposureNectar_ETOX* to calculate honey consumption and oral dose for foragers and for in-hive cohorts.

**Procedure**	**Description**	**Run by**
Tfeed_on_honey_stores_foragers_ETOX	Calculate honey consumption and oral dose for foragers.	TupdateInternalExposureNectar_ETOX* and TflightCostupdate_ETOX
Tfeed_on_honey_stores_cohorts_ETOX	Calculate honey consumption and oral dose for cohorts.	TupdateInternalExposureNectar_ETOX*

*This process is a part of both colony and foraging models.

4. Design Concepts

Basic Principles: The ecotoxicological module mechanistically simulates how pesticide residues are passed from the environment (nectar, pollen, and water) to foragers and bees in a colony. This enables colony-level effects to emerge as effects on individual foragers and cohorts are simulated based on the inherent toxicity and received dose. Contact exposure is calculated based on residue per unit dose (RUD) for overspray events (EFSA, 2009) and the application rate of the pesticide in question. The concentration of the pesticide in pollen and nectar is calculated from the application rate by using the residue per unit dose (RUD) for pollen and nectar originated from EFSA (2013). Pesticide-driven daily mortality from the received dose is assessed based on dose-response relationships, where the parameters, such as the LD_{50} and slope, are derived from standard regulatory studies. Details on how these parameters are derived for the different substances can be found in Appendix A10. The contact and oral doses are assumed to have an additive effect on individual mortality, i.e., the numbers of dead bees from each type of exposure are calculated separately and then summarized. The ecotoxicological module also simulates "biological dissipation". Pesticide residues are eliminated from the hive as honey and pollen from the store are consumed by the bees. Lethal effects can then occur due to natural causes or due to exposure. The honey store consists of six compartments with honey of different age, including the last one with capped honey. This compartmentalization ensures different pesticide concentration. As the bees first consume more fresh honey with higher pesticide concentration, that ensures conservative exposure to the pesticide and possible acute effects. Within the compartments, pesticide compounds are fully mixed as this represents the worst case (Rumkee et al., 2017). Exposure via wax, dust, and resin is not currently included,

but can be added. For nectar, the pesticide concentration is recalculated as mass per energy since consumption is driven by energy content.

Emergence: Pesticide exposure of the bees is an emergent property of the module. Exposure is a function of the concentration in the environment, the foraging behaviour, and the actual consumption of nectar, pollen, and water. The effects of the individuals are a function of the received dose and their intrinsic sensitivity. The colony level impact subsequently emerges based on these effects on individuals and cohorts. This includes the feedback from loss of workers on nursing or a lack of brood during exposure leading to a lower adult population later in the year.

Adaptation: The ecotoxicological module does not include any adaptive processes, but the original BEEHAVE does include adaption, for instance to the loss of foragers, brood, or stores.

Sensing: Individuals and cohorts can sense their received dose. Through the added water foraging behaviour, the colony as a whole sense the need for water, which leads to water foraging.

Interaction: Foragers interact with flower patches by taking up nectar, pollen, and water. With food uptake, they also receive a pesticide dose, based on the residue concentration in nectar, pollen, and water and the actual consumption. Within the hive, individuals interact with the food stores and reduce them according to their consumption rate. Therefore, they can be exposed to any pesticide present in these stores.

Stochasticity: Stochasticity is incorporated into the effect calculations. Forager squadrons receive a random floating-point number between 0 and 1, representing their individual sensitivity for lethal effects due to exposure. The level of mortality due to pesticide exposure is calculated using dose-response relationships. If this calculated number is larger than the individual sensitivity, the complete squadron dies. For in-hive adult bees and larvae, this number defines the proportion of the cohorts lost due to toxicity.

Collectives: No additional collectives are added by the ecotoxicological module.

Observation: Additional to the standard BEEHAVE outputs, a set of the plots relevant to the ecotoxicological module were added to the graphical user interface providing daily observations. These include the concentration of pesticide within the flower patches in pollen [µg/kg], nectar [µg/kJ], water [µg/L] and in the in-hive stores of pollen [µg/g] and honey [µg/kJ], with the separation on the compartments for the latter.

Furthermore, the contact doses of the forager squadron and the oral doses received by individual larvae, in-hive bees or by a forager squadron are calculated from the cumulative daily dose of the cohort of interest and plotted on the interface as well. Additionally, the model outputs the mortality as a result of exposure to a pesticide, plotted as the proportion of dead larvae, in-hive bees, and foragers. Supporting plots provide information on the distribution of energy among the honey compartments [kJ], the dynamics of water available at the patches [mL], and the pollination flight activity. Water foraging behaviour is displayed on the interface by two plots: the number of trips occurring per day and the water need of the colony per day.

5. Initialisation

Model simulations can be initialised in the same way as the default BEEHAVE model. In addition, an option to set up the colony based on empirical studies has been added. This allows the definition of a start day for a simulation that diverts from Julian day 1, the duration of a simulation, and the initial conditions of the hive (queen age, total numbers of adults, eggs, larvae and pupae, as well as the food stores in the colony). In addition, some extra options were added to better mimic field and semi-field studies. Thus, the user can alter the swarming conditions for the hive, the baseline adult survival rate, add a decreased survival rate for adults for a stress period, and define a colony feeding and honey extraction schedule. Here, up to nine separate feeding events (day and mass of added fondant) and up to four separate honey extraction events (day of honey harvest and remaining honey in the colony) can be defined.

6. Input data

Input files remain as in the default BEEHAVE model (BEEHAVE_BEEMAPP2016).

7. Submodels and alterations

The following description of the $\text{BEEHAVE}_{\text{ecotox}}$ model follows the structure of the original ODD protocol of the BEEHAVE model (Becher et al., 2014). It is focussed on the new additions by the ecotoxicological module, but also describes necessary alterations of the original BEEHAVE code.

The ecotoxicological module is integrated into the BEEHAVE model in two original parts: the colony and the foraging model.

- The implementation of the external exposure module (Fig. 1) is mainly presented in the colony model (7.2. Update).
- Potential changes in behaviour through the Effect module are integrated into the foraging model (7.II.3. Foraging round), while effects on survival are integrated into both colony and foraging models (7.4. Brood development, 7.6. Adult development, 7.II.2. Forager development and 7.II.3. Foraging round).
- In-hive fate module actions are reflected in the colony model (7.8. Consumption) and the foraging model (7.II.3. Foraging round).
- Introduction of water foraging affects the colony and foraging model as well (e.g., 7.2. Update and 7.II.3. Foraging round).
- The module to simulate experiments is incorporated into the model setup.

I. THE COLONY MODEL

7.1. Time step

Procedures involved:

- StartProc (unchanged)
- Go
- GoTreatmentProc (deactivated in this version)

The procedure *Go* calls two additional procedures: *TupdateInternalExposureNectar_ETOX* and *Tplot_exposureandeffects_ETOX*.

The procedure *GoTreatmentProc* is deactivated in this implementation. Instead, the procedures *InitialConditionsProc* and *CreateCohortsProc* are called in the Setup, allowing to start simulations with specific initial conditions and dates other than January 1st.

Within the procedure *CreateCohortsProc*, the number of eggs, larvae, and pupae are set to the values defined on the graphical user interface. Each of these cohorts have a fixed number of

age classes: three age classes for eggs, six for larvae, and 12 for pupae. The new individuals are split evenly across the age classes, ensuring a uniform age distribution.

The honey and pollen stores of the hives are set on the interface as well. The input is expected in the unit [kg]. Therefore, the usual assessment of number of cells should be converted into [kg] using the conversion factors of Schmickl and Crailsheim (2007) of 0.23 mg pollen per cell and 0.5 mg nectar (honey) per cell.

Furthermore, the total number of adults is defined on the interface. The number of adults is split between in-hive bees and foragers, currently following a fixed ratio of 1:1.

7.2. Updates

Procedures involved:

- CountingProc
- DailyUpdateProc
- TExposure_at_patch_ETOX (new, called in DailyUpdateProc)

Alterations have been made to the procedure *DailyUpdateProc*. The procedure now allows the setting of the start day of the experiment and provides the option to choose higher adult mortality to simulate additional stress in the semi-field studies with tunnel conditions. The variables of the water foraging module (described in detail in the Foraging model) are also set up in this procedure.

Input parameters to define the pesticide exposure by duration, application day, and DT_{50} have been added to the GUI and can be found in Table S1.7.

Table S1.7. Input parameters for the pesticide exposure on the user interface

Description	Variable	Unit
Spin-up phase (number of years the model will run without pesticide exposure)	ETOX_SpinupPhase	[y]
Exposure phase (number of years in which exposure will occur)	ETOX_ExposurePhase	[y]

Application day<br>(the day on which application will occur each year)	ETOX_AppDay	[doy]
Exposure period<br>(the number of days the colony will be exposed to the pesticide)	ETOX_ExposurePeriod	[d]
DT50<br>(dissipation time in which the pesticide is reduced by 50%)	ETOX_DT50	[d]

In the current setup, the ecotoxicological module is working with two flower patches (red and green) defined on the Netlogo GUI.

Flower patches in the model have additionally been given variables for the concentration of pesticide in the nectar, pollen, and water. These values are all set using inputs on the Netlogo interface.

The procedure *TExposure_at_patch_ETOX* parameterises the flower patches in the model. If the current year is within the exposure period and the current day is defined as an application day, the flower patch variables for concentration of pesticide in nectar, pollen and water, and the contact exposure to foragers are set. The contact exposure is calculated based on application rate [g/ha] and residue per unit dose (RUD) for foliar insects from the EFSA Bird and Mammal Guidance Document (EFSA 2009, Appendix 28) to convert the received dose into [mg/kg] of insects. This value of the RUD is considered to be conservative as this data were generated by directly overspray and most of the foliar insects are smaller than bees. Smaller insects have a larger surface area to volume ratio which can lead to higher exposure concentrations. The calculated dose [mg/kg] is then multiplied by forager weight (0.1 g) to calculate the dose per bee in [µg].

The concentration of the pesticide [µg/kg] in nectar and pollen also depends on the application rate [g/ha] and residue per unit dose [mg/kg] from the residue studies (EFSA, 2014). These two concentrations should be calculated before the model simulation and used as input parameters.

If the current day in the model is not an application day but within the exposure period, the pesticide within the plant matrices is reduced according to first order kinetics:

$$P_{t+1} = P_t * e^{\left(-\frac{\ln(2)}{DT_{50}}\right)} \qquad \text{(Eq.1)}$$

where P is the pesticide concentration in nectar, pollen, water, or residue on the plant, and DT_{50} is the DT_{50} of the pesticide as defined on the GUI.

Additionally, the user can limit contact exposure of foragers to only one day with the switch *ETOX_contactexp_oneday*. Therefore, the obtained contact dose is only calculated on the day of application. If the current day is beyond the exposure period, the pesticide concentration for contact exposure is set to zero.

In *TExposure_at_patch* the concentration of pesticide in nectar and pollen and the dose received through contact are set for the current day. Following this, the concentration of pesticide in the nectar is converted from [µg/kg] into [µg/L] (Eq. 2) then [µg/mol] (Eq.3) and finally [µg/kJ] (Eq. 4):

$$ETOX\_PPPNectarconc\_L = \frac{ETOX\_PPPNectarConc\_kg}{(1 - ETOX\_Densityfactor * NectarConcFlowerPatch)} \quad \text{(Eq.2)}$$

where *ETOX_Densityfactor* is 0.1047, a coefficient of the correlation function between sugar concentration in [mol/L] and respective sugar solution density in [L/kg][1]. The nectarConcFlowerPatch is the sugar concentration of the nectar in the current patch [mol/L]:

$$ETOX\_PPPNectarconc\_mol = \frac{ETOX\_PPPNectarConc\_L}{NectarConcFlowerPatch} \quad \text{(Eq.3)}$$

$$ETOX\_PPPNectarconc = \frac{ETOX\_PPPNectarConc\_mol}{(Energy\_sucrose * 1000 * 1000)} \quad \text{(Eq.4)}$$

where the energy of sucrose is given in [kJ/µmol] and converted to [kJ/mol]. After the application day, the pesticide dissipates according to a single first order decay based on the DT_{50} set through the interface.

When a foragerSquadron forages on a flower patch and there is sufficient pollen or nectar available, it is labelled as a “successful forager”. The relevant volume of nectar or weight of

[1] https://www.internetchemie.info/chemie-lexikon/daten/s/saccharose-dichtetabelle.php

pollen is removed from the flower patch, the counters for the number of successful foraging trips are increased, and the foragers activity is set to either bringing nectar or bringing pollen.

If the forager is a nectar forager, its concentration *ETOX_P_PPPNectarConc* is set to the pesticide concentration of the nectar at the flower patch. To calculate the received dose *ETOX_PPPNectarDose*, the concentration is multiplied by the energy of the load currently carried by the forager. The forager then receives 10% of that dose to represent the uptake of pesticide from the carried nectar load (Gary & Lorenzen, 1976). The dose within the carried nectar load is reduced by this amount and the concentration within the nectar is adjusted accordingly. If the forager is a pollen forager, its *ETOX_P_PPPPollenConc* and *ETOX_PPPPollenDose* variables are set to the concentration of pesticide in the pollen of the flower patch and the dose carried in the current pollen load, respectively.

Additionally, the forager is subjected to the contact dose of the flower patch. If the bee has not been previously exposed via contact, the contact exposure is set to the exposure at the visited flower patch. If the forager has already been exposed, the dose can be calculated either as sum of contact doses (*ETOX_ContactSUM* = on) or average of contact doses (*ETOX_ContactSUM* = off)_. This assumption is based on the origin of the underlying dose-response curve: in the acute contact test the bees are exposed for 48 h continuously to a droplet of substance on their back. However, in the field, a more variable exposure is realistic, in which the foragers fly in and out of the field and thus are not exposed continuously. The two approaches sum of dose and average of dose are the two extremes, and the reality is probably found somewhere in the middle. If a compound is taken up fast, then contact exposure should sum up. If a compound is not taken up fast, it stays on the surface of the bees and new contact adds and removes compound from the bee surface, so the problem become a three-phase distribution problem with the surface as mixing phase. In that case it calls more for an average of the doses (for more details see Appendix 11).

Finally, the variables tracking the travelled distance of the foragers are updated.

7.3. Egg laying

Procedures involved:

- NewEggsProc (unchanged)
- WorkerEggLayingProc
- DroneEggLayingProc

Alterations have been made to *WorkerEggLayingProc* and *DroneEggLayingProc* to introduce survival as a result of pesticide exposure.

7.4. Brood development

Procedures involved:

- DroneEggsDevProc (unchanged)
- DroneLarvaeDevProc
- DronePupaeDevProc (unchanged)
- NewDroneLarvaeProc
- NewDronePupaeProc
- NewWorkerLarvaeProc
- NewWorkerPupaeProc
- WorkerEggsDevProc (unchanged)
- WorkerLarvaeDevProc
- WorkerPupaeDevProc (unchanged)

For the development procedures of larvae (*DroneLarvaeDevProc* and *WorkerLarvaeDevProc*), code has been added to introduce pesticide-related mortality effect.

The proportion of individuals that would be lost through pesticide-induced mortality is calculated based on the dose received by the entire cohort.

$$proportion\ lost = 1 - \frac{1}{1 + \frac{Dose}{ETOX\_Larvae\_Oral\_LD50}^{ETOX\_Larvae\_Oral\_SLOPE}} \qquad \text{(Eq.5)}$$

where *Dose* is the oral dose received, *ETOX_Larvae_Oral_LD50* is the LD_{50} value for the pesticide in question, and *ETOX_Larvae_Oral_SLOPE* is the slope of the dose-response relationship. The LD_{50} and the slope are compound-specific parameters set through the interface. The calculated proportion is then removed once per day from the population after background mortality and relevant global counters have been updated.

For larvae, the substance-specific input parameters (LD_{50}, slope) for the dose-response relationship can be derived from the repeated exposure honeybee larval toxicity test (OECD, 2016). For the model input, an estimation of the daily exposure and daily effect is required. Therefore, the mortality after seven days (larval phase) is scaled to a daily mortality. Although, pupae do not feed, larval mortality has a cascading effect on their numbers as well. Details on the derivation of the parameters and temporal scaling can be found in Appendix A10 (Substance profiles).

7.5. Brood care

Procedures involved:

- BroodCareProc (unchanged)

7.6. Adult Development

Procedures involved:

- DronesDevProc
- NewDronesProc
- NewIHbeesProc
- WorkerIHbeesDevProc

The development procedures (*DroneDevProc* and *WorkerIHBeesDevProc*) have also been complemented by a procedure for pesticide-induced lethal effects, similar to the larvae procedure:

$$proportion\ lost = 1 - \frac{1}{1 + \frac{Dose}{ETOX\_Forager\_Oral\_LD50}^{ETOX\_Forager\_Oral\_SLOPE}} \quad \text{(Eq.6)}$$

where *Dose* is the oral dose received, *ETOX_Forager_Oral_LD50* is the LD_{50} value for the pesticide in question, and *ETOX_Forager_Oral_SLOPE* is the slope of the dose-response relationship.

For adults, the substance-specific input parameters (LD_{50}, slope) for the dose-response relationship can be derived from acute oral (OECD, 1998a) and chronic oral (OECD, 1998b) study data. Similar to the calculations for larvae, test data should be translated into daily exposure and total oral dose used to assess daily survival. This is straightforward for the acute

oral test: a single dose is given within 6 hours, and the bees are observed for 48 hours. Thus, the dose-response relationship after 48 hours is used. For the chronic oral test, the bees are exposed continuously over 10 days, so scaling to daily mortality is needed. Between parameters from acute and chronic exposure, the preference should be given to the more conservative dose-response relationship. Details on the derivation of the parameters can be found in Appendix A10 (Substance profiles).

7.7. Transition to foragers

Procedures involved:

- AffProc (unchanged)
- WorkerIHBeesDevProc

The procedure WorkerIHBeesDevProc was changed as described in 7.6. Adult Development.

7.8. Consumption

Procedures involved:

- HoneyConsumptionProc
- PollenConsumptionProc (unchanged)

Variables from the consumption procedures governing the honey and pollen need of each cohort, as well as the energy requirement for brood thermoregulation have been made global variables, to be used in exposure calculations. The procedure *HoneyConsumptionProc* has been adapted by incorporating a switch to prevent the honey store from becoming negative in case the BEEHAVE model does not stop after the colony dies.

Furthermore, the honey storage procedure has been modified. Honey stores are represented by the amount of energy provided by the honey in kJ. Nectar collected by bees is ripened in uncapped honey stores for five days until the cells are capped (Winston 1991; Seeley 1995; Eyer et al. 2016). To simulate this process, $\text{BEEHAVE}_{\text{ecotox}}$ introduces six honey compartments: from D0 to D4 and the “capped” honey compartment. Honey store D0 contains the nectar gathered during the day, D1 the nectar gathered the day before, D2 the nectar gathered two days ago, up until honey store D4. Any nectar older than this belongs to one large store referred to as “capped”. Each store tracks the total energy and the concentration of pesticide within the store. The pesticide concentration within each store is assumed to be completely mixed-in to

represent the worst-case scenario (Rumkee et al., 2017). Implementing different honey store compartments ensures that the colony receives a conservative dose. Additionally, the bees consume the newest honey and, thus, receive a high pesticide concentration after an application, accounting for possible acute effects.

The submodel consumption is extended by in-hive exposure to pesticide. This new module involves the following procedures:

- TupdateInternalExposureNectar_ETOX
- Tfeed_on_honey_stores_cohorts_ETOX
- Tfeed_on_honey_stores_foragers_ETOX.

The procedure *TupdateInternalExposureNectar_ETOX* calculates the amount of honey and pollen consumed by each of the in-hive cohorts (in-hive adults and larvae, *Tfeed_on_honey_stores_cohorts_ETOX*) and by the forager squadrons (*Tfeed_on_honey_stores_foragers_ETOX*). Based on the consumed amount, the procedure calculates the respective oral doses for each cohort.

The procedure *TupdateInternalExposureNectar_ETOX* includes also in-hive degradation of the pesticide. If degradation of honey is set to *true* via the GUI (*ETOX_degradation_honey*), the pesticide will dissipate over time following single first order degradation with the DT_{50} set through the interface in each of the five honey compartments (D0 – 4 and capped).

$$Concentration = Concentration * e^{\left(\frac{\ln(2)}{DT_{50}}\right)} \quad \text{(Eq.7)}$$

where *DT50* is the half-life of the pesticide in honey. It is important that next to chemical dissipation, also biological dissipation contributes to a decrease of pesticide concentration in the hive. As the bees consume contaminated honey, the pesticide is removed from the hive over time, which is referred to as biological dissipation. Measurements of pesticide degradation within the hive will include both processes.

After this procedure, the compartments are moved on, with D0 becoming D1, D1 becoming D2, and finally, the D4 honey store being added to the capped honey store. For this final step, the concentration of the capped honey store is altered by:

$$Capped\ Conc. = \frac{(Capped\ Conc.\cdot\ Capped\ Volume) + (D4\ Conc.\cdot\ D4\ Volume)}{Capped\ Volume + D4\ Volume} \qquad \text{(Eq.8)}$$

The actual exposure is then calculated in *TupdateInternalExposureNectar_ETOX*. Individual cohorts assess their energy need and take it from the nectar and pollen stores. When individuals consume pollen or honey from the hive stores, their oral pesticide dose is increased by the concentration within the hive pollen or honey stores multiplied by the daily pollen or honey need.

Larvae consume a set amount of each food source every day (*DAILY_HONEY_NEED_LARVA* and *DAILY_POLLEN_NEED_LARVA*). For larvae, the pesticide doses are reduced by a pollen or honey feeding factor (*ETOX_FF_Nursebees_Pollen* and *ETOX_FF_Nursebees_Nectar*). This factor represents the filtering of the nurse bees, which have been found to deliver reduced amounts of pesticide to the larvae (Böhme et al., 2018; Davis & Shuel, 1988). The pesticide dose from the pollen or nectar are calculated as follows:

$$Dose\ from\ pollen = In\ hive\ pollen\ conc.\cdot Daily\ larval\ pollen\ need \cdot Pollen\ feeding\ factor \qquad \text{(Eq.9)}$$

Adults also consume a set amount of pollen and honey each day (*DAILY_POLLEN_NEED_ADULT* and *DAILY_HONEY_NEED_ADULT_RESTING*), additionally to the nectar need for brood care (*ETOX_EnergyThermo*). Foragers have additional flight cost energy requirements (described in detail in the Foraging model).

ETOX_EnergyThermo represents the nectar required [kJ] for care (thermoregulation) of brood: It is a function of the total brood number and the energy needed per brood cell.

$$ETOXEnergyThermo = TotalWorkerAndDroneBrood * \frac{THERMOREGULATION\_BROOD}{1000 * ENERGY\_HONEY\_per\_g} \qquad \text{(Eq.10)}$$

where *THERMOREGULATION_BROOD* is calculated as the additional nectar [mg] required per brood individual.

The procedure *Tfeed_on_honey_stores_cohorts_ETOX* is used to calculate feeding on nectar. Therefore, it also includes the oral exposure of both in-hive adults and larvae. The individual

cohorts take their required amount of honey from the D0 store and are provided with the relevant dose of pesticide (added to the cohort's $ETOX\_PPPOralDose$ variable).

$$Dose = Dose + volume\ consumed * D0\ honey\ conc. \qquad \text{(Eq.12)}$$

If there is insufficient D0 honey, the available D0 honey is consumed, and the dose received is calculated as above. Subsequently, any remaining consumption requirements are taken from the next honey store (D1). The received dose is again calculated as above using the D1 honey concentration. This pattern continues until the D4 honey stores are consumed and capped honey is used.

For larvae, the pesticide doses are calculated the same way as for adults but additionally reduced by a nectar feeding factor (*ETOX_FF_Nursebees_Nectar*). The factor represents the proportion of pesticide from the nectar taken up by the nurses during the feeding process (as for the pollen feeding).

A similar procedure occurs for the foragers in *Tfeed_on_honey_stores_foragers_ETOX*. While for the in-hive cohorts, individual level doses are the total dose for the cohort divided by the varying *number* of the cohort (how many bees are in that cohort), for the foragers the dose is divided by the constant *SQUADRON_SIZE* (by default 100).

7.9. Swarming

Procedures involved:

- SwarmingProc

This procedure has been changed to set up experimental conditions and to incorporate the ecotox-specific honey stores in parental and prime swarms.

7.10. Beekeeping

Procedures involved:

- BeekeepingProc

This procedure has been changed to include experimental conditions such as honey harvesting, feeding schedule, and merging colonies, and to incorporate the ecotox-specific honey stores in parental and prime swarms.

7.11. Graphic user interface and output.

A new procedure *Tplot_exposureandeffects_ETOX* is added to the existing list. This procedure plots ecotox-specific outputs.

II. THE FORAGING MODEL

7.II.1. Foraging

Procedures involved:

- Start_IBM_ForagingProc (unchanged)
- Foraging_ProbabilityREP (unchanged)
- Foraging_PeriodREP

The procedure *Foraging_PeriodREP* was complemented to define the daily amount of water needed in the colony for cooling (if water foraging is considered).

7.II.2. Forager development

Procedures involved:

- ForagersDevelopmentProc (unchanged)
- NewForagersProc
- ForagersLifespanProc

In the procedure *NewForagersProc,* sensitivity of the forager squadron to pesticide through the oral and contact pathways is set through assigning a random floating-point number between 0 and 1 called *ETOX_rdm_Survival_oral* and *ETOX_rdm_Survival_contact* to represent their sensitivity for oral and contact exposure.

In the procedure *ForagersLifespanProc*, exposure to the pesticide of forager squadrons for both oral and contact doses are calculated as:

$$pesticide\ exposure = 1 - \frac{1}{1 + \frac{Dose}{LD50}^{SLOPE}} \quad \text{(Eq.13)}$$

where *Dose* is the dose (oral or contact) received by the forager squadron, *LD50 (ETOX_Forager_Contact_LD50 or ETOX_Forager_Oral_LD50)* is the LD50 value for the pesticide in question, and *Slope (ETOX_Forager_Contact_SLOPE or ETOX_Forager_Oral_SLOPE)* is the slope of the dose-response relationship.

The input parameters for the LD_{50} and the slope are derived the same way as for adult bees (see 7.6. Adult development). The contact exposure parameters can be calculated based on a honeybee acute contact study (OECD, 1998b). Study data needs to be scaled to a daily exposure contact dose and a daily effect, which is straightforward since the bees are exposed only to a single dose and observed for 48 hours. For details on the derivation of the parameters see Appendix A10 (Substance profiles).

This exposure to the pesticide is calculated for each forager squadron, and if the number is higher than that squadron's sensitivity for the exposure pathway in question (contact or oral), the forager squadron dies.

7.II.3. Foraging round

Procedures involved:

- ForagingRoundProc
- FlowerPatchesUpdateProc
- Foraging_start-stopProc
- Foraging_searchingProc
- Foraging_collectNectarPollenProc
- Foraging_flightCosts_flightTimeProc
- TflightCostupdate_ETOX (new)
- Foraging_mortalityProc
- Foraging_dancingProc (unchanged)
- Foraging_unloadingProc

Water foraging procedures has been added alongside the ecotoxicological processes. The flower patches in the model have new variables introduced to enable the modelling of water foraging. These variables are the volume of water over a full day (e.g., puddles) and details surrounding water foraging (handling time, flight cost, background risk and a counter for the number of trips made each day). The variables mirror these for the foraging of pollen and nectar. Thus, the procedure *ForagingRoundProc*, calculates now all the foragers, including water foragers with three new introduced activities (Tab. 2), while the procedure *FlowerPatchesUpdateProc* has been changed to consider mortality risk because of water handling.

Within the model, water is required for two processes: dilution of honey for feeding and cooling of the hive. Capped honey has a reduced water content of 20%. However, for feeding the honey needs to be diluted to the water content of 50% (Hooper, 2010; Knopper et al., 2016). There is a limited time in the morning (default: 1 hour) in which the bees undertake water foraging (Lindauer, 1954) for dilution, while water for cooling is carried out as required.

These rationales are reflected in the procedure *Foraging_start-stopProc* to calculate the water need of the colony and to assign foragers for water search. At the beginning of each foraging round in the model, before foragers are allocated to other activities, the number of foragers required for water foraging is calculated. If water is needed for the dilution of honey, the number of required foragers is calculated first, followed by the water for cooling:

$$\text{Foragers required}=\frac{\text{Water required for dilution(g)}}{\text{Water per forager (g)}\cdot\text{Possible trips per hr}\left(\text{hr}^{-1}\right)\cdot\text{Water foraging period(hr)}} \qquad \text{(Eq.14)}$$

$$\text{Foragers required}=\frac{\text{Water required for cooling (g)}}{\text{Water per forager (g)}\cdot\text{Possible trips per hr}(\text{hr}^{-1})\cdot\text{Daily foraging period (hr)}} \qquad \text{(Eq.15)}$$

where the water per forager is based on Visscher et al. (1996) with 0.044 [ml] water per trip. During this time, the required foragers are focussing on collecting water and are not foraging for nectar or pollen, based on the observations of Lindauer (1954). Therefore, their state variable *ETOX_WF* is set to *true* and *pollenForager* is set to *false*.

The water required for cooling is currently being set via an encoded list. This list is generated via a spreadsheet outside the model and based on the observations and equations of Lindauer (1954). The water need for cooling is a function of the daily environmental temperature, hive

measurements, and material characteristics. In a first step, the heat transmission rate Q is calculated:

$$Q = k * (T_{out} - T_{in} + T_{bees}) * A \quad \text{(Eq.16)}$$

$$\frac{1}{k} = \frac{2}{\alpha} + \frac{d}{\lambda} \quad \text{(Eq.17)}$$

where k is the heat transition coefficient, based on the heat transfer coefficient α and the thermal conductivity λ (material specific); T_{out} is the environmental temperature outside the hive, T_{in} is the optimal temperature inside the hive, T_{bees} is the heat contribution of the bees, and A is the apiary surface. The heat transmission rate is then used to calculate the water amount needed in [ml/day] (G) via the water evaporation heat

$$G = \frac{Q}{r_{35}} \quad \text{(Eq.18)}$$

where r_{35} is the water evaporation heat at 35 °C. The water amount needed per day is transformed to a total volume per day by assuming a foraging period of 12 hours per day and adding a scaling factor to account for water required for increased heat transfer trough the hive entry, the buffering capacity of honey filled combs, and the delay of the heat transmission by the bees themselves (Lindauer, 1954). A detailed listing of the equations, including a template for generation the water lists for the model can be found in Appendix A8.

These values calculated for dilution of honey and cooling of the hive are summed to give the total number of water foragers required. If there are currently not sufficient experienced water foragers available to meet the demands of the hive, each forager squadron has the probability (P) of spontaneously becoming a water forager. The probability is a function of the total water foragers required in relation to the total number of foragers in the colony.

The procedures *Foraging_searchingProc* has been changed to include the additional search for water. The nearest flower patch to the colony containing enough water for a single forager squadron is found and set as the desirable patch for water foragers. For each water foraging squadron, it is checked whether they know of a water patch (*ETOX_knownWaterPatch*). If they do not, their patch of choice is defined to be the known patch. If the foraging squadron is experienced and has a known patch, then they will return to that known patch unless it is no longer contains sufficient water for their needs. In this case they will choose a patch in their vicinity with sufficient amount of water as a new *ETOX_knownWaterPatch*. This process represents the foraging squadron sharing information about most suitable foraging locations, in the same way as information regarding nectar is communicated.

Within the *Foraging_collectNectarPollenProc* procedure, in which the collection of nectar and pollen is modelled, water foraging, including collection and returning of water, has been added. Each foraging squadron checks that its known flower patch still contains sufficient water for the entire squadron to carry a water load. If it does not, water foraging trip is counted as unsuccessful, and no water is returned. If there is enough water, the water within the flower patch is reduced by the volume the squadron can carry, and the squadron takes on a load of water including potential pesticide within the water for that day. When a water forager collects water from a patch, the concentration of pesticide within the collected water (*ETOX_PPPWaterConc*) is defined to be the concentration of pesticide in the water of the flower patch (*waterconc)*. Water is then returned to the colony. If water is required for dilution of food within the hive, this need is reduced by the volume being collected by the foragers. If no water is required for dilution, then the requirement for cooling is checked and if required it is reduced by the volume collected by the foragers.

The total water brought in is increased by the relevant volume, and any pesticide within the water is added to the total dose of the pesticide in the total water store. The additional pesticide dose received by bees using that water to dilute capped honey is calculated:

$$Conc.in\ capped\ honey\ [\frac{wt.}{ml}] = \frac{\frac{1}{2} * ((Dose\ in\ capped\ honey\ [wt.]) + (Dose\ in\ returned\ water\ [wt.]))}{Volume\ of\ capped\ honey\ [ml]} \quad \text{(Eq.19)}$$

The individual dose received by foragers is then increased by the concentration within the water multiplied by 10% of their crop volume, simulating water consumption (Gary & Lorenzen, 1976).

Additionally, the procedure *Foraging_collectNectarPollenProc* also counts pollination flights to two different patches to convert into pollinating activity. The procedure *Foraging_flightCosts_flightTimeProc* was complemented to calculate the duration of water foraging trips, both successful and unsuccessful. The new procedures *TflightCostupdate_ETOX* called by *Foraging_flightCosts_flightTimeProc* calculates energy and time spent on the foraging trips for nectar, pollen, and water. The new procedure is a turtle procedure and was introduced to transfer the energetic costs for a foraging trip to a turtle (bee) variable. This was necessary to be able to implement the feeding on the different honey stores and also to allow for the implementation of more complex landscapes. The original calculations are still performed through the flower patches to keep the original BEEHAVE model unchanged and. the summary energy at the end of the day is identical for both procedures. The flight cost of a

trip is specific to the flower patch and depends on the distance from the colony and any handling times required. This energy is then subtracted from the ecotox-specific honey stores in the procedure *Tfeed_on_honey_stores_foragers_ETOX*, starting from the newly deposited stores (D0).

Depending on the pesticide mode of action, the user can choose to include immediate mortality for the foragers (*ETOX_Forager_ImmediateMortality* = *true*). Based on the pesticide dose received by foraging (through both the oral and contact pathways), *Foraging_mortalityProc* calculates the likelihood of daily mortality based on the current dose received by the foragers. If this value is greater than the forager's *ETOX_rdm_survival_contact* or *ETOX_rdm_survival_oral* the forager squadron dies (see the procedure *ForagersLifespanProc* for details).

When a nectar forager unloads its nectar load into the colony stores, the overall honey stores are increased by the amount within the crop of a single forager multiplied by the number of foragers within a squadron. The procedure *Foraging_unloadingProc* calculates the new pesticide concentration in D0 honey store based on the current concentration and the amount brought in by foragers. The concentration within the D0 honey store after nectar addition is calculated as:

$$D0\,conc. = \frac{((D0\,conc.\cdot D0\,energy) + (Nectarload\,conc.\cdot (1-uptake)\cdot squadronEnergyload))}{D0\,energy + squadronEnergyload} \qquad \text{(Eq.20)}$$

where *uptake* is the dose taken in by the foragers in transit and *squadronEnergyload* is the total amount of honey (in energy value) being unloaded by the squadron.

When nectar is returned to the colony, it is assumed to consist of 80% water. However, capped honey should only contain 20% water. Thus, the excess of water is calculated (Winston, 1991):

$$Excess = \frac{Weight\ of\ nectar\ brought\ in}{Density\ of\ honey}\cdot (0.8-0.2) \qquad \text{(Eq.21)}$$

with weight of nectar calculated as the energy value of nectar brought in. This excess is then removed from the water requirements for the dilution of honey.

Additionally, the procedure *Foraging_unloadingProc* updates pollen in-hive pesticide concentration (*ETOX_InHivePollen_Conc*):

$$IH\ Pollen\ conc. = \frac{((IH\ Pollen\ conc. * IH\ Pollen\ stores) + (collected\ pollen\ conc * collected\ pollen\ weight)}{IH\ \ Pollen\ stores + collected\ pollen\ weight} \qquad \text{(Eq.22)}$$

III. THE VARROA MODEL

This model remains unchanged in this $\text{BEEHAVE}_{\text{ecotox}}$ model version.

References:

Becher, M. A., Grimm, V., Thorbek, P., Horn, J., Kennedy, P. J., & Osborne, J. L. (2014). BEEHAVE: A systems model of honeybee colony dynamics and foraging to explore multifactorial causes of colony failure. *Journal of Applied Ecology*, *51*(2), 470–482. https://doi.org/10.1111/1365-2664.12222

Böhme, F., Bischoff, G., Zebitz, C. P. W., Rosenkranz, P., & Wallner, K. (2018). From field to food—Will pesticide-contaminated pollen diet lead to a contamination of royal jelly? *Apidologie*, *49*(1), 112–119. https://doi.org/10.1007/s13592-017-0533-3

Croft, S., Brown, M., Wilkins, S., Hart, A., & Smith, G. C. (2018). Evaluating European Food Safety Authority Protection Goals for Honeybees (Apis mellifera): What Do They Mean for Pollination?: Impact of EFSA Bee Protection Goals on Pollination. *Integrated Environmental Assessment and Management*, *14*(6), 750–758. https://doi.org/10.1002/ieam.4078

Davis, A. R., & Shuel, R. W. (1988). Distribution of 14C-labelled carbofuran and dimethoate in royal jelly, queen larvae and nurse honeybees. Apidologie, 19(1), 37–50. https://doi.org/10.1051/apido:19880103

EFSA. (2009). Risk Assessment for Birds and Mammals. *EFSA Journal, 7(12):1438,* Appendix 28. https://doi.org/10.2903/j.efsa.2009.1438

EFSA. (2013). Guidance on the risk assessment of plant protection products on bees (Apis mellifera, Bombus spp. And solitary bees). *EFSA Journal*, *11*(7). https://doi.org/10.2903/j.efsa.2013.3295

EFSA. (2015). Statement on the suitability of the BEEHAVE model for its potential use in a regulatory context and for the risk assessment of multiple stressors in honeybees at the landscape level. *EFSA Journal*, *13*(6). https://doi.org/10.2903/j.efsa.2015.4125

Eyer, M., Neumann, P., & Dietemann, V. (2016). A Look into the Cell: Honey Storage in Honey Bees, Apis mellifera. PLOS ONE, 11(8), e0161059. https://doi.org/10.1371/journal.pone.0161059

Gary, N. E., & Lorenzen, K. (1976). A Method for Collecting the Honey-Sac Contents from Honeybees. Journal of Apicultural Research, 15(2), 73–79. https://doi.org/10.1080/00218839.1976.11099838

Grimm, V., Berger, U., Bastiansen, F., Eliassen, S., Ginot, V., Giske, J., Goss-Custard, J., Grand, T., Heinz, S. K., Huse, G., Huth, A., Jepsen, J. U., Jørgensen, C., Mooij, W. M., Müller, B., Pe'er, G., Piou, C., Railsback, S. F., Robbins, A. M., … DeAngelis, D. L. (2006). A standard protocol for describing individual-based and agent-based models. *Ecological Modelling*, *198*(1–2), 115–126. https://doi.org/10.1016/j.ecolmodel.2006.04.023

Grimm, V., Berger, U., DeAngelis, D. L., Polhill, J. G., Giske, J., & Railsback, S. F. (2010). The ODD protocol: A review and first update. *Ecological Modelling*, *221*(23), 2760–2768. https://doi.org/10.1016/j.ecolmodel.2010.08.019

Grimm, V., Johnston, A. S. A., Thulke, H.-H., Forbes, V. E., & Thorbek, P. (2020). Three questions to ask before using model outputs for decision support. *Nature Communications*, *11*(1), 4959. https://doi.org/10.1038/s41467-020-17785-2

Hooper, T. (2010). *Guide to bees & honey—The worlds best selling guide to beekeeping*. Northern Bee Books.

Knopper, L. D., Dan, T., Reisig, D. D., Johnson, J. D., & Bowers, L. M. (2016). Sugar concentration in nectar: A quantitative metric of crop attractiveness for refined pollinator risk assessments. *Pest Management Science*, *72*(10), 1807–1812. https://doi.org/10.1002/ps.4321

Lindauer, M. (1954). Temperaturregulierung und Wasserhaushalt im Bienenstaat. *Zeitschrift für Vergleichende Physiologie*, *36*, 391–432.

OECD. (1998a). *Test No. 213: Honeybees, Acute Oral Toxicity Test*. OECD. https://doi.org/10.1787/9789264070165-en

OECD. (1998b). *Test No. 214: Honeybees, Acute Contact Toxicity Test*. OECD. https://doi.org/10.1787/9789264070189-en

OECD. (2009). *Guidance document on overview of residue chemistry studies*. OECD.

OECD. (2016). *GD 239 Honey Bee Larval Toxicity Test following Repeated Exposure*.

Rumkee, J. C. O., Becher, M. A., Thorbek, P., & Osborne, J. L. (2017). Modeling Effects of Honeybee Behaviors on the Distribution of Pesticide in Nectar within a Hive and Resultant in-Hive Exposure. *Environmental Science & Technology*, *51*(12), 6908–6917. https://doi.org/10.1021/acs.est.6b04206

Schmickl, T., & Crailsheim, K. (2007). HoPoMo: A model of honeybee intracolonial population dynamics and resource management. *Ecological Modelling*, *204*(1–2), 219–245. https://doi.org/10.1016/j.ecolmodel.2007.01.001

Seeley, T. D. (1995). The wisdom of the hive: The social physiology of honey bee colonies. Harvard University Press.

Visscher, P. K., Crailsheim, K., & Sherman, G. (1996). How do honey bees (Apis mellifera) fuel their water foraging flights? Journal of Insect Physiology, 42(11–12), 1089–1094. https://doi.org/10.1016/S0022-1910(96)00058-3

Wilensky, U. (1999). *NetLogo*. Center for Connected Learning and Computer-Based Modeling, Northwestern University. https://ccl.northwestern.edu/netlogo/

Winston, M. L. (1991). The biology of the honey bee (1. Harvard Univ. Press paperback ed). Harvard Univ. Press.
