# Supplementary material for: The BEEHAVEecotox Model—Integrating a Mechanistic Effect Module into the Honeybee Colony Model
Source: Environ Toxicol Chem. 2022 Oct 4;41(11):2870–82. doi: 10.1002/etc.5467 (PMC9828121; doi:10.1002/etc.5467)
Supplement: Supplementary file 2 — Supporting information. [file ETC-41-2870-s011.pdf]

## APPENDIX 2 - BEEHAVE$_{ecotox}$ partial sensitivity analysis

Sensitivity analysis was conducted following the Becher et al. (2014) approach assessing the sensitivity of the model output to changes in the newly implemented parameters. In total, 19 parameters of the BEEHAVEecotox model have been assessed towards their influence on the model output (Appendix A5). Each parameter was assigned a default value (factor 1) and then multiplied by the factors 0.001, 0.01, 0.1, 0.5, 0.9, 1, 1.1, 1.5, 2, 4, 10, 100, 1000. Exceptions were made for the following parameters:

1) The parameter allowing the immediate death of foragers due to toxic effects (*ETOX_Forager_ImmediateMortality),* the parameter switching on and off water foraging *(ETOX_Water_foraging),* and the parameter allowing to limit contact exposure to one day *(ETOX_contactexp_oneday*) as these parameters are Boolean ones.

2) The pollen and nectar-feeding factors (*ETOX_FF_Nursebees_Pollen* and *ETOX_FF_Nursebees_Nectar*) as their ranges lie between 0 and 1.

3) The slopes of the dose-response relationships (*ETOX_Forager_Oral_Slope*, *ETOX_Forager_contact_Slope* and *ETOX_Larvae_Oral_slope*) as the given set of factors would not provide meaningful results in sense of the dose-response relationships.

4) The duration of exposure (*ETOX_ExposurePeriod*) as a parameter that should be an integer. This parameter and those in p.3 were multiplied by a tailored set of factors (Appendix A5).

The number of adult bees was used to measure the sensitivity of the model output. The number of adults was measured at three points in time: on the day after the end of exposure, at the end of the semi-field study, and at the end of the year and averaged over ten replicates (after Becher et al. 2014). This was done to observe the immediate effect of the pesticide application on the bee colony, the postponed effect observed at the end of the standard semi-field study, and the state of beehives before winter. Sensitivity analysis was conducted for two compounds also used in validation: dimethoate, which mainly affects adult bees (Fig. S2.1), and fenoxycarb, which affects the larval stage of the honeybee (Fig. S2.2). *ETOX_Water_foraging wa*s set to false by default, except for the sensitivity analysis of the parameters *ETOX_WaterVolume* and *ETOX_WaterConc.*

The sensitivity analysis showed that the concentration of the pesticide in nectar and pollen (external exposure module) and the $LD_{50}$ for adult bees and larvae (effect module) had the

largest influence on the population dynamics of adult bees, showing a 10-100-fold increase or decrease in the number of adult bees.

The $DT_{50}$ of the pesticide, the parameters describing the filter effect of nurse bees for nectar and pollen, water source volume, and pesticide concentration in the water affected the model output the least (the number of adult bees changed less than 10%).

However, the sensitivity depended on the mode of action of the pesticide. In dimethoate simulations, the increase of the $LD_{50}$ parameter for adult bees in oral exposure (simulating a reduced bee sensitivity to the compound via a higher $LD_{50}$) led to the pronounced increase in the number of adult bees (1000-fold from factor 0.01 to 1). In fenoxycarb simulations, however, this parameter did not significantly affect the model output due to the default value being very large, i.e., due to its mode of action.

In general, the effects of the parameters with time after exposure preserved the direction of the change in the number of adult bees (compare blue line trends with the green line trends in Fig. S2.1 & S2.2). However, an increase of the *ETOX_RUD* (residue per unit dose in contact exposure, see Appendix A1, 7.2. Updates) caused the immediate decrease of the adult bees after the application, but a slight increase towards the end of the simulation. One of the explanations could be that the foragers received a higher contact dose (corresponding to the higher RUD) and died faster. Therefore, less pesticide was brought back to the hive and thus, reducing the long-term effects of oral exposure via the pesticide stored in the hive. Additionally, towards the end of the year, population dynamics were also influenced by other processes in the model leading to the decrease of the number of adult bees and, thus, making the influence of a particular parameter of the ecotoxicological module less pronounced.

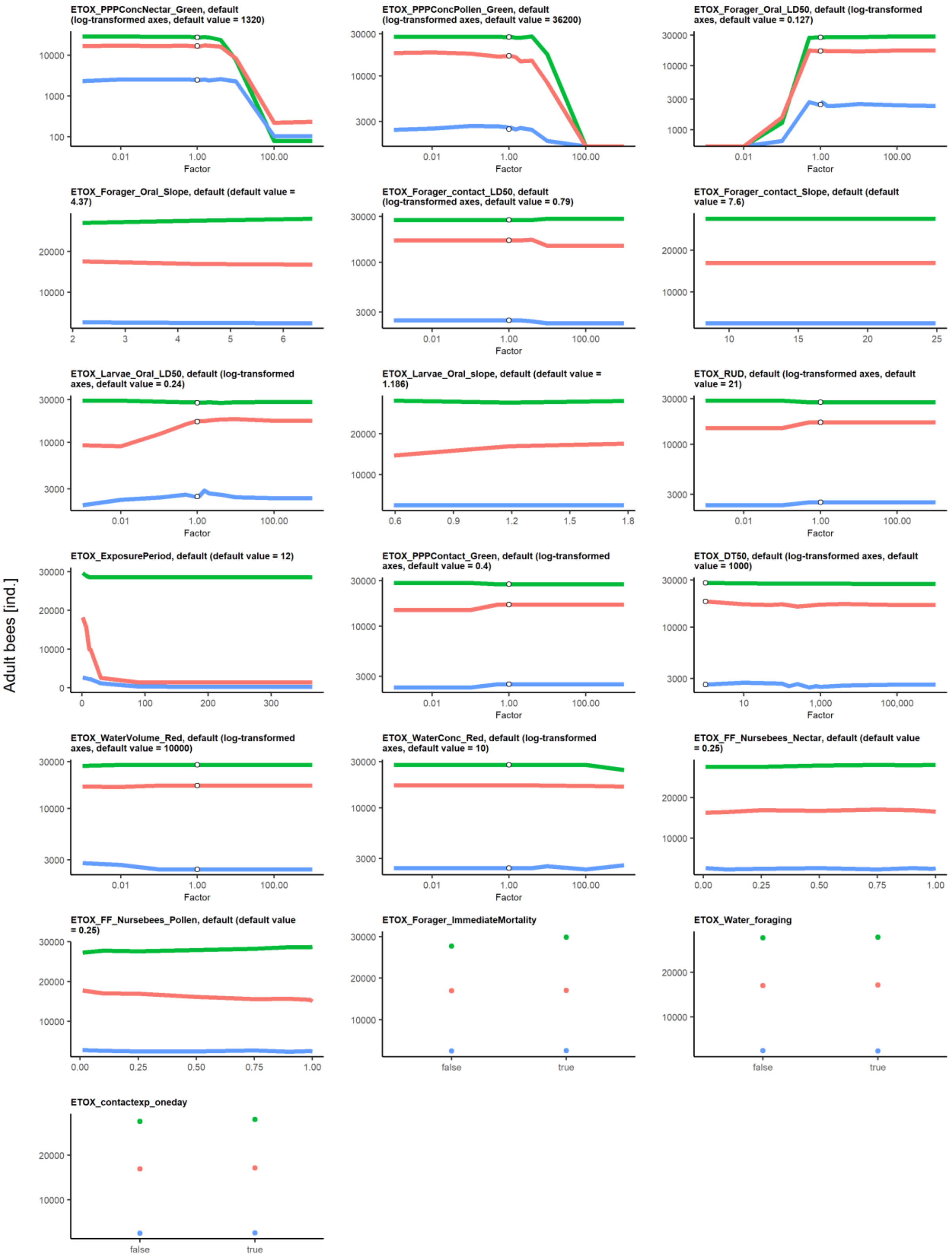


Figure S2.1. Sensitivity of the model output (number of adult bees) to the changes in the selected parameters. The default values of the parameters are from the dataset with dimethoate as toxic reference. Green lines (points): observation of the model output at the end of the exposure; red lines (points): at the end of the semi-field study; blue lines: at the end of the year.

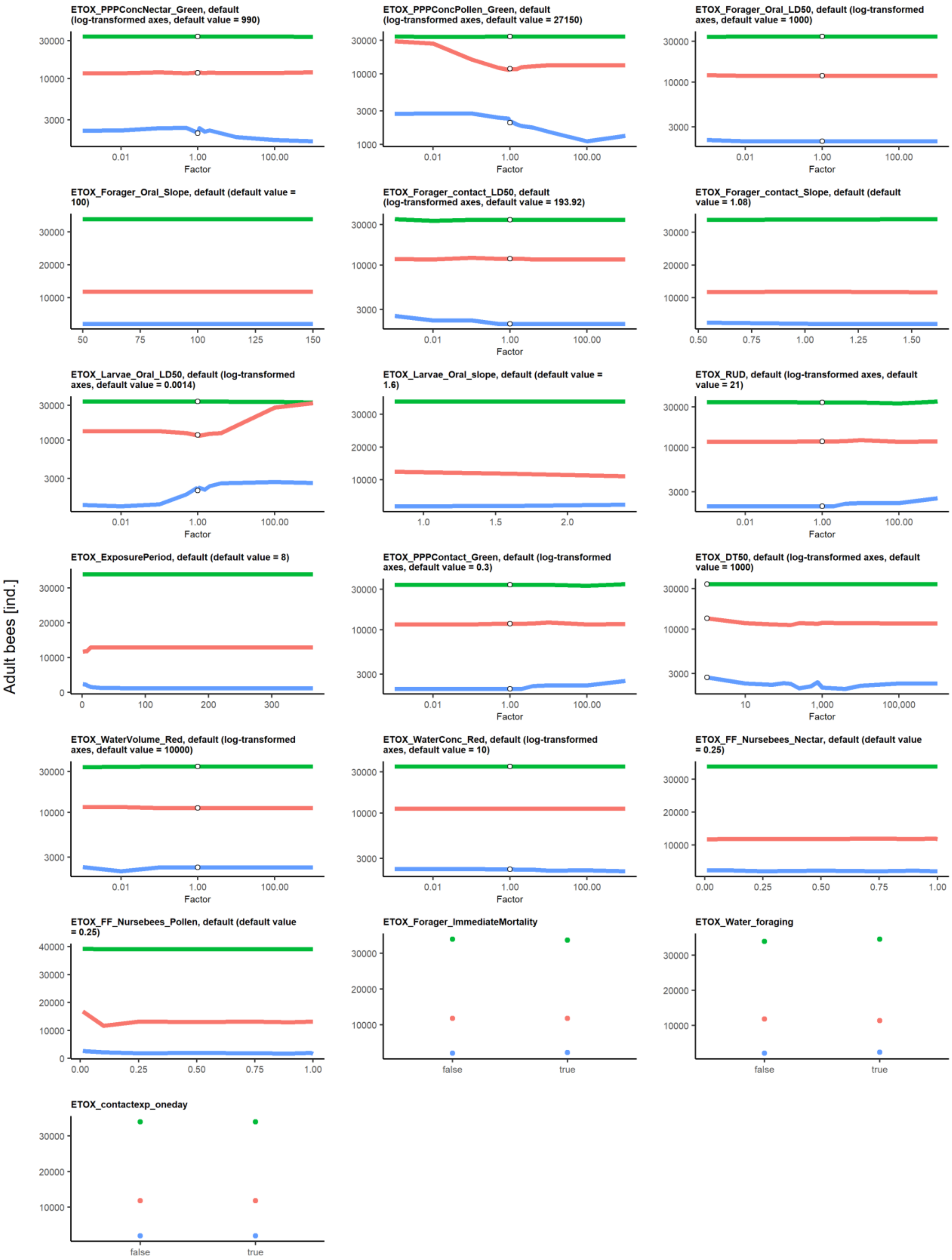


Figure S2.2. Sensitivity of the model output (number of adult bees) to the changes in the selected parameters. The default values of the parameters are from the dataset with fenoxycarb as toxic reference. Green lines (points): observation of the model output at the end of the exposure; red lines (points): at the end of the the semi-field study; blue lines: at the end of the year.

## REFERENCES

Becher, M. A., Grimm, V., Thorbek, P., Horn, J., Kennedy, P. J., & Osborne, J. L. (2014). BEEHAVE: A systems model of honeybee colony dynamics and foraging to explore multifactorial causes of colony failure. *Journal of Applied Ecology*, *51*(2), 470–482. https://doi.org/10.1111/1365-2664.12222
