# Supplementary material for: The BEEHAVEecotox Model—Integrating a Mechanistic Effect Module into the Honeybee Colony Model
Source: Environ Toxicol Chem. 2022 Oct 4;41(11):2870–82. doi: 10.1002/etc.5467 (PMC9828121; doi:10.1002/etc.5467)
Supplement: Supplementary file 3 — Supporting information. [file ETC-41-2870-s006.pdf]

APPENDIX 3 – ECOTOX MODULE VERIFICATION

The implementation of the ecotoxicological module in the BEEHAVE$_{ecotox}$ model has been thoroughly verified by two of the authors who were not involved in the initial model development and implementation. Special attention has been paid to the verification of the applied units and their consistency throughout the graphical user interface, the code, and the model outputs. The complexity of the model and the use of different sources of input information, such as beekeeping practices, laboratory, and field ecotoxicological studies, require a careful transformation of the units when including new datasets.

Furthermore, the implementation of the toxicity was verified. This included the main exposure pathways, such as contact exposure of foragers and the oral exposure of larvae. The verification was exemplarily performed with Dimethoate by comparing the mortality rates of BEEHAVE$_{ecotox}$ with spreadsheet calculations. It was shown that the model outputs correspond to the results intended by applied formulas (Figure S.3.1-3.3).

1. Contact exposure of foragers

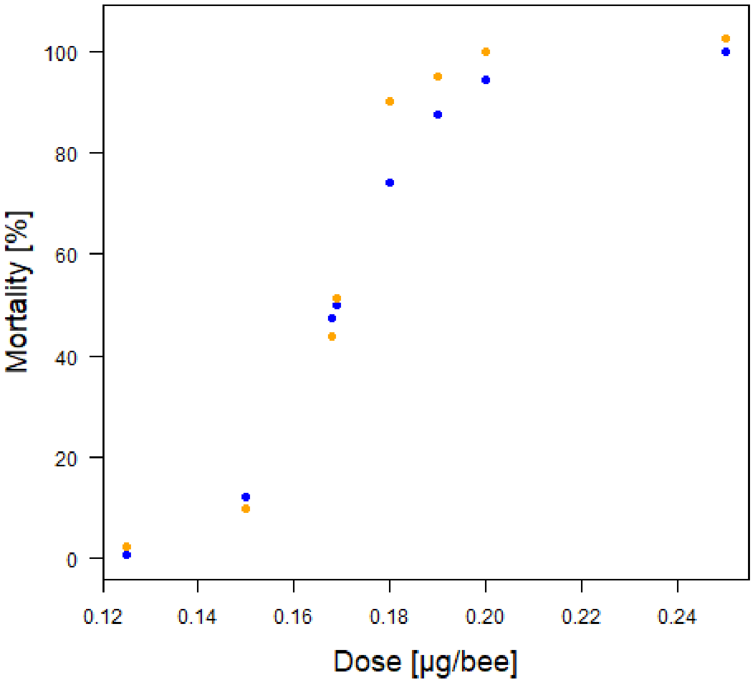

Figure S3.1. Contact forager mortality [%] in relation to dose received [µg/bee] at different concentration levels. Blue dots show the spreadsheet calculations, orange dots the BEEHAVEecotox predictions.

2. Oral exposure of larvae

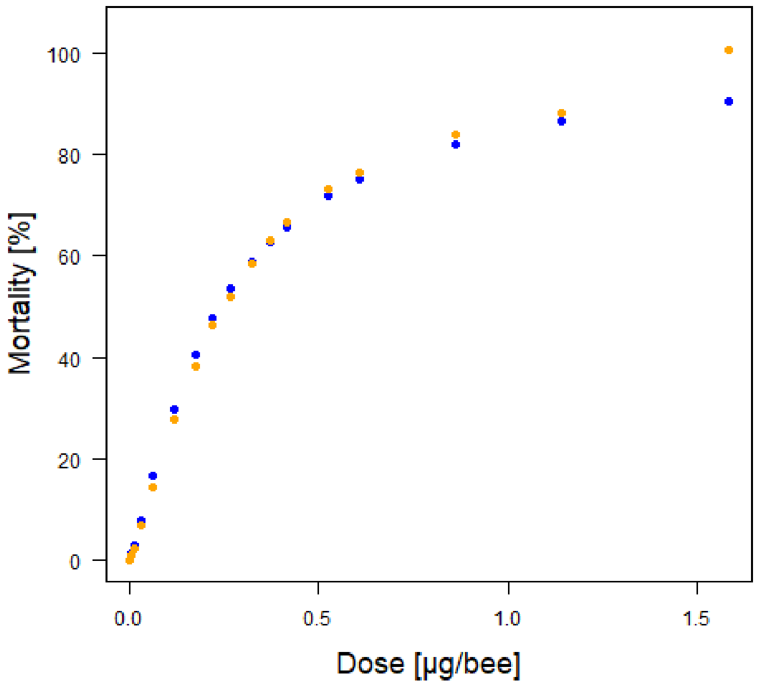

Figure S3.2. Larvae mortality [%] in relation to dose received [µg/bee] through pollen at different concentration levels. Blue dots show the spreadsheet calculations, orange dots the BEEHAVEecotox predictions.

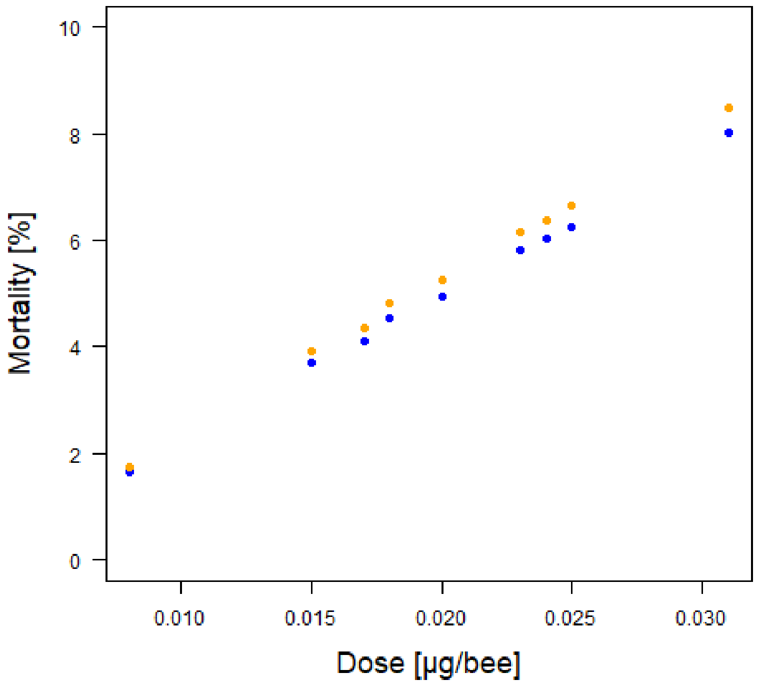

Figure S3.3. Larvae mortality [%] in relation to dose received [µg/bee] through nectar at different concentration levels. Blue dots show the spreadsheet calculations, orange dots the BEEHAVEecotox predictions.

3. Oral exposure of adult bees

The oral exposure of adult bees was not verified in this process, as it is an emergent property of the model. The individual dose obtained by an adult bee depends on the individual behavior of separate foragers and their interaction, such as the nectar need of the hive, the number of successful foragers, their energetic costs for flights, and the weather conditions. This results in a cumulative dose which is stored in the honey. Therefore, individual exposure of adult bees depends on the emerging model processes and is currently not explicitly predicted.
