# Supplementary material for: The BEEHAVEecotox Model—Integrating a Mechanistic Effect Module into the Honeybee Colony Model
Source: Environ Toxicol Chem. 2022 Oct 4;41(11):2870–82. doi: 10.1002/etc.5467 (PMC9828121; doi:10.1002/etc.5467)
Supplement: Supplementary file 4 — Supporting information. [file ETC-41-2870-s004.pdf]

APPENDIX 4 - BEEHAVE$_{ecotox}$ model validation

1.1 Validation of the ecotoxicological module

The aim of validation of the ecotoxicological module in the BEEHAVE$_{\mathbf{ecotox}}$ model as a tool in ecological risk assessment is to identify its ability to match patterns in the colony development of empirical colonies in the short-term, as well as the ability to correctly capture both initial impacts of a pesticide and behaviour of the colony during the period following the exposure.

Specifically, for the validation presented here, we intended to assess the model ability to match short-term pesticide impacts from the positive control scenarios presented in semi-field studies under tunnel conditions. The focus of this validation is the adult population response to the pesticide effect.

To validate the BEEHAVE ecotoxicological module, data from two semi-field studies have been used (Bayer, 2019; Syngenta, 2012). These studies are semi-field ecotoxicological studies selected as they use the same crop within the tunnel (*Phacelia tanacetifolia*) with pesticides applied in a single application with the use of one pesticide as the toxic reference.

Additional changes were made within the model for validation. Firstly, the egg-laying rate curve in the BEEHAVE model, taken from HoPoMo (Schmickl & Crailsheim, 2007) was altered providing a higher egg-laying rate later in the year, this was calibrated to the empirical egg data for the control scenario in dataset 1 (parameter x5 of the curve changed from 26 to 30). Secondly, the additional stress on the colony of being placed in a tunnel was introduced, represented by relocation stress with the respective increase in the adult mortality (from 0.004 to 0.0241) as it was shown in Simone-Finstrom et al. (2016). The state of the colony (number of bees plus nectar and pollen stores), size of the crop patch within the tunnel and the dates of the study were used as input values for the model with no further calibration of parameters.

For validation purposes, a weather scenario for semi-field studies was introduced allowing for 12 hours of foraging activity per day to ensure exposure to the pesticide in simulations. The model was also adjusted to switch between the patches, where one was representing the tunnel conditions, and another the monitoring site landscape settings.

1.2 Validation Methods

1.2.1 Datasets

Both datasets used are semi-field studies designed to assess the impact of a test pesticide on a honeybee colony within a tunnel. In both studies, the colonies are placed in a tunnel with a

small area of *Phacelia tanacetifolia* for a brief period and then moved to a monitoring site devoid of agricultural land or large areas of crops. The studies tested a single application of a test chemical as well as a reference pesticide (dimethoate in dataset 1 and fenoxycarb in dataset 2), as a positive control. The negative control scenarios and the reference scenarios (positive controls) have been used in this validation.

1.2.2 Values for colony parameterisation

From the data sets, for both the negative control and reference scenarios, the number of adult bees, eggs, larvae, pupae and cells containing nectar and pollen were taken (Fig. S4.1, Tab. S4.1). In the case of the nectar and pollen, these values were converted into weight using values from Schmickl and Crailsheim (2007) (0.23 g/cell pollen, 0.5 g/cell nectar).

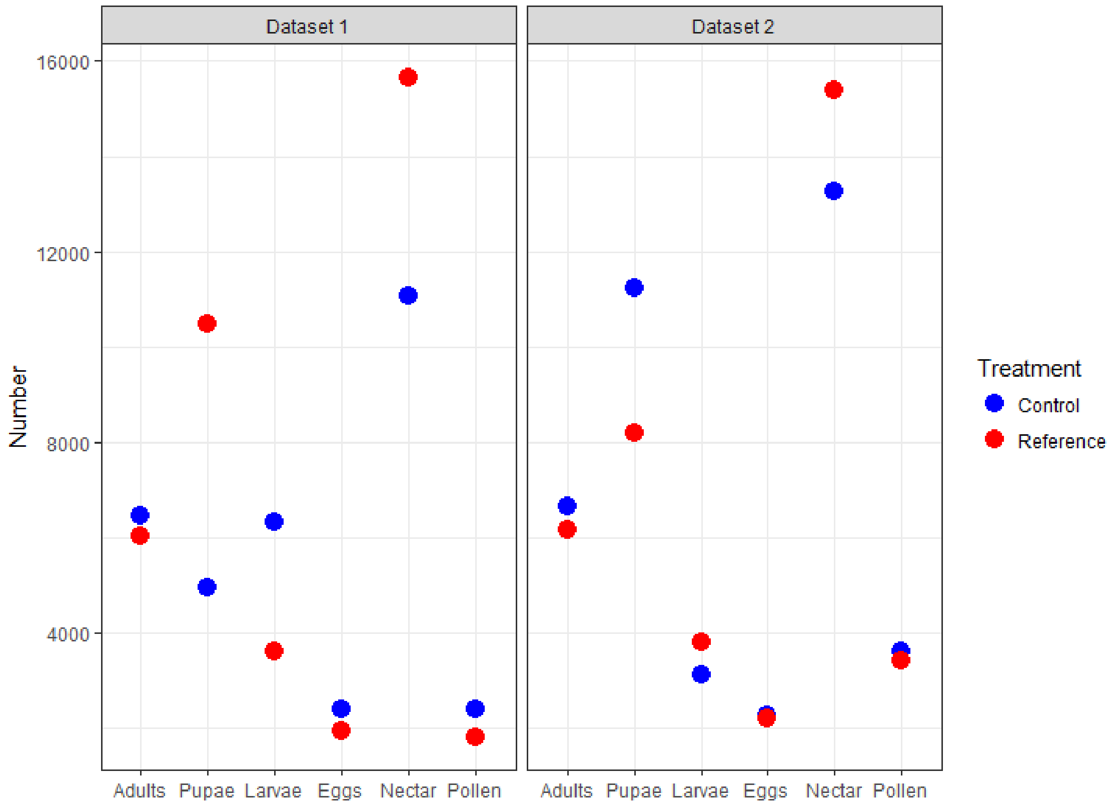

Figure S4.1. The initial values of the cohorts and food stores for both control (blue) and reference (red) scenarios in both datasets.

Table S4.1. Initial numbers of cohorts and cellfuls of both food stores for both control and reference scenarios in both datasets given as an average of three hives (Dataset 1) and four hives (Dataset 2).

	**Dataset 1**		**Dataset 2**	
	**Control**	**Reference**	**Control**	**Reference**
Adults	6,459	6,042	6,663	6,143
Pupae	4,933	10,467	11,250	8,200
Larvae	6,333	3,600	3,100	3,800
Eggs	2,400	1,933	2,250	2,200
Nectar	11,067	15,667	13,250	15,400
Pollen	2,400	1,800	3,600	3,400

1.2.3 Negative impact of the tunnel scenario

One factor not included in the ecotoxicological module of the BEEHAVE model, specific in validating the model against semi-field studies under the tunnel conditions, is the impact of confinement on the colony. This impact is required to be immediate and has a noticeable effect on the adult population in the colony without leading to long term impacts on the colony. Additional mortality of the in-hive adult bees was chosen as a proxy for the overall stress of confinement on the colony, as it meets these criteria while being a simple impact to implement and understand.

1.2.4 Crop within the tunnel

For each of the datasets, the area of *Phacelia tanacetifolia* within the tunnel was known: For dataset 1 the crop area in the tunnel is 47.52 m^2, for dataset 2: 75 m^2.

Nectar and pollen provided by BEEHAVE food patches were parameterised as the volume of nectar per patch and weight of pollen. These values were calculated per area (m^2) of *P. tanacetifolia* and then multiplied by the area within the tunnel as required for each simulation.

Data on honey and nectar availability for *P. tanacetifolia* were provided by literature as ranges (Pritsch, 2007). For honey production per hectare, the maximum value of 496 kg honey ha^{-1} was chosen (range 214-496 kg honey ha^{-1}), while a mean of 32.5% was used for sugar concentration in the nectar (range 22-43%) (Pritsch, 2007). Assuming a sugar concentration of

82% in honey, this leads to a requirement of 2.52 kg nectar to produce 1 kg of honey (Winston, 1991). Based on the maximum output of 496 kg honey ha^{-1} of *P. tanacetifolia*, this leads to a nectar availability of 0.1252 kg nectar m^{-2}. Considering *P. tanacetifolia*'s flowering period of 35 days, the nectar availability in the tunnel was set to 0.0036 kg nectar m^{-2} of crop per day.

Assuming a sugar percentage of 32.5%, the nectar has a density of 1.1391 kg/L[1] (Pritsch, 2007). Therefore, each m^2 of the crop in the tunnel provides 0.0031 L of nectar per day. As pollen weight per area or flower was not provided from the same source, the same ratio of nectar to pollen per area as in the default BEEHAVE food patches (20:1) is assumed.

1.2.5 To ensure exposure of the individuals within the colony to the pesticide, the nectar and pollen values per area within the tunnel were then multiplied by 20. Within the honeybee colony, the movement of pesticides and the mixing of nectar changes the resultant impact of a pesticide on the colony (Rumkee et al., 2017). This increase in the food availability in the crop both ensures that the colony has sufficient food within the tunnel and that pesticide exposure occurs throughout the colony, as also the realistic food levels were not sufficient to sustain the colony (see Appendix A9). This is a challenge originated in the original BEEHAVE model. The pollen budget was found to not represent realistic consumption (Schmolke et al., 2020). While in real colonies, foragers consume a negligible amount of pollen, in BEEHAVE foragers, in-hive bees, and winter bees consume the same average amount of pollen. Therefore, the forage availability was adapted. Ecotoxicological parameters

1.2.5.1 Exposure

For the pesticide concentration in the nectar and pollen of the modelled *Phacelia* crop, the maximum (residue per unit dose) values based on 1 kg/ha were taken from the EFSA guidance document (2013) (Table F1). These are 90.5 mg/kg in pollen and 3.3 mg/kg in nectar of *Phacelia*. These values are multiplied by the relevant application rate (0.4 kg/ha in dataset 1 and 0.3 kg/ha in dataset 2) to give the final values used in the model simulations.

The DT50 of the pesticide has been set to 1000 days to ensure exposure throughout the tunnel period.

[1] https://www.internetchemie.info/chemie-lexikon/daten/s/saccharose-dichtetabelle.php

The contact exposure is defined within the module by the application rate [(kg/ha)] and the contact RUD [(ha*mg)/(kg*kg)]. The application rate is 0.4 kg/ha for dataset 1 and 0.3 kg/ha for dataset 2. The RUD used for contact exposure is 21 (EFSA, 2009).

1.2.5.2 Impact of pesticide

The dose-response curves for both substances were parametrised from the study reports from contact, acute and chronic oral, and larval studies (data and details in Appendix A10). These values are given in Table 2.

Table S4.2. Ecotoxicological parameters

Pesticide	Dimethoate		Fenoxycarb	
	**LD50 (µg/bee/day)**	**slope**	**LD50 (µg/bee/day)**	**slope**
Adult – Contact	0.169	16.6	193.92	1.08
Adult – Oral	0.127	4.37	10,000*	100*
Larvae - Oral	0.24	1.186	0.0014	1.6

**No mortality seen.*

1.2.6 Weather and landscape at the monitoring site

For simplicity and to provide a conservative estimate of weather, for each of the datasets, it is assumed that there are 12 hours each day available for the colony to forage. This ensures that the foragers will be out encountering the treated crops during the tunnel period, as well as ensuring forager mortality due to foraging activity in the monitoring period.

For each empirical study, the landscape at the monitoring site was lacking agricultural crops and was significantly distant from any intensive crops that may further contaminate the colony. The food available was therefore natural wildflowers. In the validation simulations, this is assumed to be a patch providing 10% of the nectar and pollen per area of that of *Phacelia* and the colony is provided with 1 hectare of this forage but located 100 m from the colony (for the period inside the tunnel – 10 m). With such settings, the colony has the nectar flow required to maintain itself but there is not an unrealistic unlimited flow of nectar (see also Appendix A9).

1.2.7 Simulations

For each of the datasets, the model colony was set up using the data from the first day the empirical colonies were placed into the tunnel. The colony was then allowed to run until the end of the year. For each scenario run, the model was run 100 times, each with a different seed (integers from 1 to 100) from the Netlogo default random number generator. This gives each run a slightly different behaviour and ensures that any pattern seen is not solely due to the random behaviour within the BEEHAVE model.

1.3 Validation Results

Data set 1 (dimethoate as the toxic reference)

Overall, the simulations with dimethoate as the toxic reference predicted well the effects on the colony strength shown relative to the control treatment with the same starting conditions for colony structure and in-hive food store (Fig. S4.1.1.a).

The simulated number of eggs, larvae and pupae over time was, in general, lower than observed in the empirical experiment for both control and toxic reference (Fig. S4.1.2a, 3a, 4a). The simulations started with the calculations of the excess of the brood followed by the elimination of most of the eggs and some larvae. The extent of the excess depended on the starting conditions of a hive. This initial decrease in the cohorts led to the overall lower numbers in the simulations. However, the effect of dimethoate on these cohorts shown relative to the control dynamics captured the pattern of empirical observations well (Fig. S4.1.2b, 3b, 4b). For eggs, the model underpredicted the effect, while for pupae slightly overpredicted. The model simulations with dimethoate captured the dynamics of adult bees well enough. For both the control treatment and the treatment with the toxic reference, the model only overpredicted the number of adult bees inside the tunnel (Fig. S4.1.5.a). Although the toxic reference caused a short-time decrease in the number immediately after the day of the application, this decrease was later compensated by new adults developed from unaffected by dimethoate pupae. However, the accumulation of the dimethoate in nectar in the beehive caused a delayed decrease in the number of adult bees matching the empirical observations (Fig. S4.1.5.a). The dynamics of the absolute numbers of bees shown in the simulations differed from hive to hive (Fig. S4.2). Thus, the dynamics is largely driven by starting conditions of a particular hive, such as the proportion of different bee cohorts and stored nectar and pollen.

The model simulation was able to capture the dynamics in the amount of honey and nectar in hives compared to empirical observations (Fig. S4.1.6a, 7a). However, the effect of dimethoate on nectar and pollen was not pronounced in the semi-field study observations and was consequently not met by the model simulations (Fig. S4.1.6b, 7b).

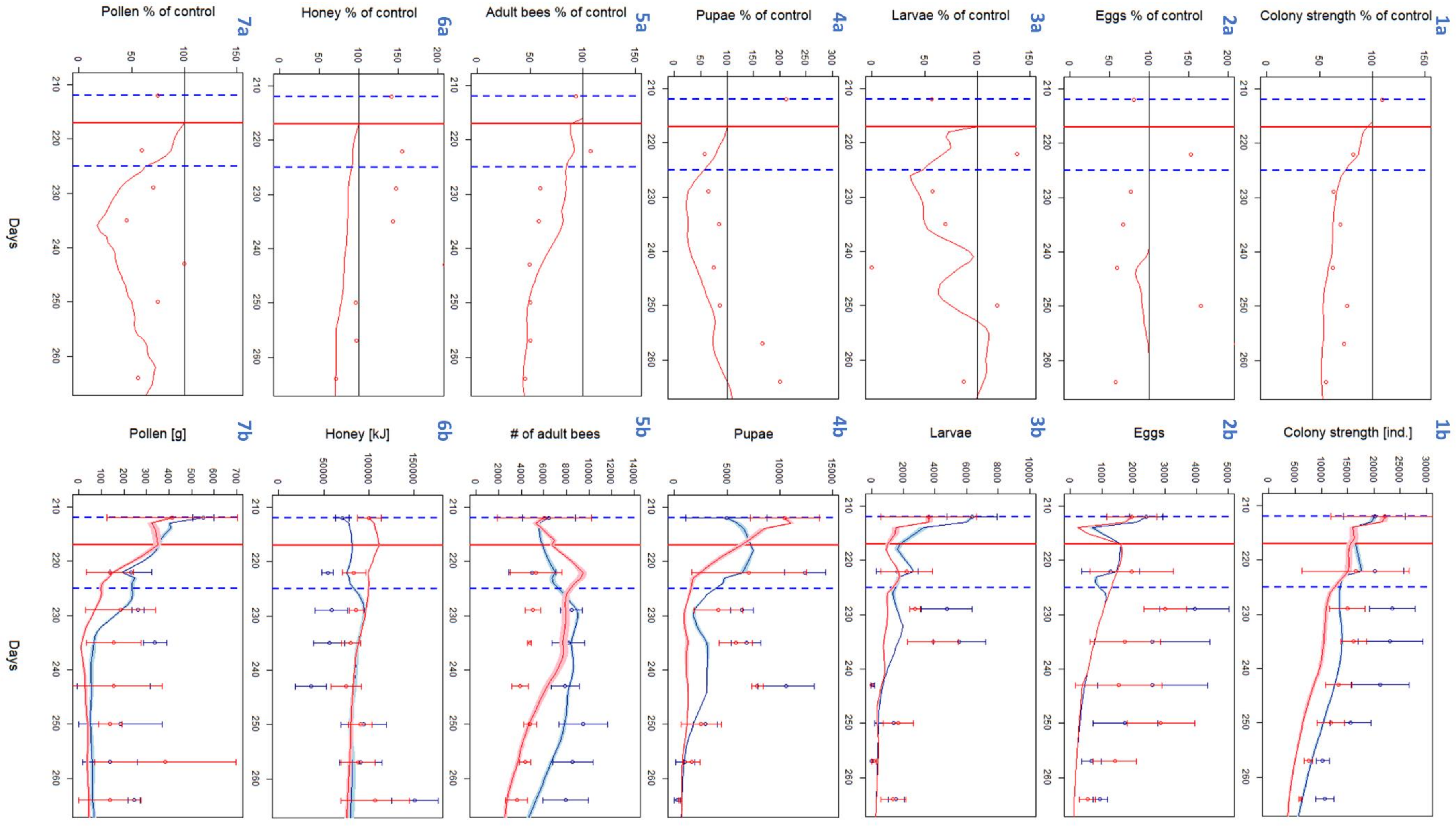

Figure S4.1. All bee cohorts (1), eggs (2), larvae (3), pupae (4), adult bees (5), honey (6), pollen (7). The dashed vertical blue lines are delineating the start and the end of the tunnel period. The vertical red line is the day of the application of the toxic reference: (a) Percentage in the experiments with the toxic reference (dimethoate) relative to the control experiments (100%-line). The red points show percentage for the semi-field study, and the red line shows percentage for the simulation experiment. Simulation experiments are run with the same starting conditions for bee cohorts and in-hive stores. (b) Number / amount over the time in the control treatment (blue) and with the toxic reference (red) observed in the semi-field study (points) and model simulation (lines with 95% confidence intervals) as an average of three beehives.

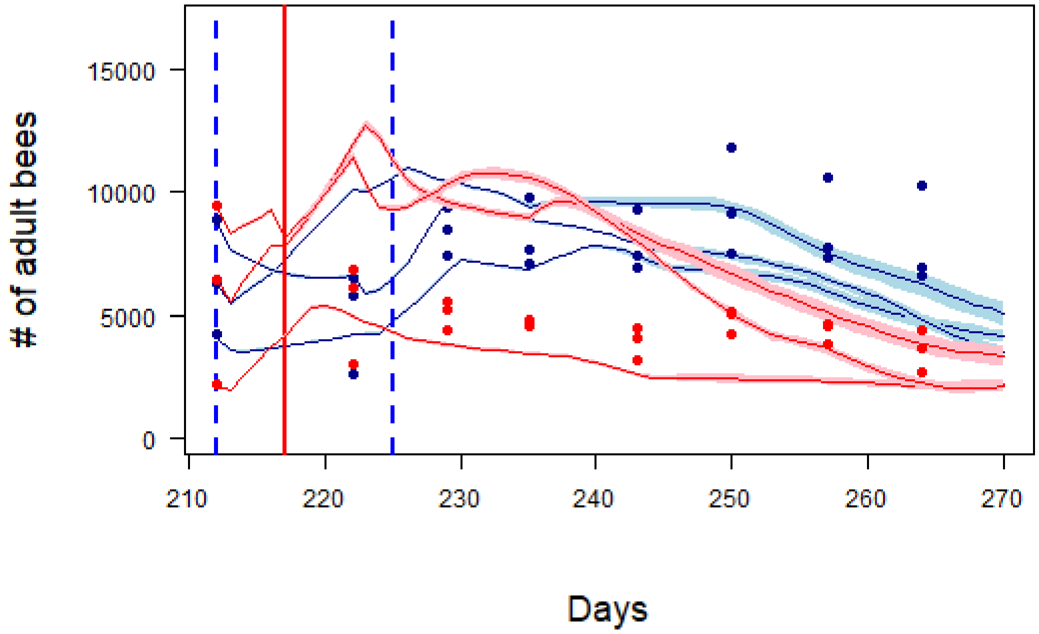

Figure S4.2. Number of adult bees over the time in the control treatment (blue) and with the toxic reference (red) observed in the semi-field study (points) and model simulation (lines with 95% confidence intervals). The number of adults is given for six beehives (three for each treatment) with different starting conditions. The dashed vertical blue lines are delineating the start and the end of the tunnel period. The vertical red line is the day of the application of the toxic reference.

Data set 2 (fenoxycarb as the toxic reference)

For fenoxycarb as the toxic reference, the simulations predicted the toxic effect on the colony strength well (Fig. S4.3.1a).

The simulated numbers of eggs, larvae and pupae were lower than observed in the empirical experiment (Fig. S4.3.2a, 3a, 4a) as a result of the elimination of a large portion of the brood at the beginning of the experiment. This phenomenon was even stronger than in the case of dimethoate and could be explained by the influence of the starting conditions, such as numbers of different cohorts, predominantly the proportion of the numbers of the brood cohorts relative to the number of nursing adult bees. However, the toxic effect of fenoxycarb on these cohorts was captured well as shown relative to control dynamics for the hives with the same starting conditions (Fig. S4.3.2b, 3b, 4b). For eggs, the simulations underpredicted the effect, and for pupae, the effect was overpredicted. For both larvae and pupae known to be particularly affected by fenoxycarb, the model was able to simulate the toxic effect.

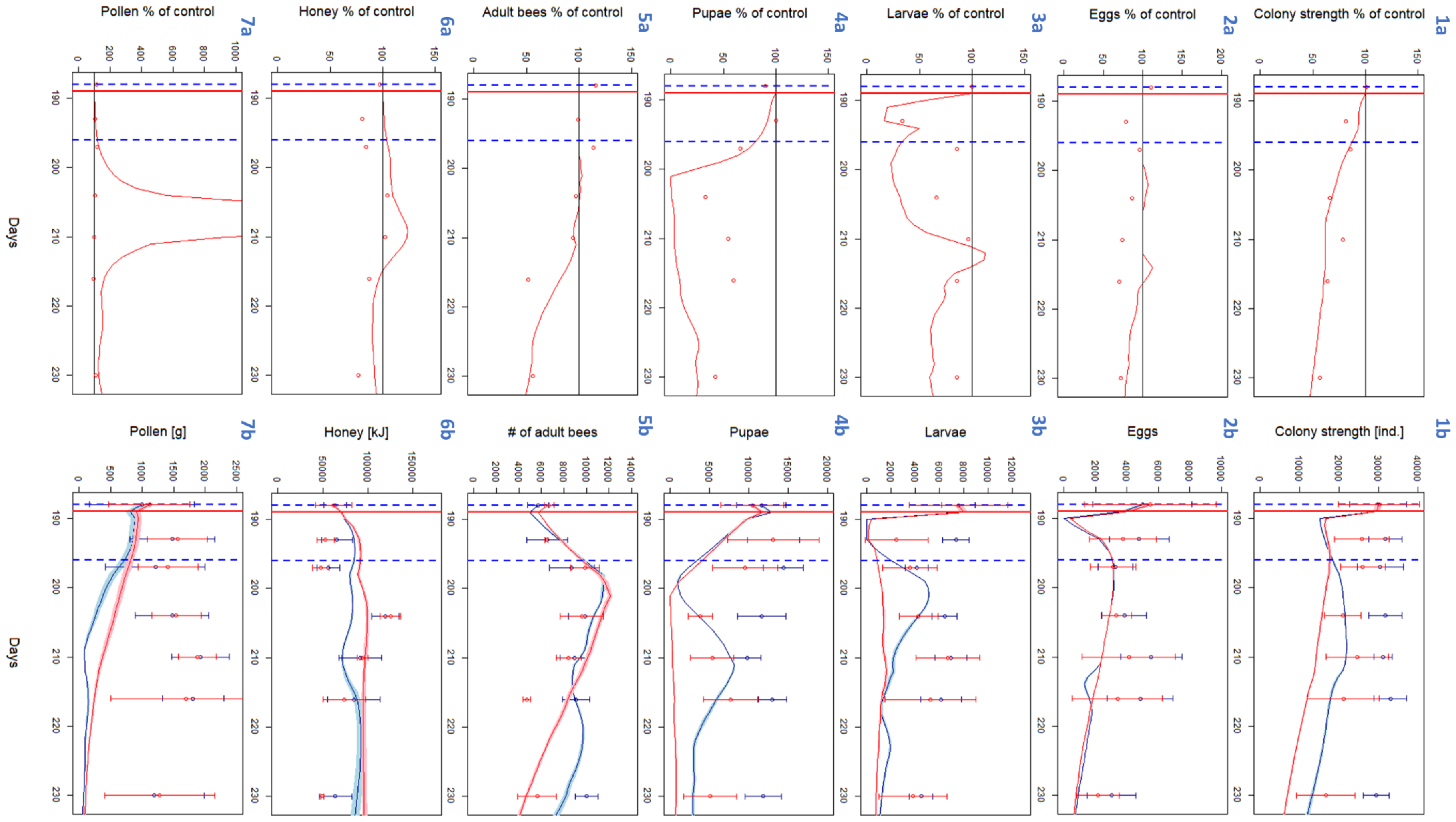

Figure S4.3. All bee cohorts (1), eggs (2), larvae (3), pupae (4), adult bees (5), honey (6), pollen (7). The dashed vertical blue lines are delineating the start and the end of the tunnel period. The vertical red line is the day of the application of the toxic reference: (a) Percentage in the experiments with the toxic reference (fenoxycarb) relative to the control experiments (100%-line). The red points show the percentage for the semi-field study, and the red line shows the percentage for the simulation experiment. Simulation experiments are run with the same starting conditions for bee cohorts and in-hive stores. (b) Number / amount over the time in the control treatment (blue) and with the toxic reference (red) observed in the semi-field study (points) and model simulation (lines with 95% confidence intervals) as an average

The model simulations were able to capture the dynamics of adult bees very well, both for control and for toxic reference (Fig. S4.3.5a). The minor overprediction of adult bee number was observed 20 days after the relocation from the tunnel (Fig. S4.3.5b), also observed in the individual hive simulations (Fig. S4.4)

The model underpredicted the amount of pollen in the hives relative to the observed in the semi-field study (Fig. S4.3.6a, 7a). For both honey and pollen, the model overpredicted their amount in the experiment with the toxic reference relative to the control experiment (Fig. S4.3.6b, S4.3.7b). However, the toxic effect on honey and pollen also was not shown as noticeable in the empirical semi-field study.

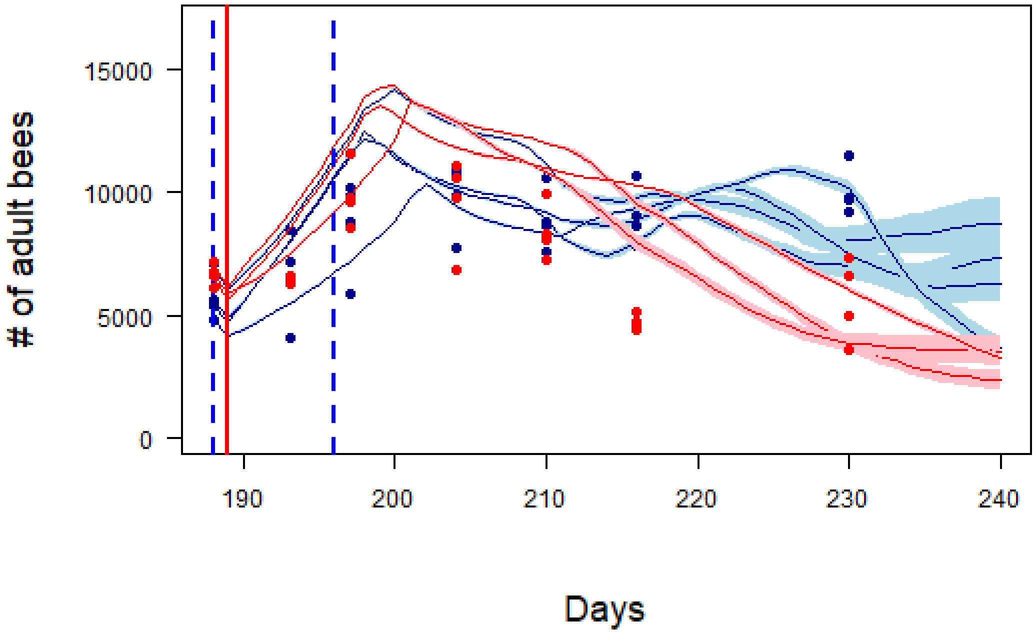

Figure S4.4. Number of adult bees over the time in the control treatment (blue) and with the toxic reference (red) observed in the semi-field study (points) and the model simulation (lines with 95% confidence intervals). The number of adults is given for eight beehives (four for each treatment) with different starting conditions. The dashed vertical blue lines are delineating the start and the end of the tunnel period. The vertical red line is the day of the application of the toxic reference.

References:

Bayer. (2019). *Iprovalicarb WG 50: Effects on Honey Bee Brood (Apis mellifera L.) under Semi-Field Conditions* (M-648855-01–1; Date: 2019-01–17). The full report can be requested by sending an email to cropscience-transparency@bayer.com

EFSA. (2009). Risk Assessment for Birds and Mammals. *EFSA Journal, 7(12):1438,* Appendix 28. https://doi.org/10.2903/j.efsa.2009.1438

EFSA. (2013). Guidance on the risk assessment of plant protection products on bees (Apis mellifera, Bombus spp. And solitary bees). *EFSA Journal*, *11*(7). https://doi.org/10.2903/j.efsa.2013.3295

OECD. (2016). GD 239 Honey Bee Larval Toxicity Test following Repeated Exposure.

Pritsch, G. (2007). Bienenweide: 200 Trachtpflanzen erkennen und bewerten. Kosmos.

Rumkee, J. C. O., Becher, M. A., Thorbek, P., & Osborne, J. L. (2017). Modeling Effects of Honeybee Behaviors on the Distribution of Pesticide in Nectar within a Hive and Resultant in-Hive Exposure. *Environmental Science & Technology*, *51*(12), 6908–6917. https://doi.org/10.1021/acs.est.6b04206

Schmickl, T., & Crailsheim, K. (2007). HoPoMo: A model of honeybee intracolonial population dynamics and resource management. *Ecological Modelling*, *204*(1–2), 219–245. https://doi.org/10.1016/j.ecolmodel.2007.01.001

Schmitzer, S., & Kling, A. (2014). Final Report – Summary of the Results of the International Ring Test for the Standardisation of a 10 Day Chronic Feeding Test on Honey Bees (Apis mellifera L.) in the Laboratory (S. 50) [OECD Report].

Schmolke, A., Abi-Akar, F., Roy, C., Galic, N., & Hinarejos, S. (2020). Simulating Honey Bee Large-Scale Colony Feeding Studies Using the BEEHAVE Model—Part I: Model Validation. Environmental Toxicology and Chemistry, 39(11), 2269–2285. https://doi.org/10.1002/etc.4839

Simone-Finstrom, M., Li-Byarlay, H., Huang, M. H., Strand, M. K., Rueppell, O., & Tarpy, D. R. (2016). Migratory management and environmental conditions affect lifespan and oxidative stress in honey bees. Scientific Reports, 6(1), 32023. https://doi.org/10.1038/srep32023

Syngenta. (2012). Semi-field study with honey bees. The full report can be requested by sending an email to Jack.Rumkee@syngenta.com.

Winston, M. L. (1991). *The biology of the honey bee* (1. Harvard Univ. Press paperback ed). Harvard Univ. Press.
