# Supplementary material for: The BEEHAVEecotox Model—Integrating a Mechanistic Effect Module into the Honeybee Colony Model
Source: Environ Toxicol Chem. 2022 Oct 4;41(11):2870–82. doi: 10.1002/etc.5467 (PMC9828121; doi:10.1002/etc.5467)
Supplement: Supplementary file 9 — Supporting information. [file ETC-41-2870-s009.pdf]

Appendix A9 – Review simulations

Table of Contents

Appendix A9 – Review simulations 1
General information 2
1. Dimethoate 3
1.1 Dimethoate: Different age structure 4
1.2 Dimethoate: Distance to monitoring site 7
1.3 Dimethoate: Nectar and pollen availability 11
1.4 Dimethoate: In-hive/forager ratio 15
2. Fenoxycarb 19
2.1 Fenoxycarb: Different age structure 20
2.2 Fenoxycarb: Distance to monitoring site 23
2.3 Fenoxycarb: Nectar and pollen availability 27
2.4 Fenoxycarb: In-hive/forager ratio 31

General information

These additional model runs were conducted to address four points raised by the reviewers:

1. **Different age structure**
   Runs compare the default model settings (one older adult bee group) versus three young adult bee groups

2. **Distance to monitoring site**
   The distance between the hive and the monitoring site was tested with three distances: 100 m (default), 10 m, and 1,000 m.

3. **Nectar and Pollen availability**
   The pollen and nectar availability was tested with three assumptions:
   a) initial settings (default) with calculated values multiplied by 20
   b) the calculated nectar and pollen availability based on the provided spreadsheet (A6)
   c) 100x the nectar and pollen availability of the default settings.

4. **In-hive / forager ratio**
   The in-hive/forager ratio was modified to 1:1 (default), 1:3, and 3:1.

1. Dimethoate

Table 1 Effects relative to control on the last day of observation for dimethoate (day 264). The empirical values are originated from the semi-field studies, the default values are the values of the BEEHAVE$_{ecotox}$ model in its default settings. Values are averages of 10 runs.

[%]	Colony strength	Adult bees	Eggs	Larvae	Pupae	Honey	Pollen
Empirical	56.18	45.27	57.13	86.95	199.6	70.6	56.23
Default	***52.04***	***43.4***	***100.12***	***105.84***	***100.12***	***70.37***	***70.16***
1. Different age structure							
**3 young bee groups**	57.88	46.42	99.19	90.4	90.88	57.71	50.47
2. Distance to monitoring site							
**10 m**	53.17	44.2	100.07	104.42	101.03	82.12	65.76
**1000 m**	50.66	42.55	100.16	104.7	95.42	65.59	64.34
3. Nectar and pollen availability							
**Calculated**	197.21	167.43	141.85	254.28	315.25	167.77	281.24
**Default * 100**	78.87	76.36	100.07	99.28	93.89	79.62	96.11
4. In-hive/forager ratio							
**1:3**	53.22	45.64	99.97	106.76	90.71	87.16	53.11
**3:1**	33.68	25.67	99.72	96.07	75.75	46.64	47.34

1.1 Dimethoate: Different age structure

default

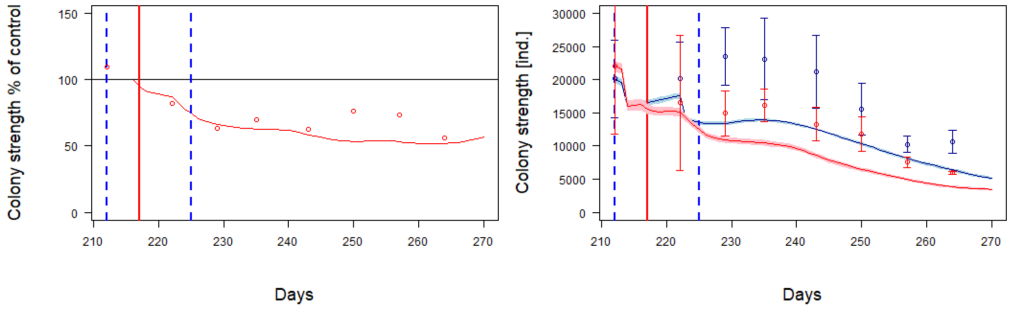

with 3 young bee groups at initialization

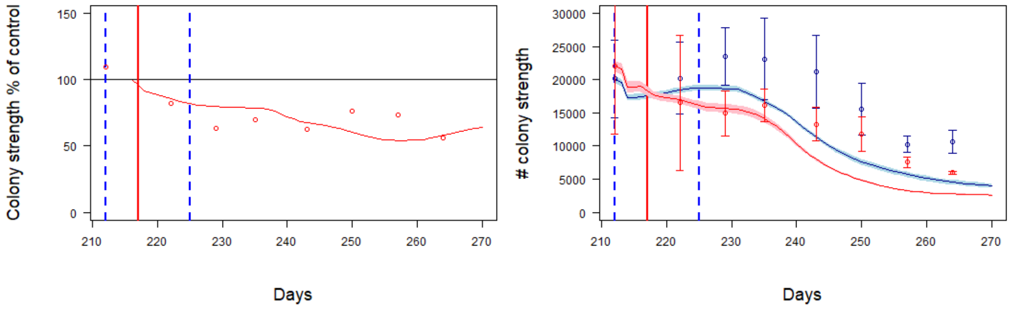

default

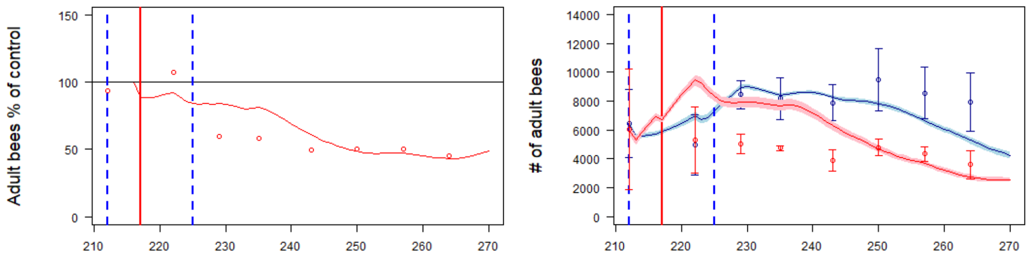

with 3 young bee groups at initialization

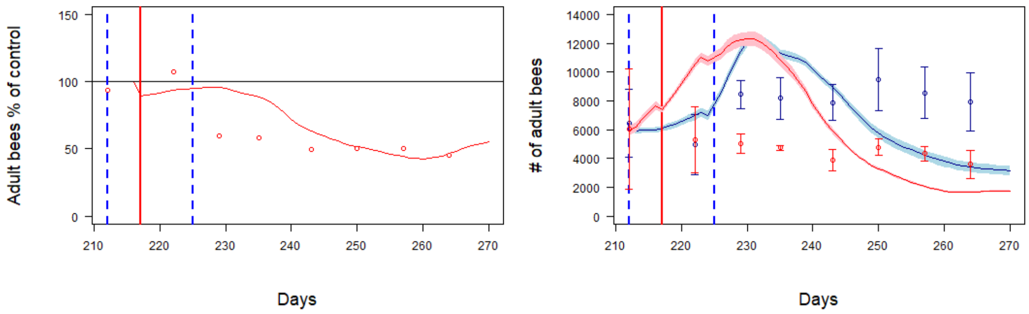

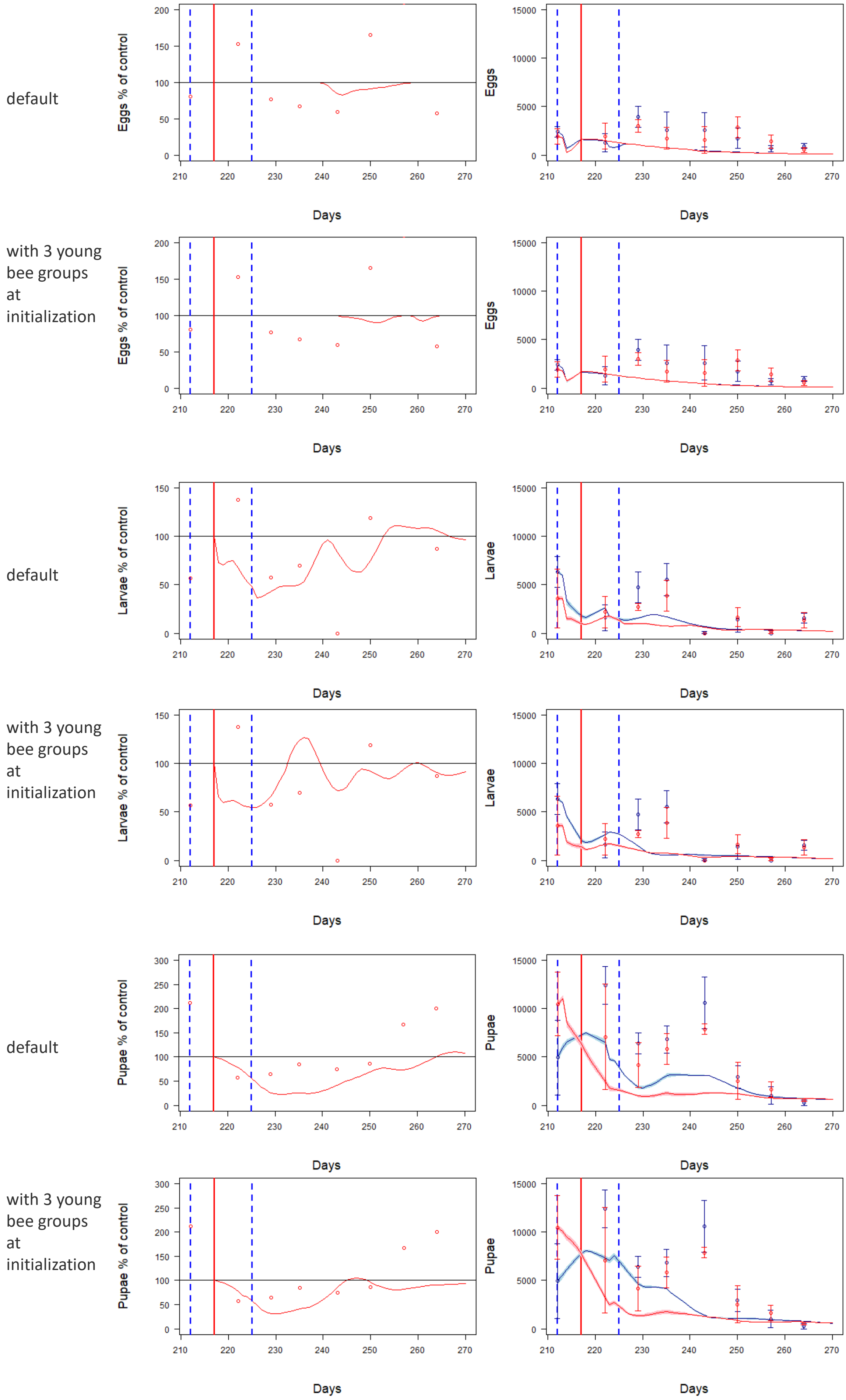
default
with 3 young bee groups at initialization
default
with 3 young bee groups at initialization
default
with 3 young bee groups at initialization
Eggs % of control
Eggs
Larvae % of control
Larvae
Pupae % of control
Pupae
Days

default

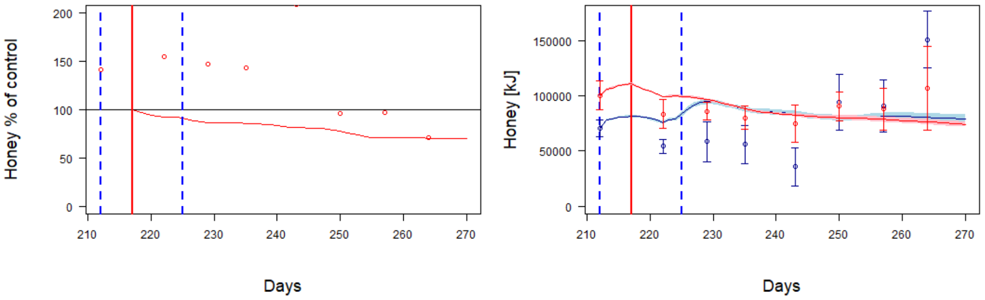

with 3 young bee groups at initialization

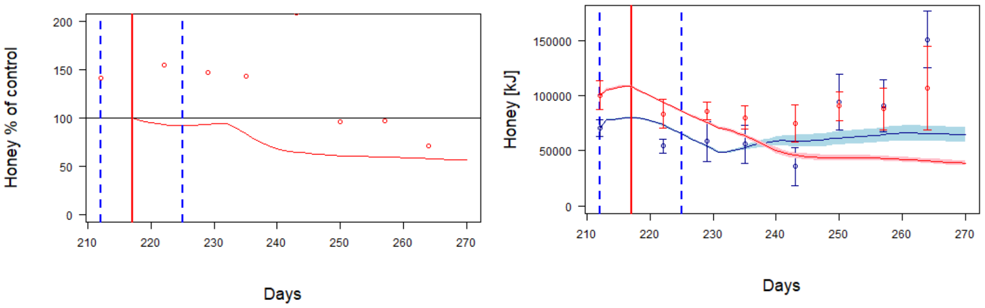

default

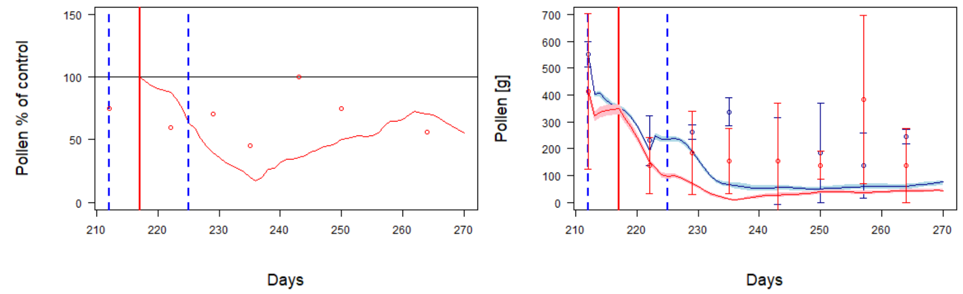

with 3 young bee groups at initialization

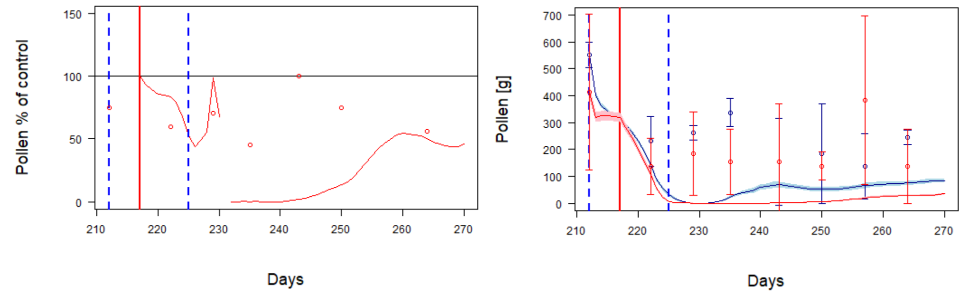

1.2 Dimethoate: Distance to monitoring site

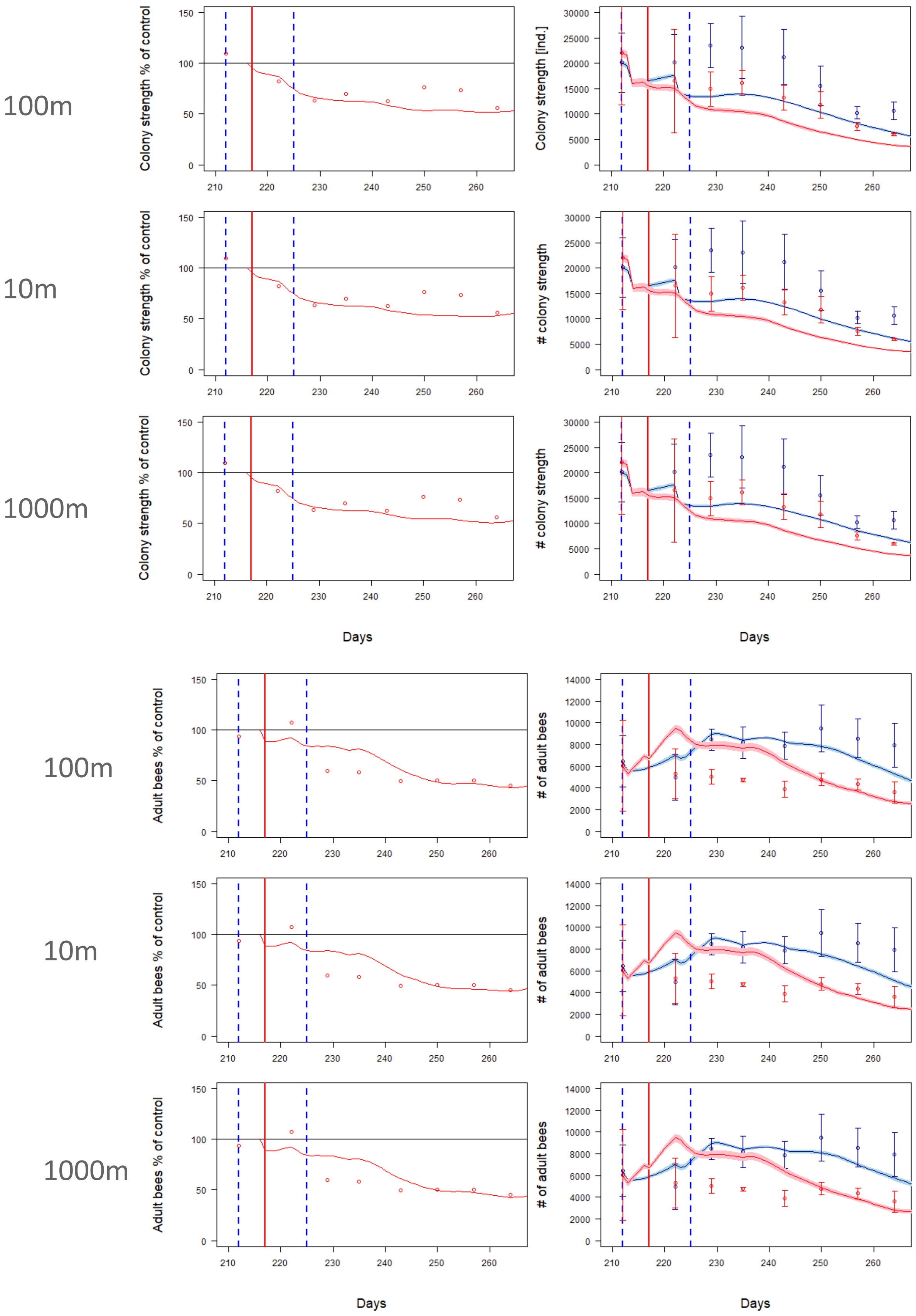

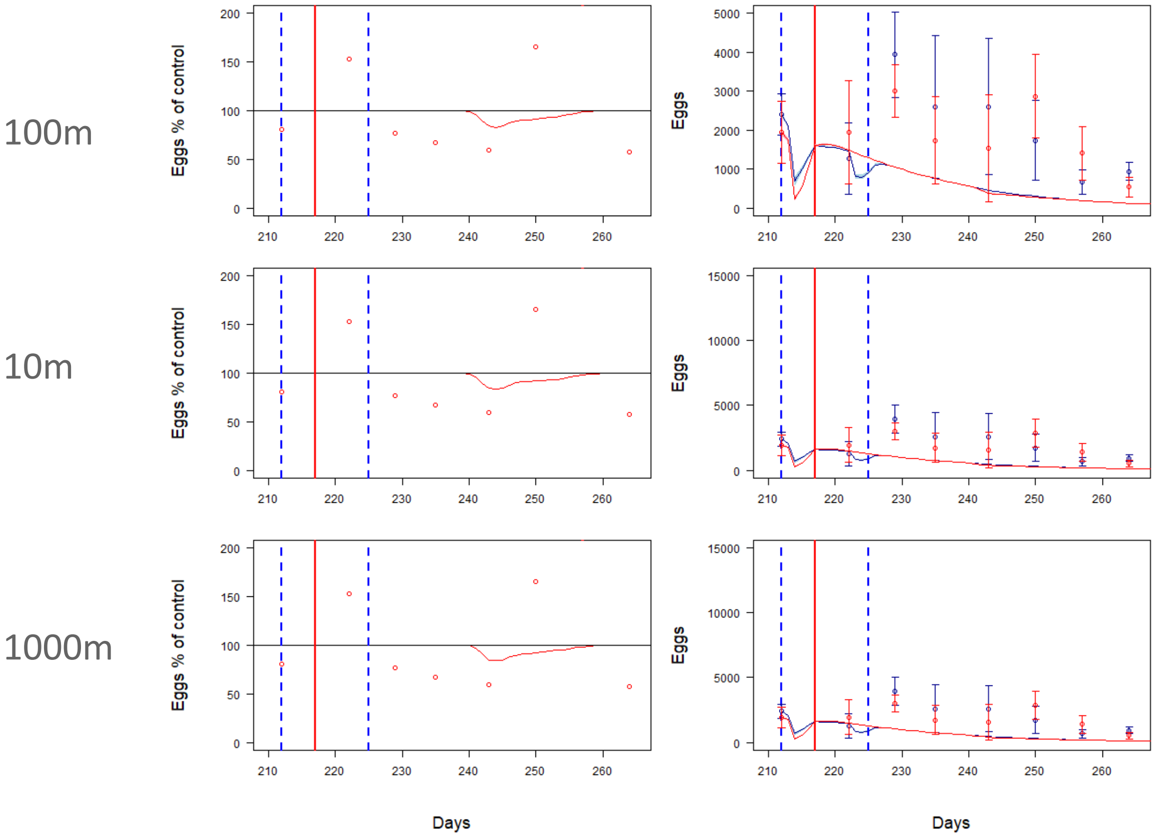
100m
10m
1000m
Eggs % of control
Eggs
Days
Days

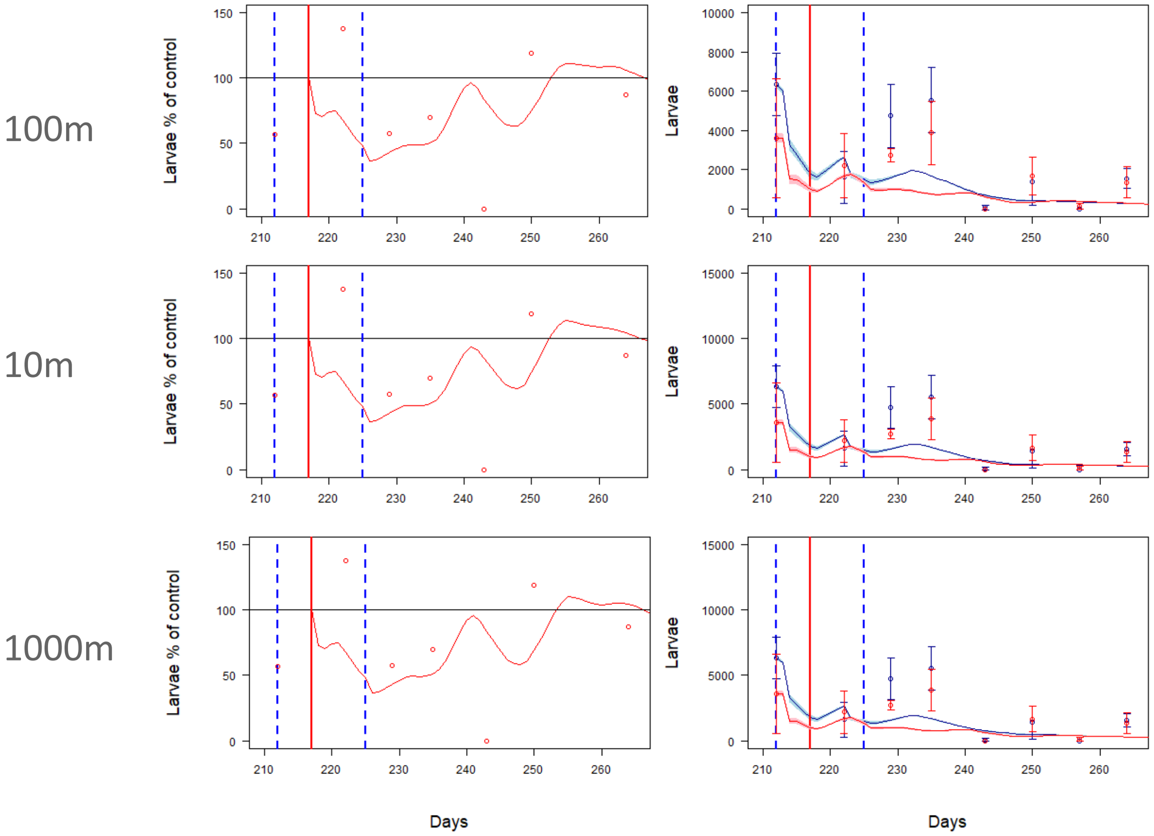
100m
10m
1000m
Larvae % of control
Larvae
Days
Days

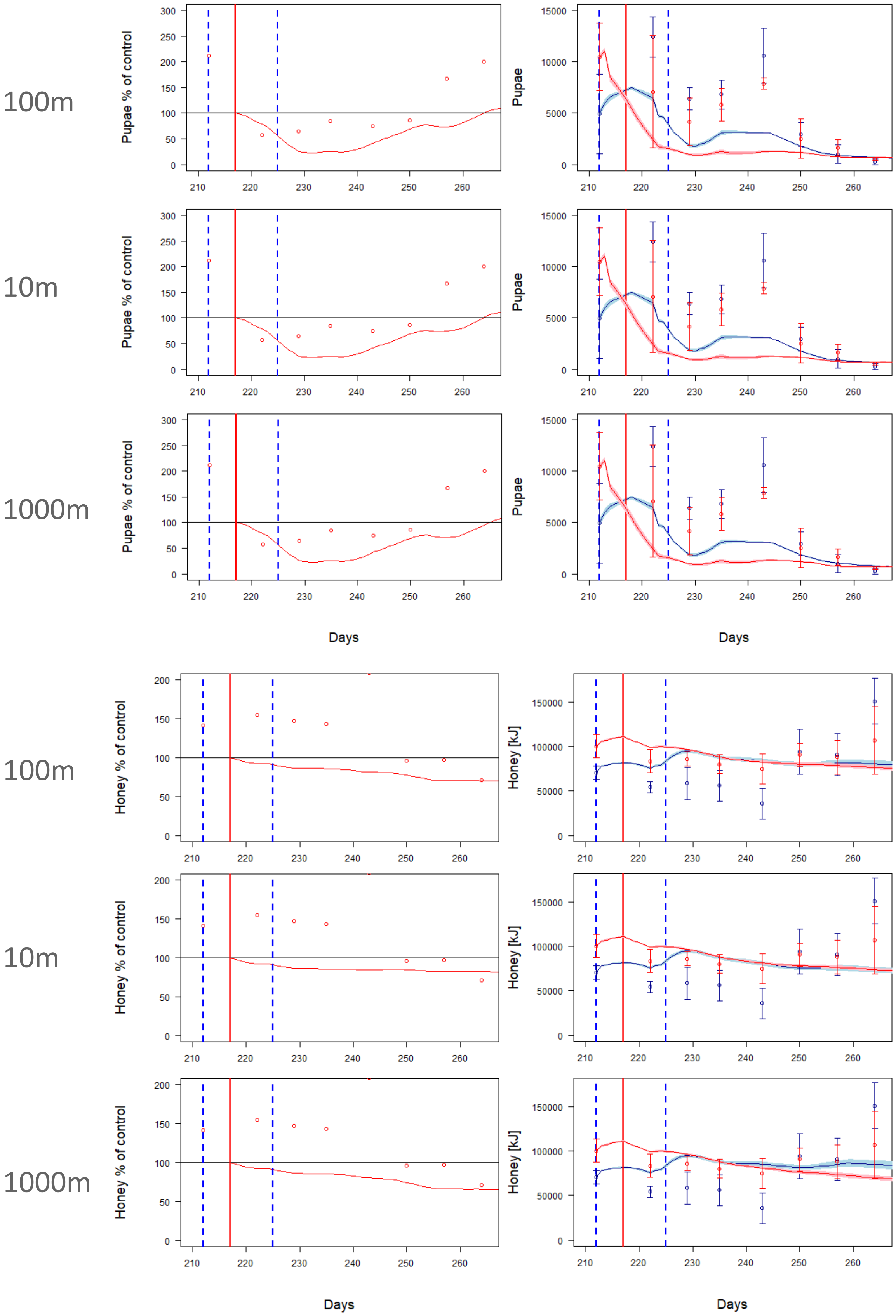
100m
10m
1000m
100m
10m
1000m
Pupae % of control
Pupae
Honey % of control
Honey [kJ]
Days

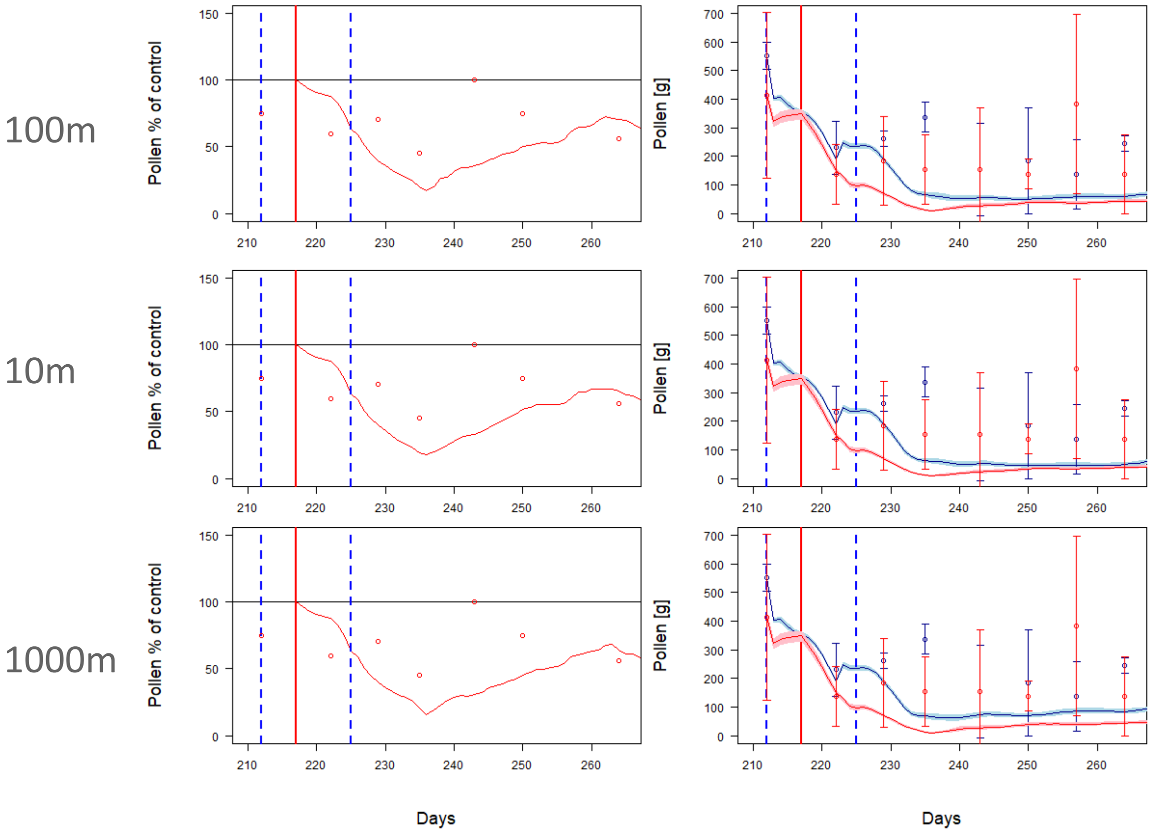

100m
10m
1000m
Pollen % of control
Pollen [g]
Days

1.3 Dimethoate: Nectar and pollen availability

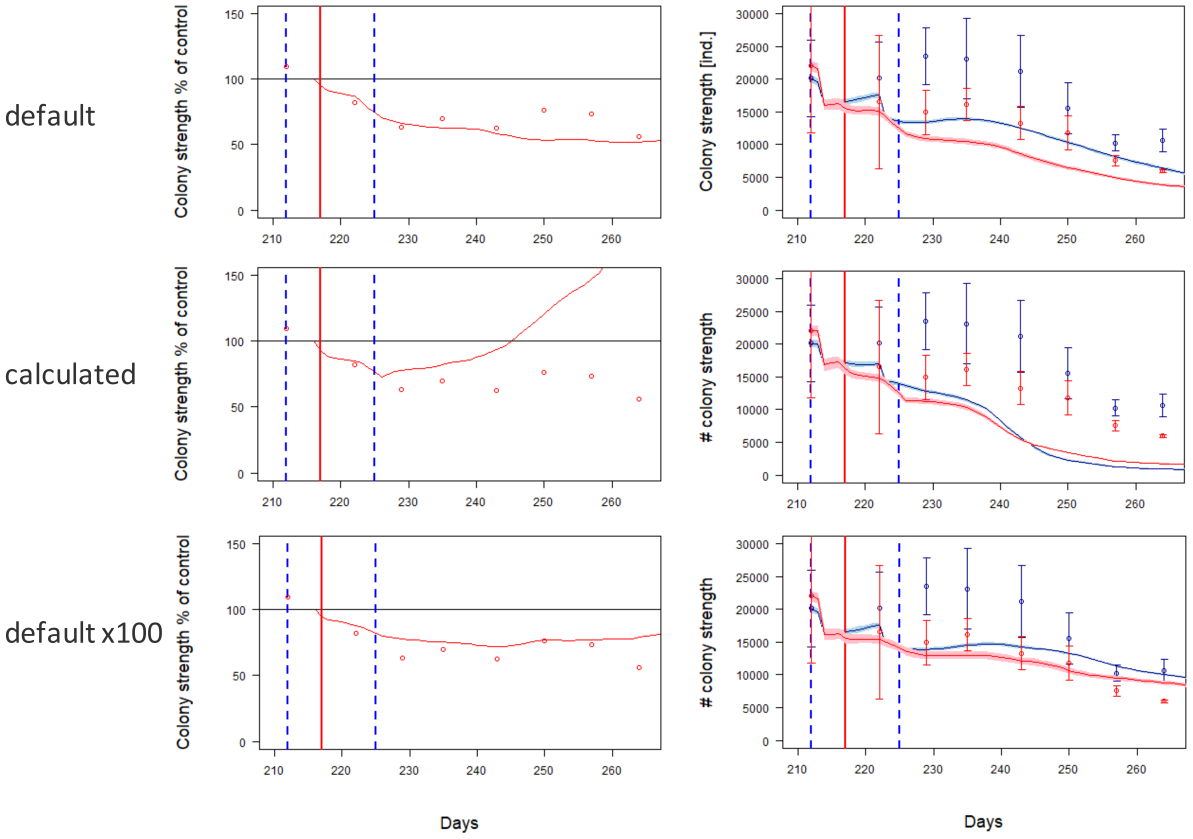

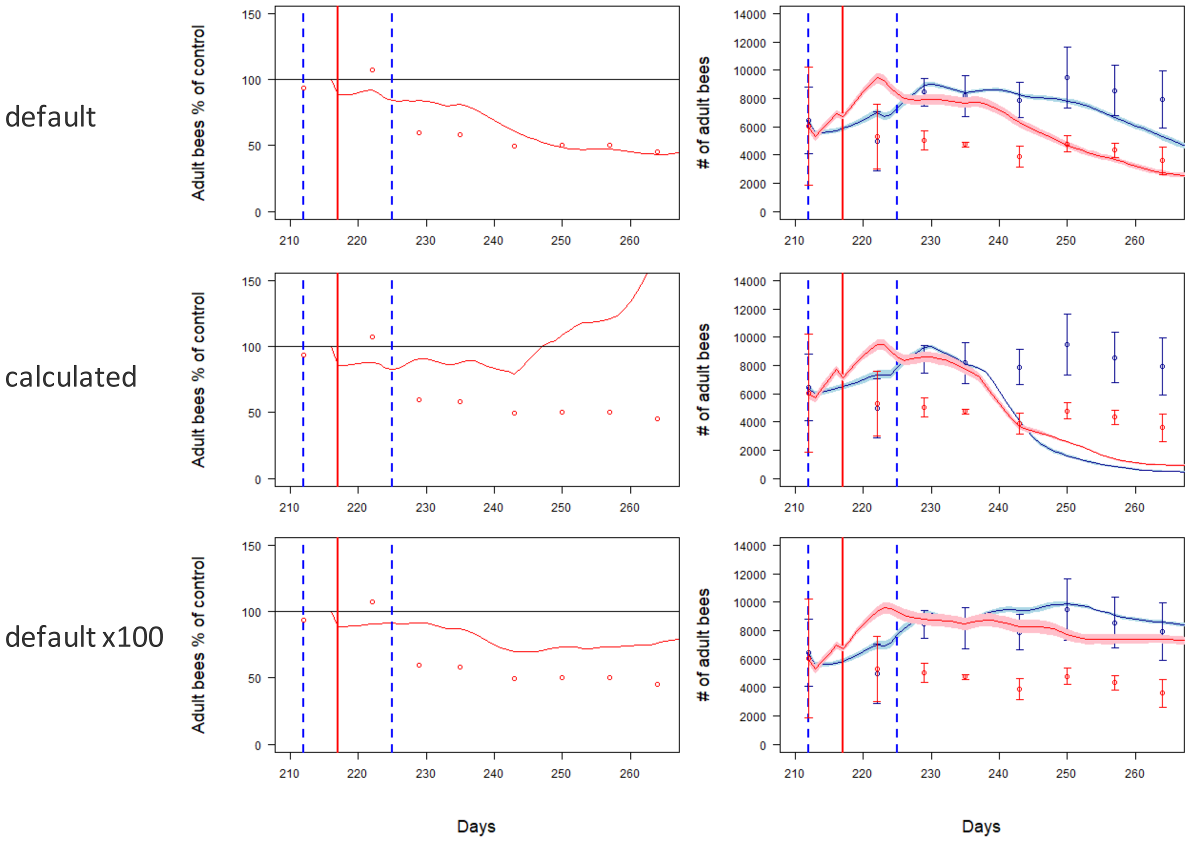

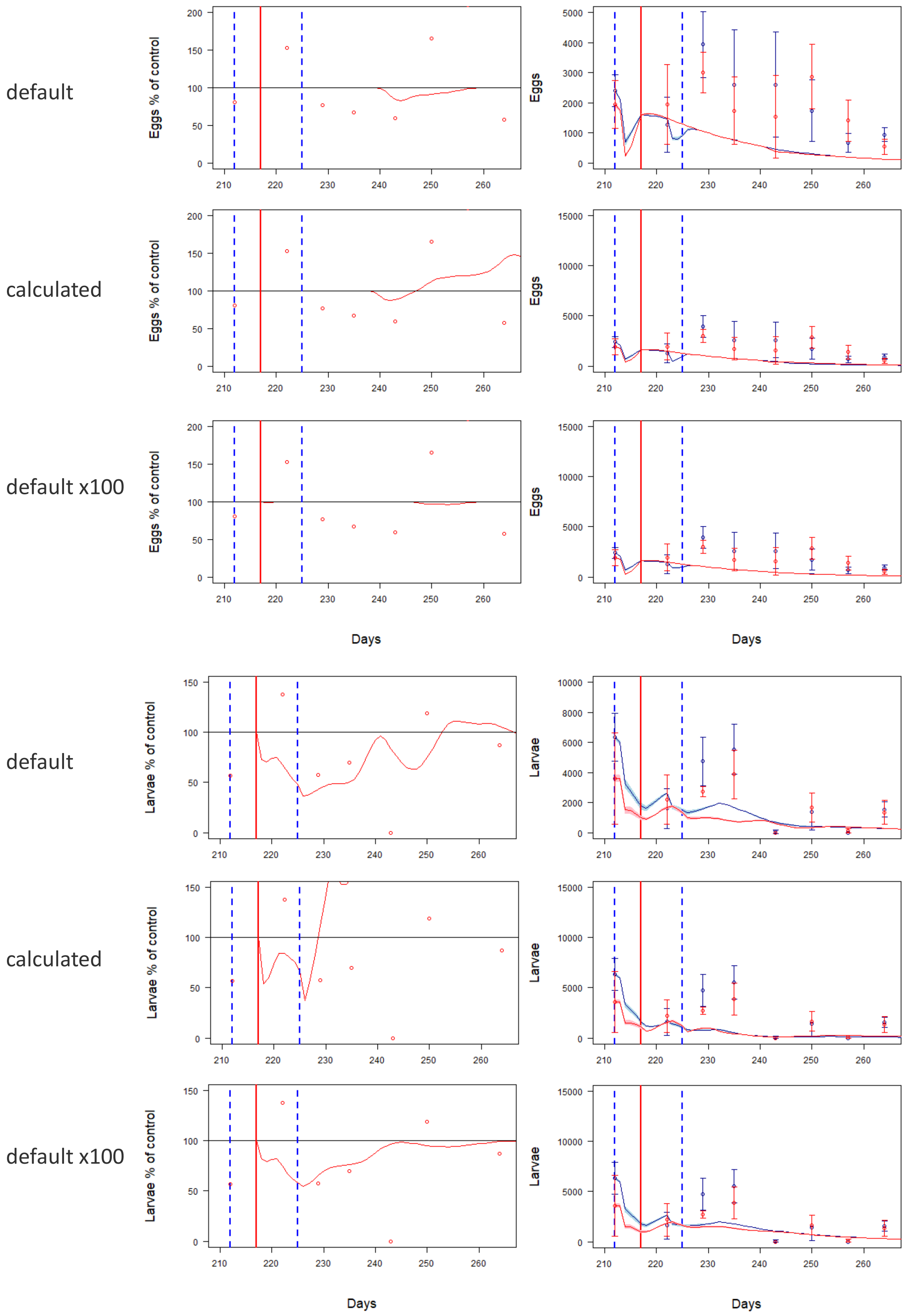

default
calculated
default x100
Eggs % of control
Eggs
Days
default
calculated
default x100
Larvae % of control
Larvae
Days

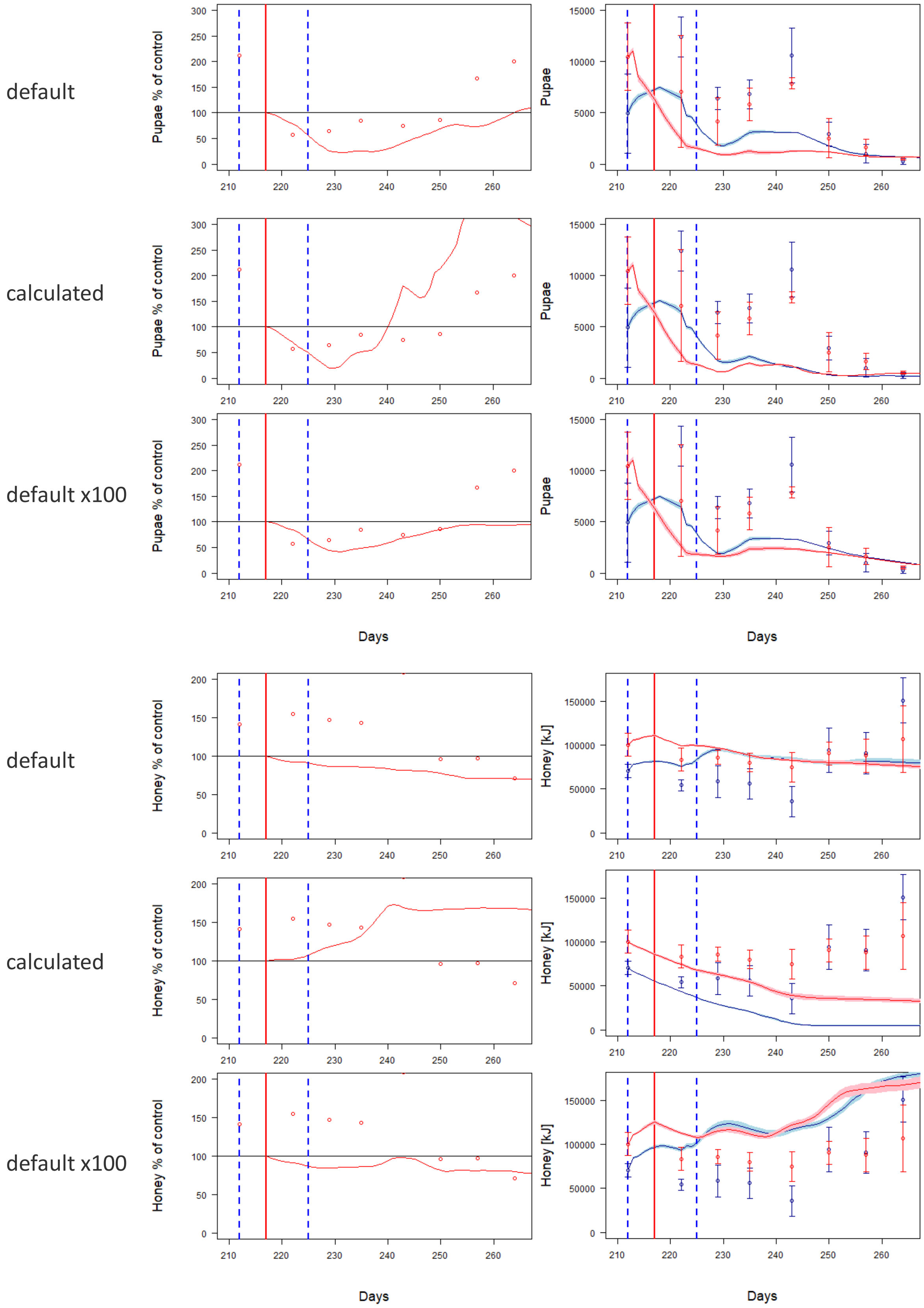
default
calculated
default x100
Pupae % of control
Pupae
Days
default
calculated
default x100
Honey % of control
Honey [kJ]
Days

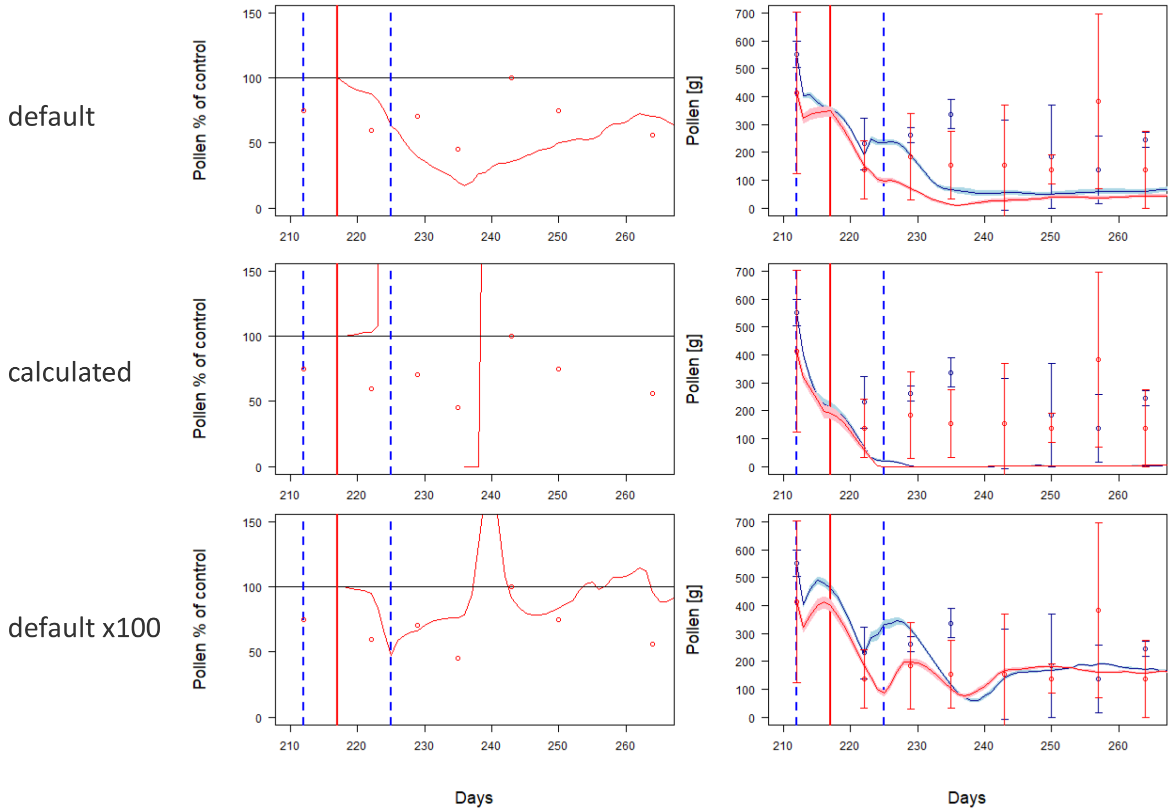
default
calculated
default x100
Pollen % of control
Pollen [g]
Days
Days

1.4 Dimethoate: In-hive/forager ratio

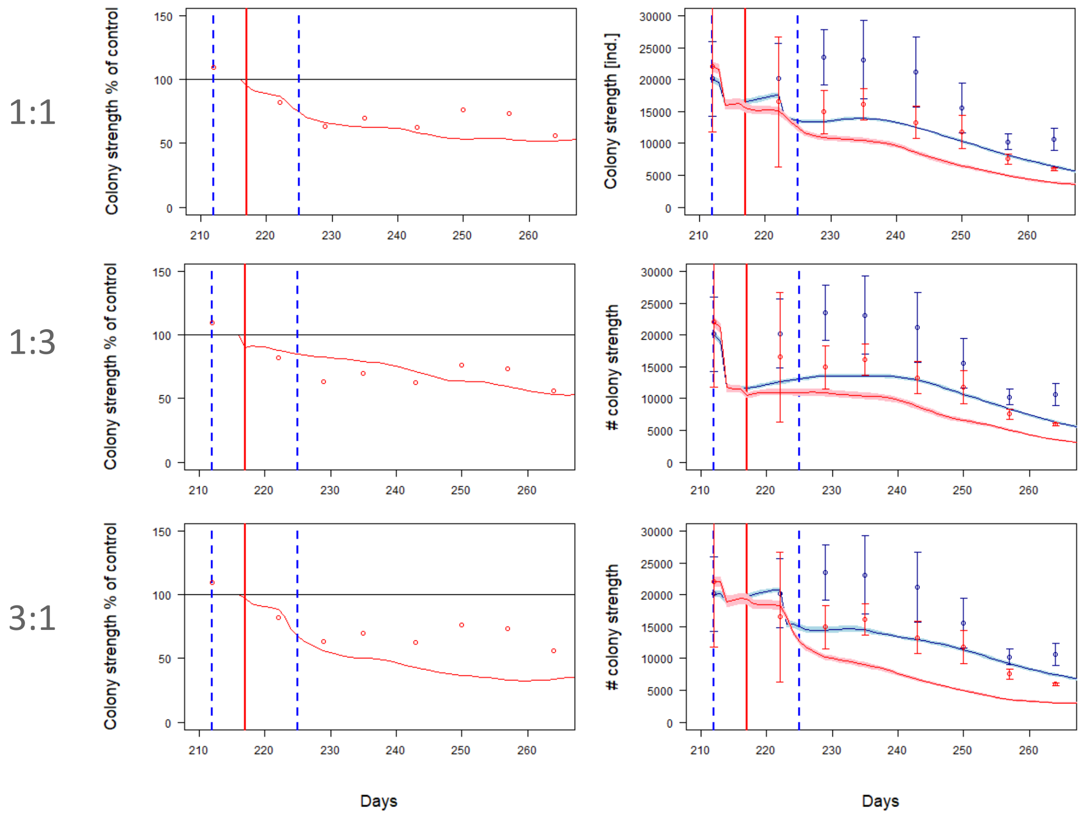

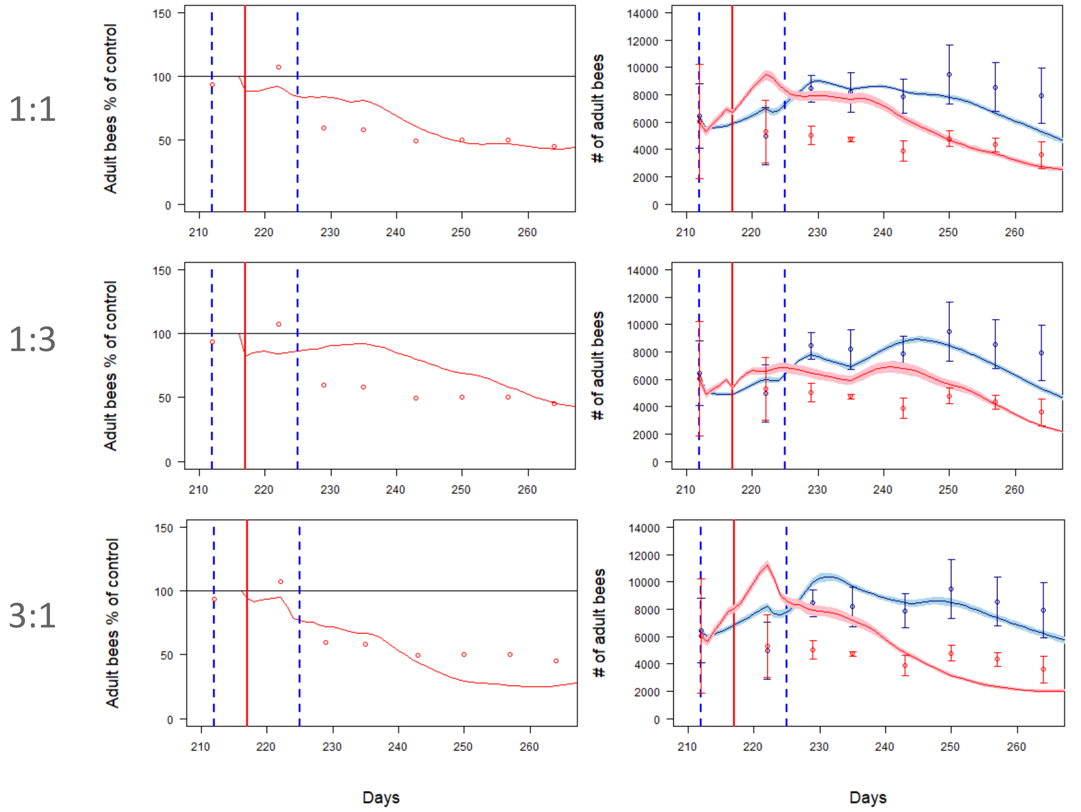

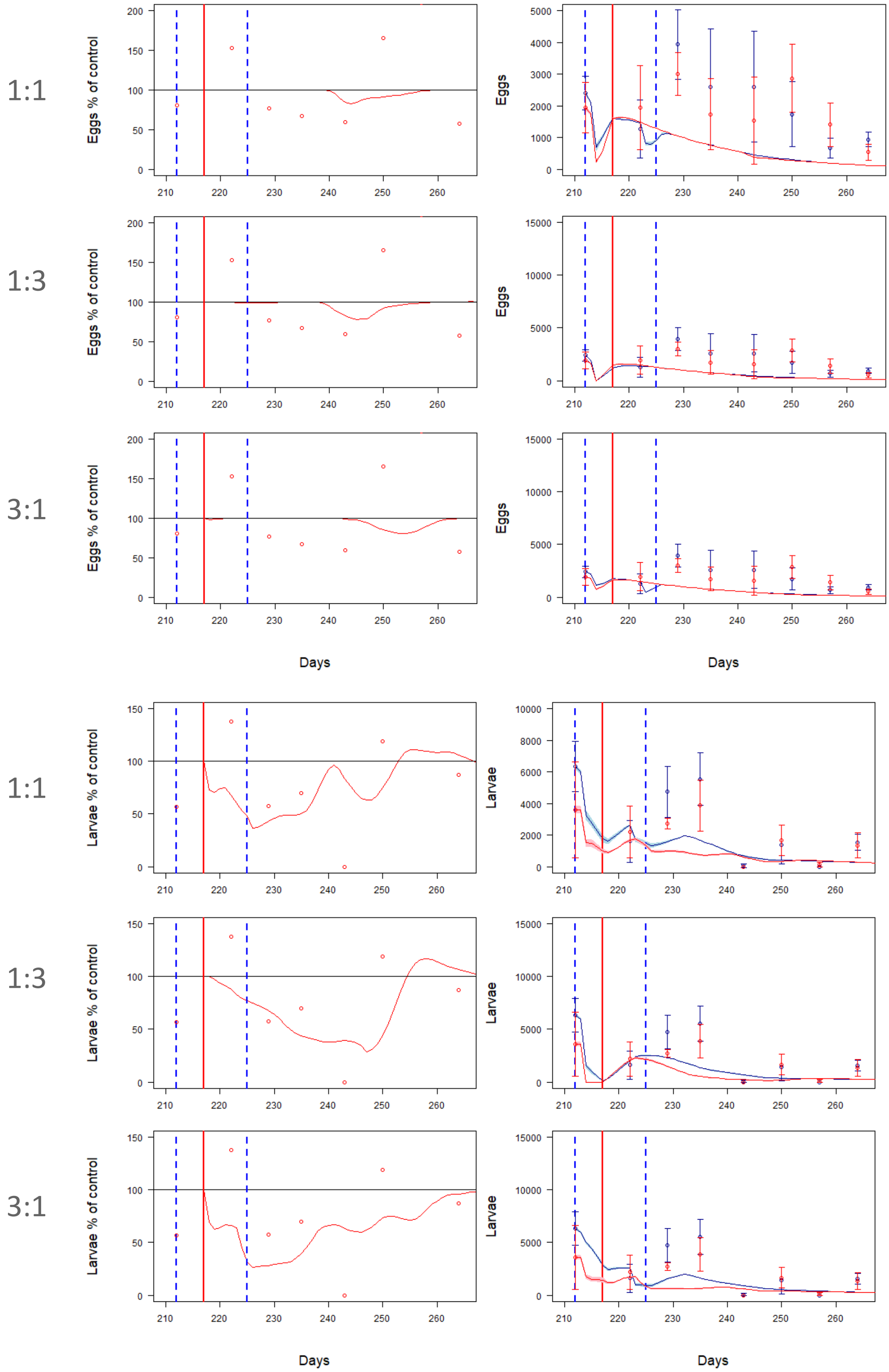
1:1
1:3
3:1
Eggs % of control
Eggs
Days
1:1
1:3
3:1
Larvae % of control
Larvae
Days

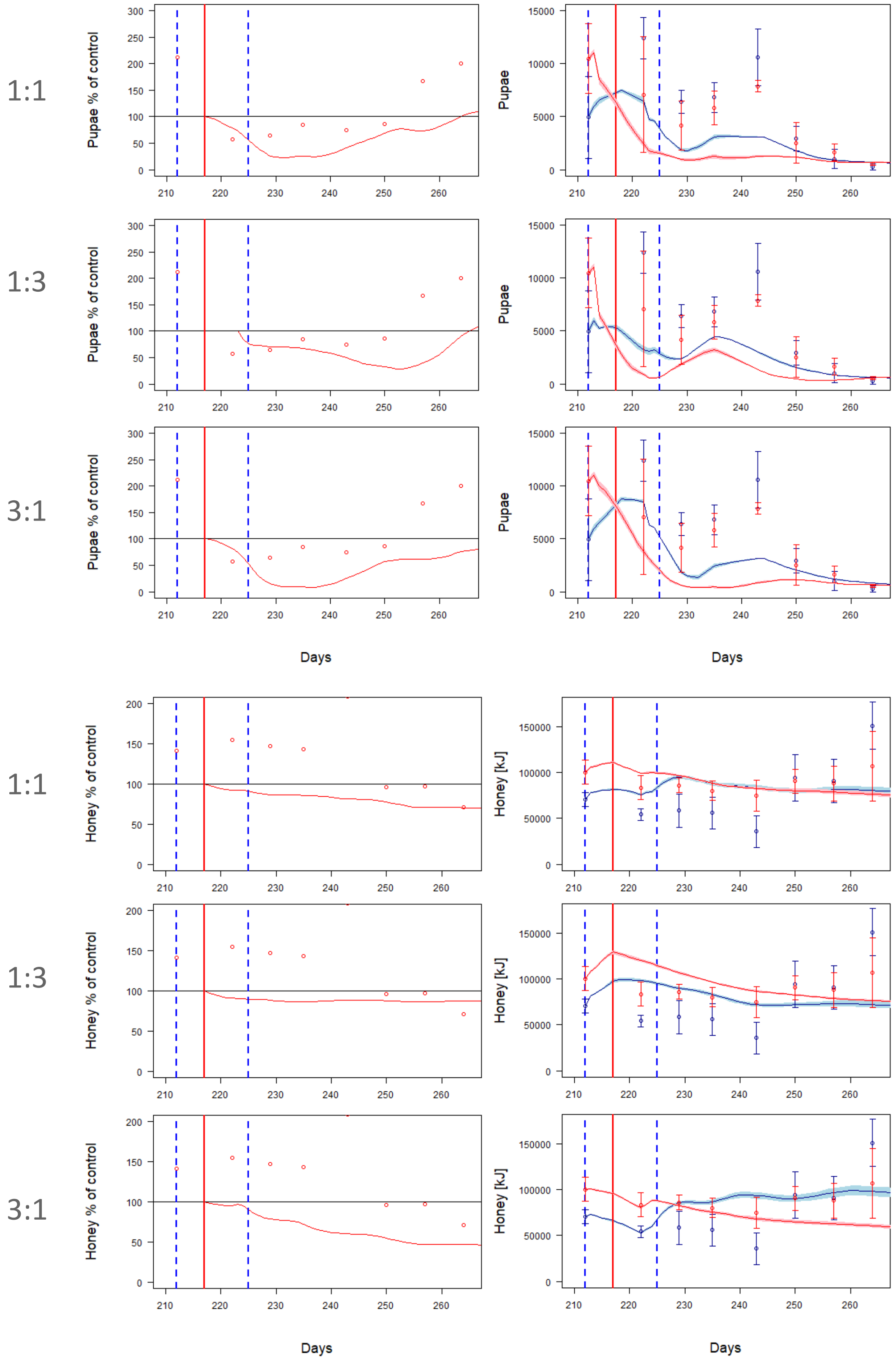
1:1
1:3
3:1
1:1
1:3
3:1
Pupae % of control
Pupae
Days
Days
Honey % of control
Honey [kJ]
Days
Days

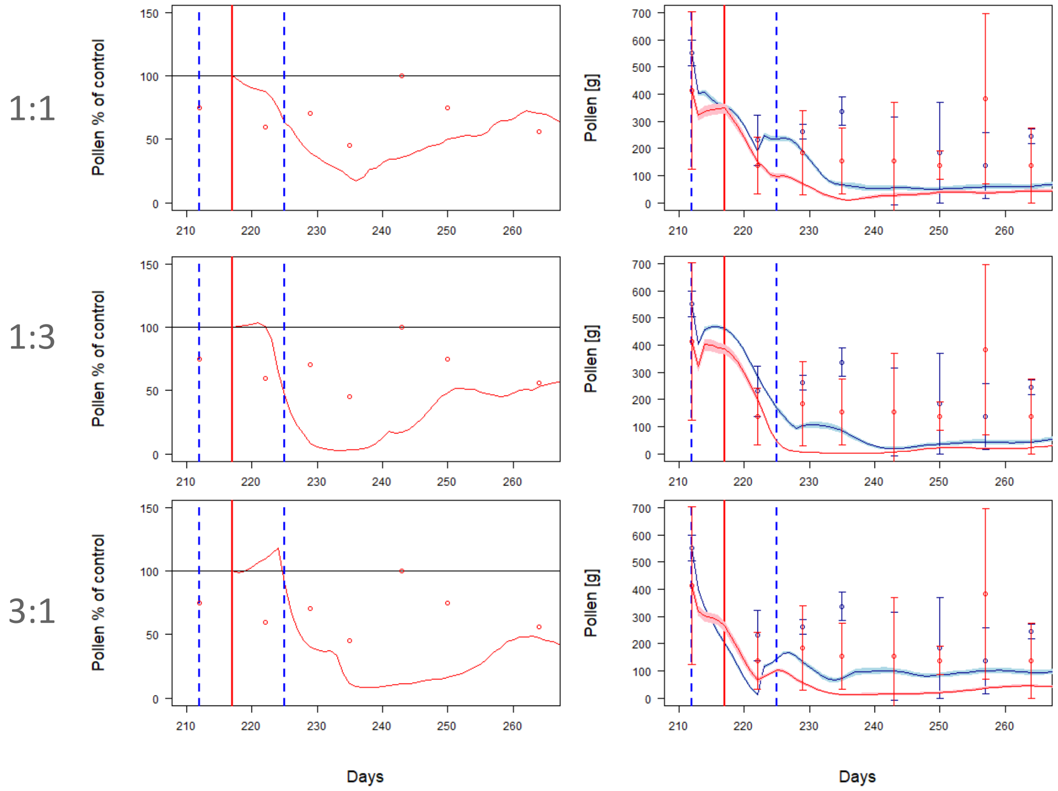

1:1
1:3
3:1
Pollen % of control
Pollen [g]
Days

2. Fenoxycarb

Table 2 Effects relative to control on the last day of observation for fenoxycarb (day 230). The empirical values are originated from the semi-field studies, the default values are the values of the BEEHAVE$_{ecotox}$ model in its default settings. Values are averages of 10 runs.

[%]	Colony strength	Adult bees	Eggs	Larvae	Pupae	Honey	Pollen
Empirical	56.68	55.88	71.43	85.48	42.68	76.98	106.94
Default	**49.61**	**52.84**	**79.22**	**59.68**	**25.08**	**92.53**	**127.44**
1. Different age structure							
**3 young bee groups**	66.72	64.33	58.01	102.57	63.52	150.95	710.95
2. Distance to monitoring site							
**10 m**	51.75	56.77	76.77	61.72	22.05	112.22	154.7
**1000 m**	46.43	52.71	82.22	44.49	18.44	83.71	153.29
3. Nectar and pollen availability							
**Calculated**	59.71	59.37	49.42	175.98	981.71	Inf.	3490
**Default * 100**	46.11	46.88	94.65	59.67	31.72	80.03	66.5
4. In-hive/forager ratio							
**1:3**	45.96	46.59	72.38	79.62	9.23	129.2	1055.76
**3:1**	78.08	72.68	95.51	100.42	82.69	87.29	104.45

2.1 Fenoxycarb: Different age structure

default

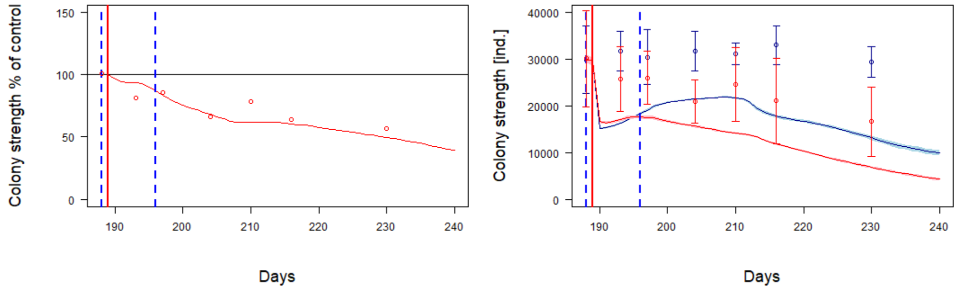

with 3 young bee groups at initialization

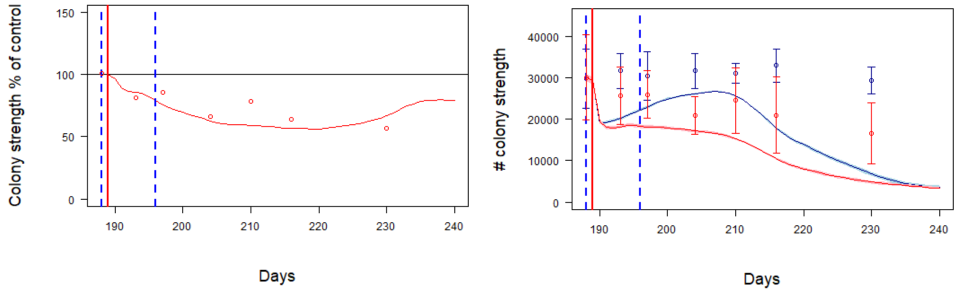

default

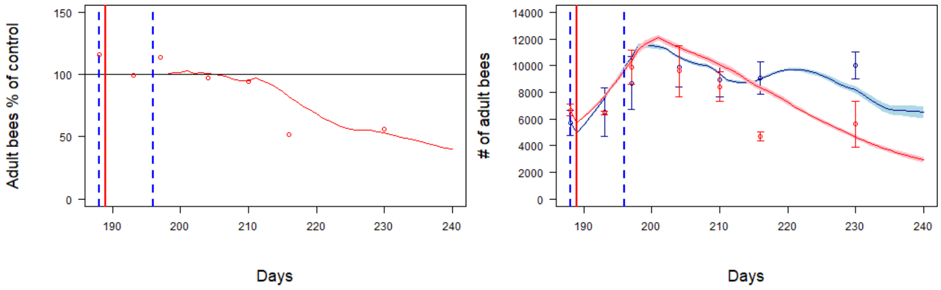

with 3 young bee groups at initialization

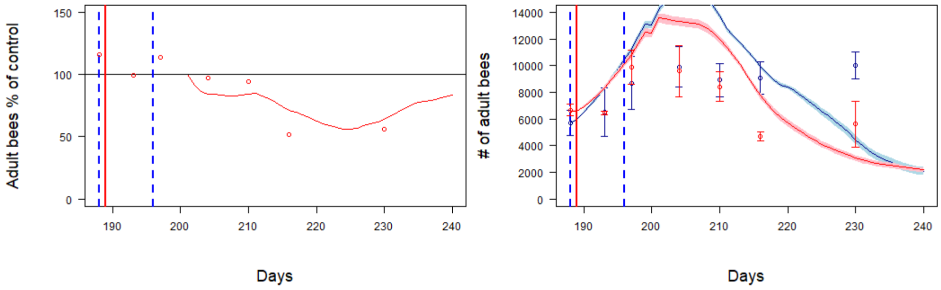

default

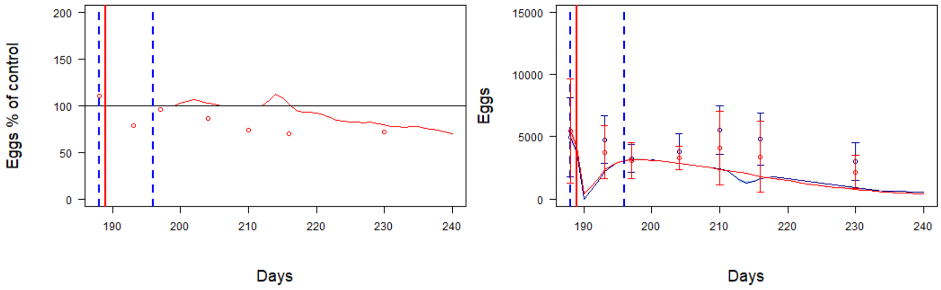

with 3 young bee groups at initialization

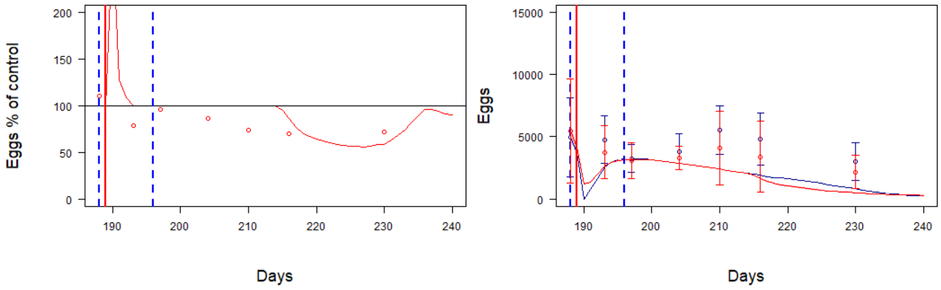

default

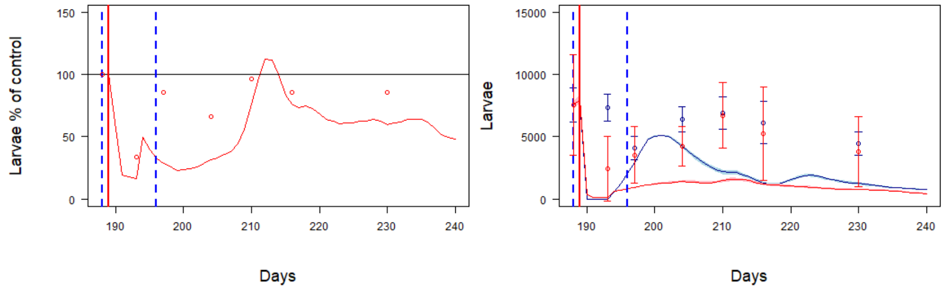

with 3 young bee groups at initialization

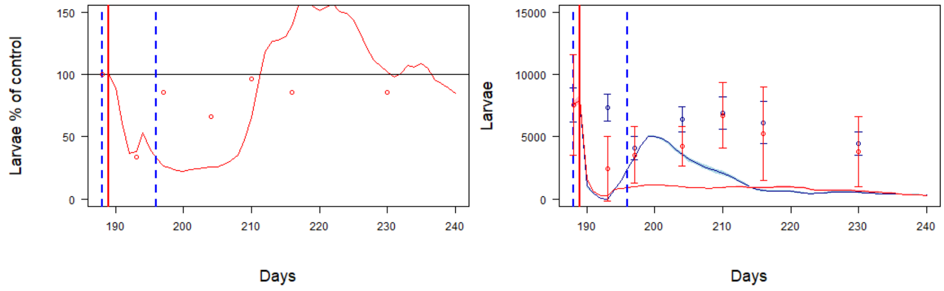

default

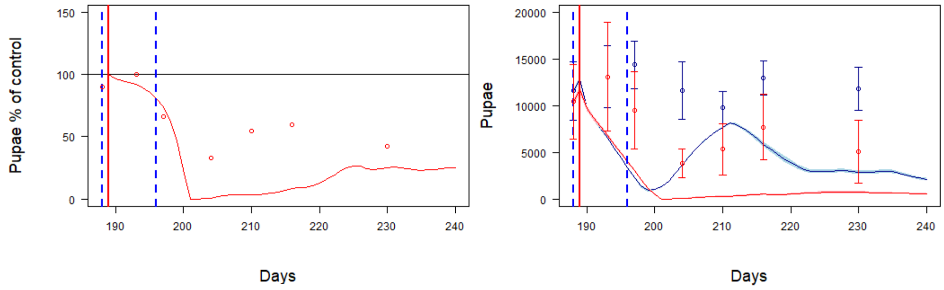

with 3 young bee groups at initialization

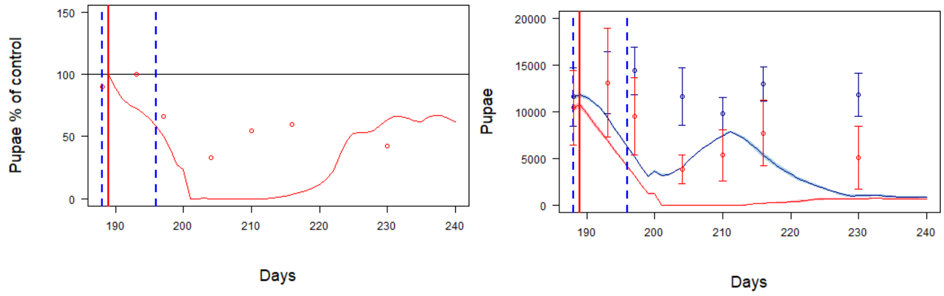

default

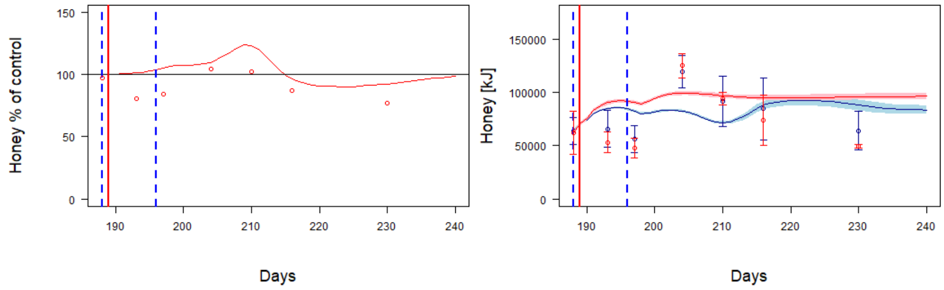

with 3 young bee groups at initialization

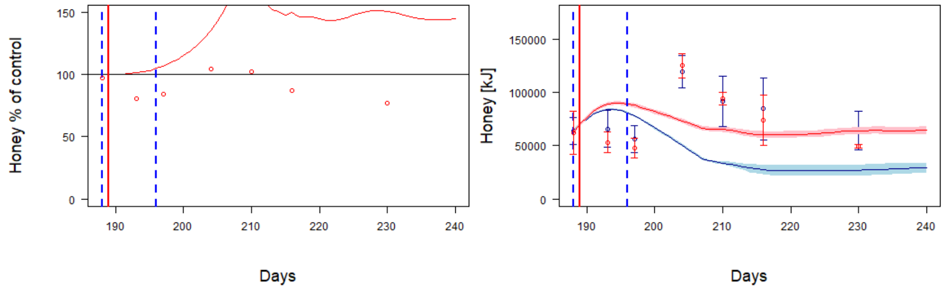

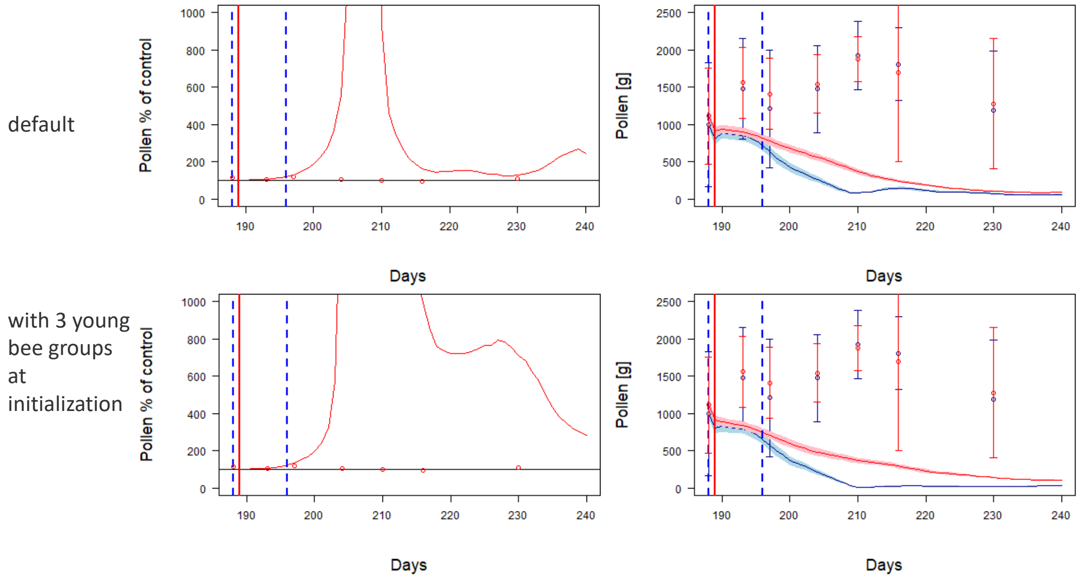
default
with 3 young bee groups at initialization
Pollen % of control
Pollen [g]
Days

2.2 Fenoxycarb: Distance to monitoring site

100m

10m

1000m

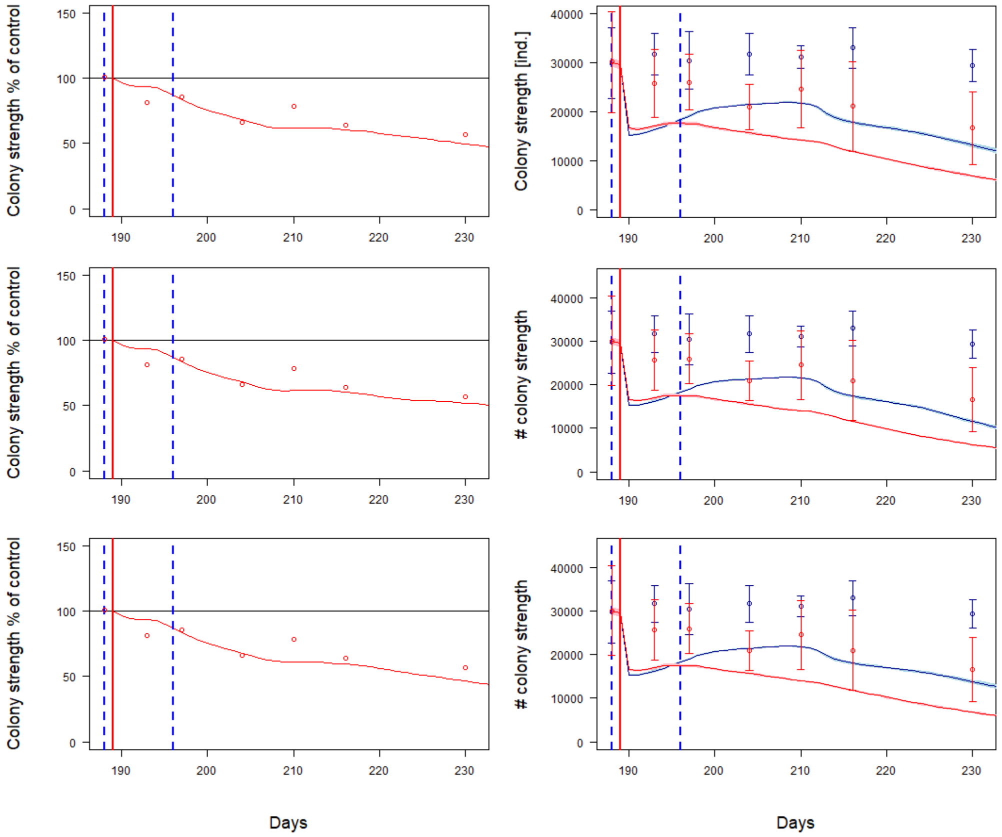

100m

10m

1000m

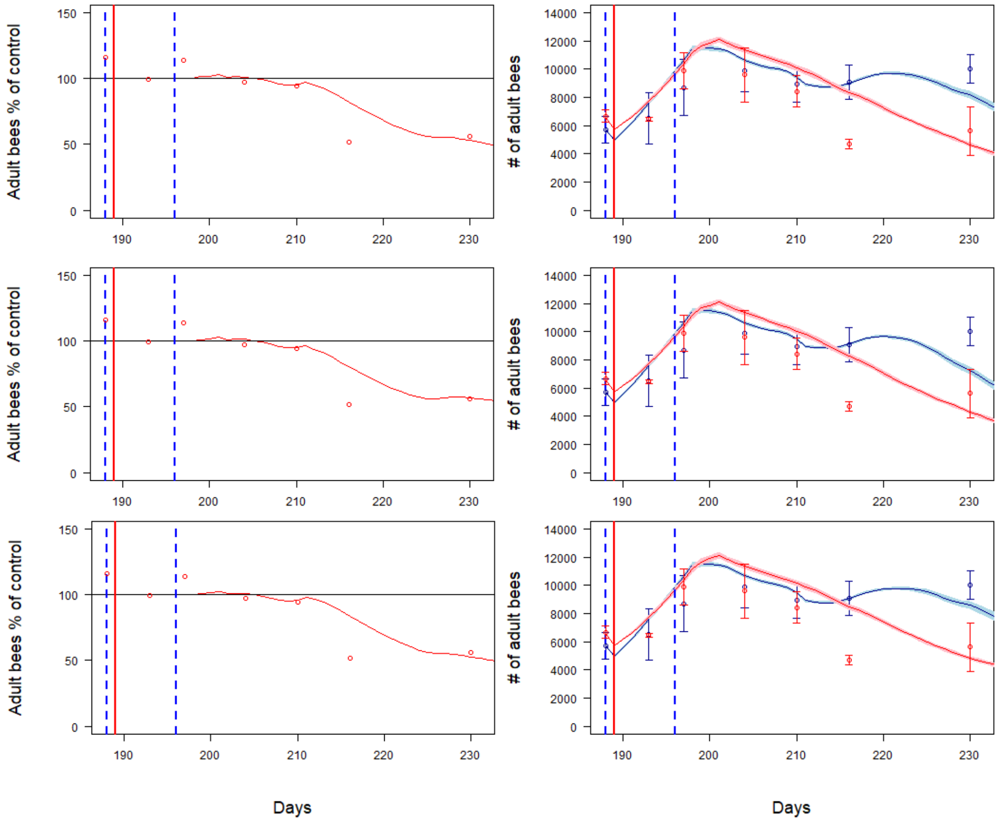

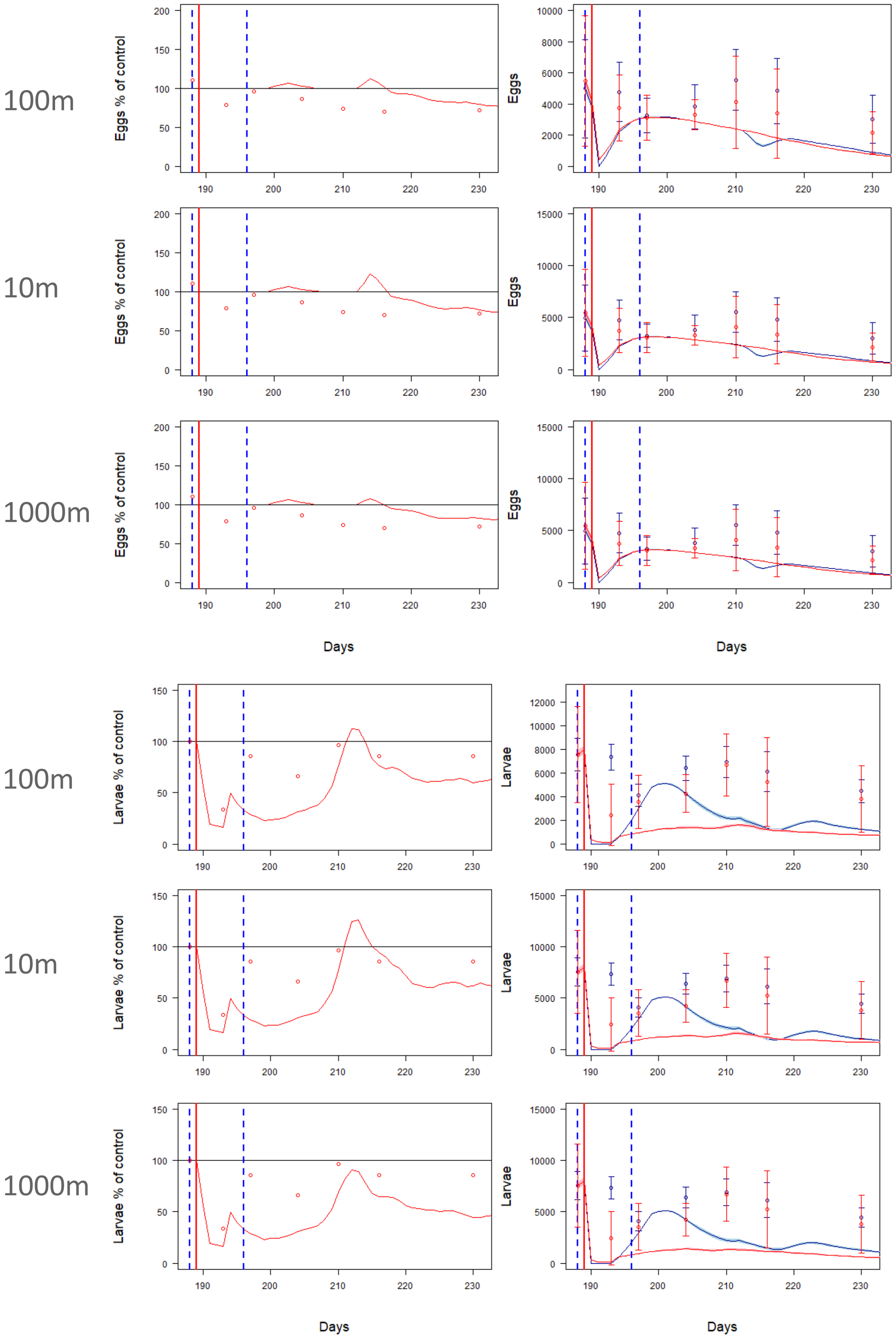
100m
10m
1000m
Eggs % of control
Eggs
Days
100m
10m
1000m
Larvae % of control
Larvae
Days

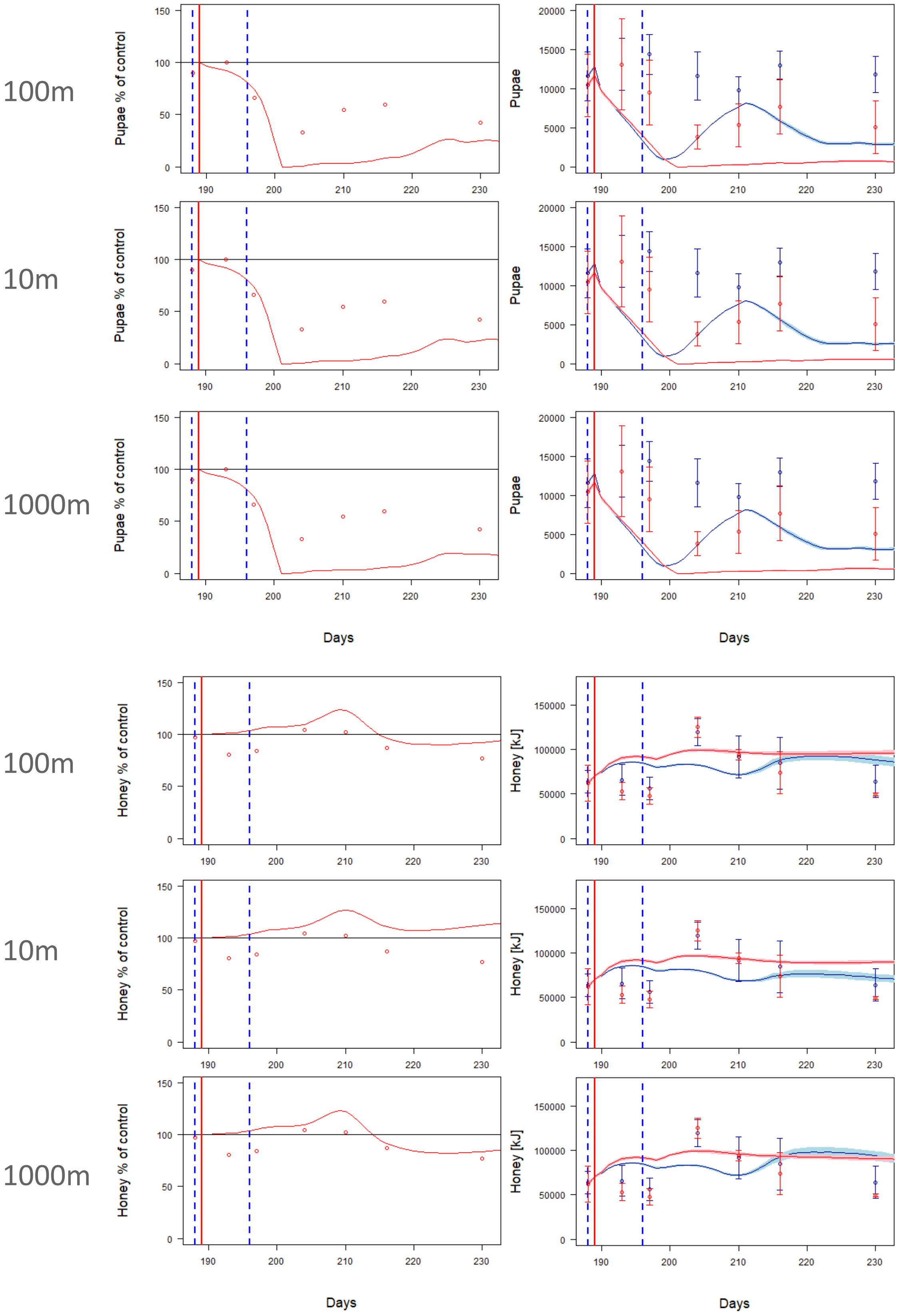

100m
10m
1000m
100m
10m
1000m
Pupae % of control
Pupae
Honey % of control
Honey [kJ]
Days

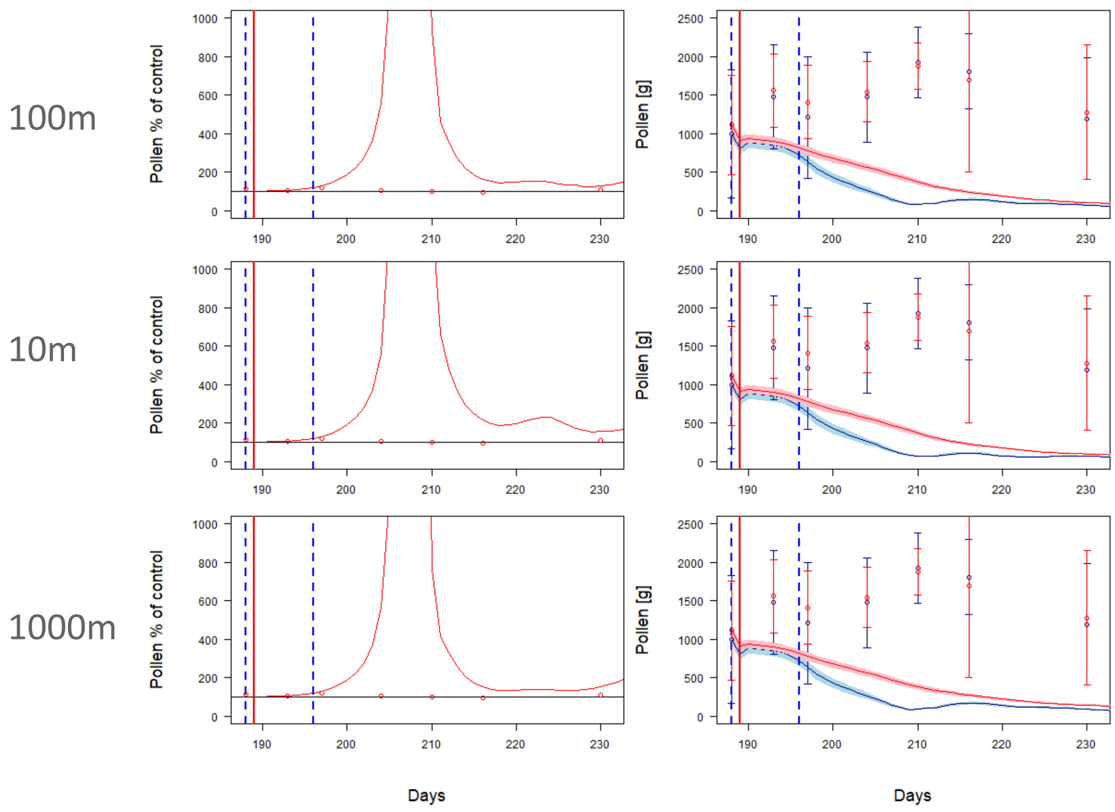

100m
10m
1000m
Pollen % of control
Pollen [g]
Days

2.3 Fenoxycarb: Nectar and pollen availability

default

calculated

default x100

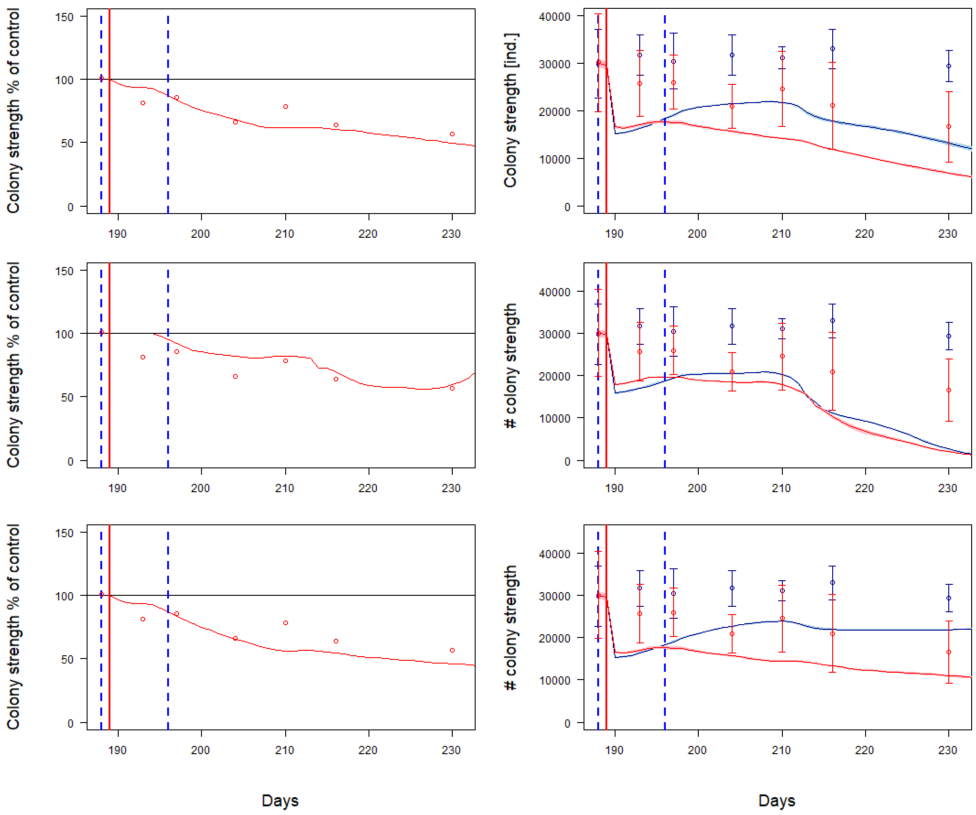

default

calculated

default x100

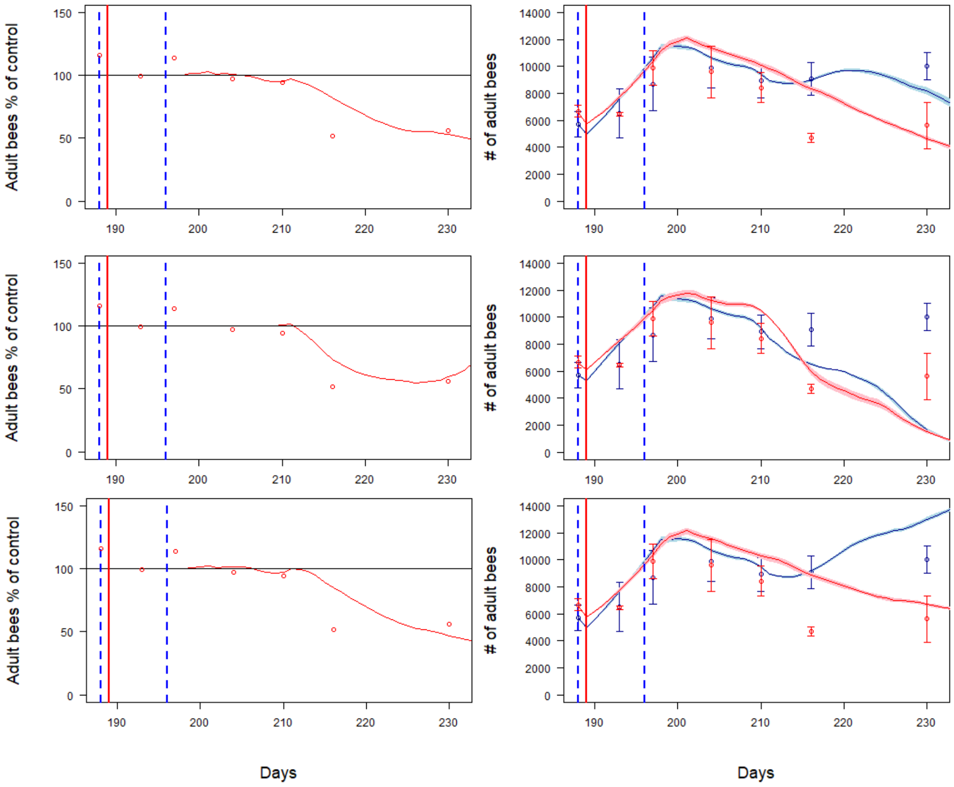

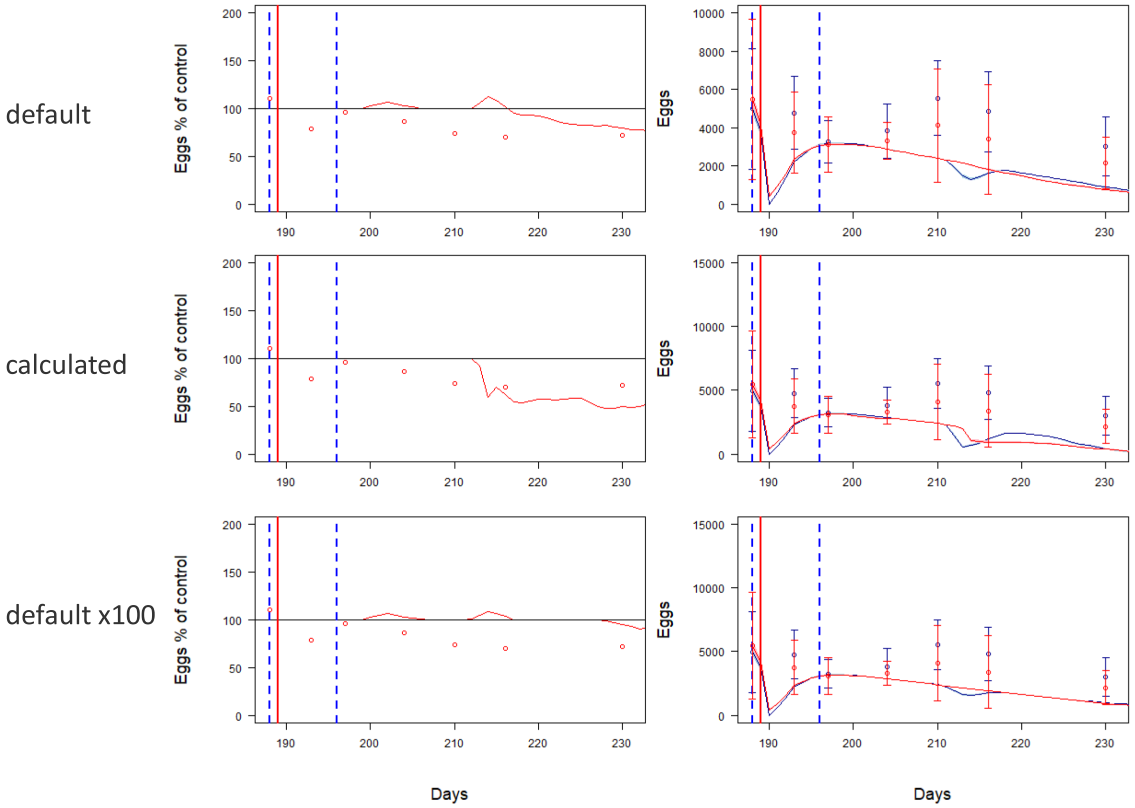
default
calculated
default x100
Eggs % of control
Eggs
Days
Days

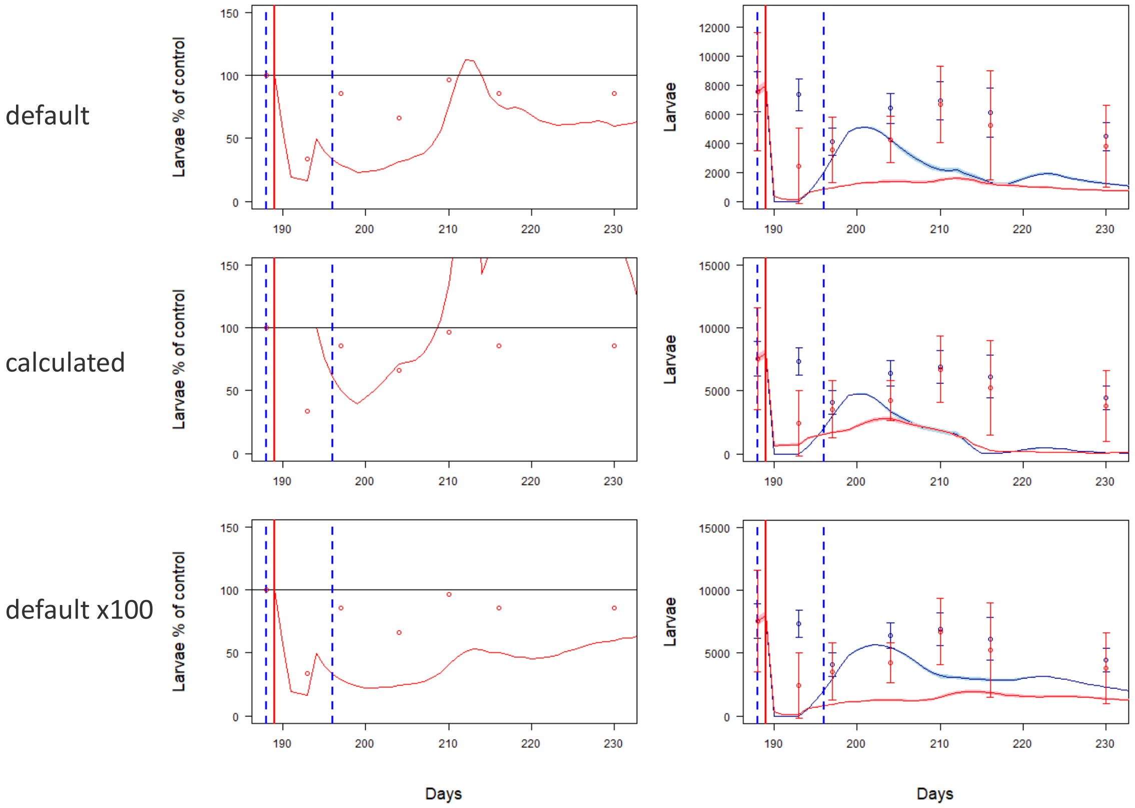
default
calculated
default x100
Larvae % of control
Larvae
Days
Days

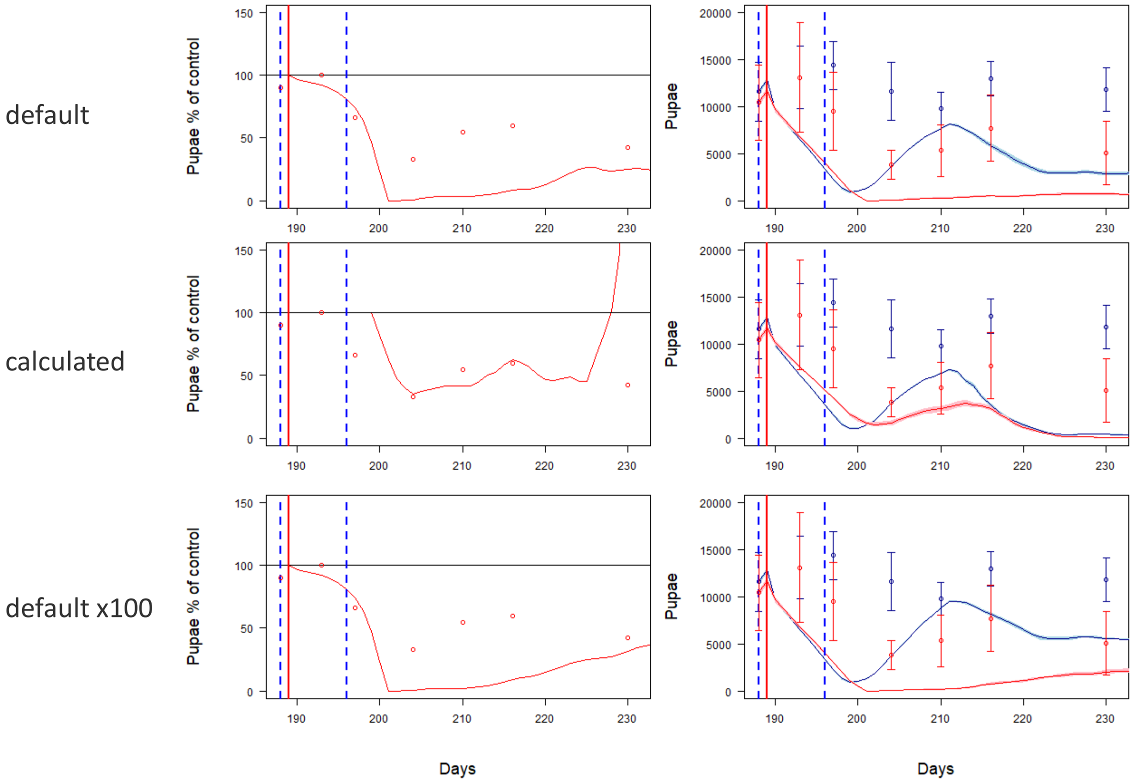
default
calculated
default x100
Pupae % of control
Pupae
Days
Days

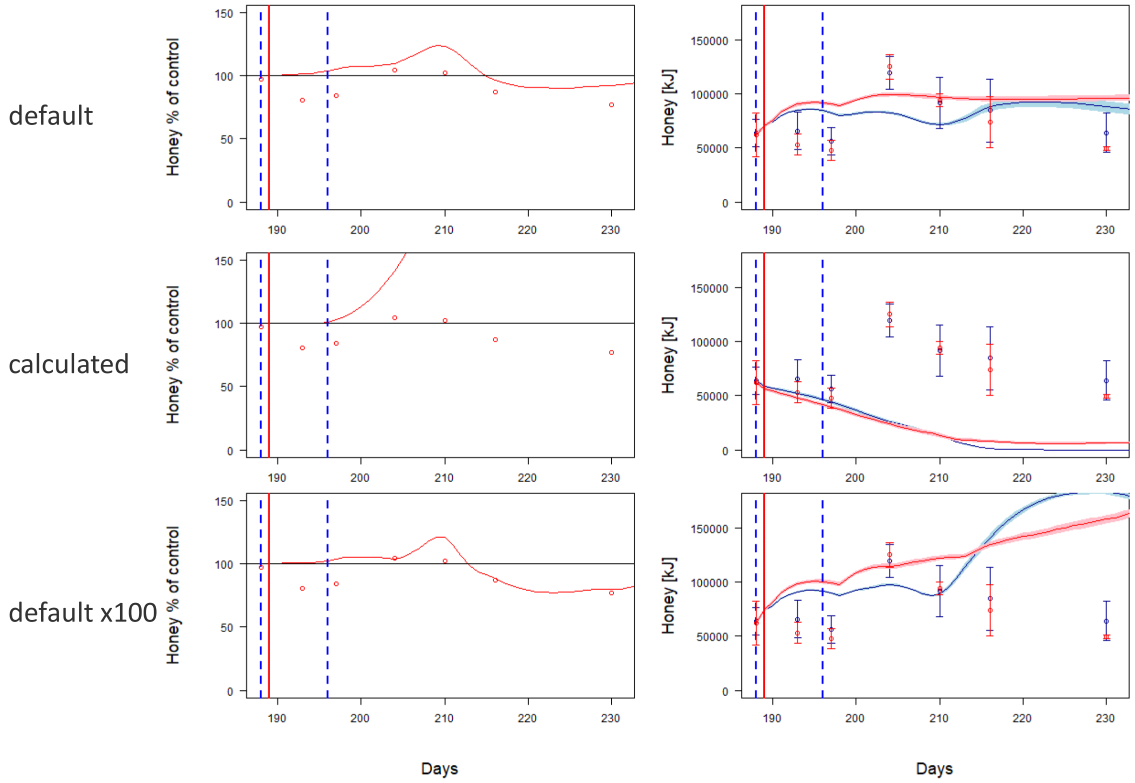
default
calculated
default x100
Honey % of control
Honey [kJ]
Days
Days

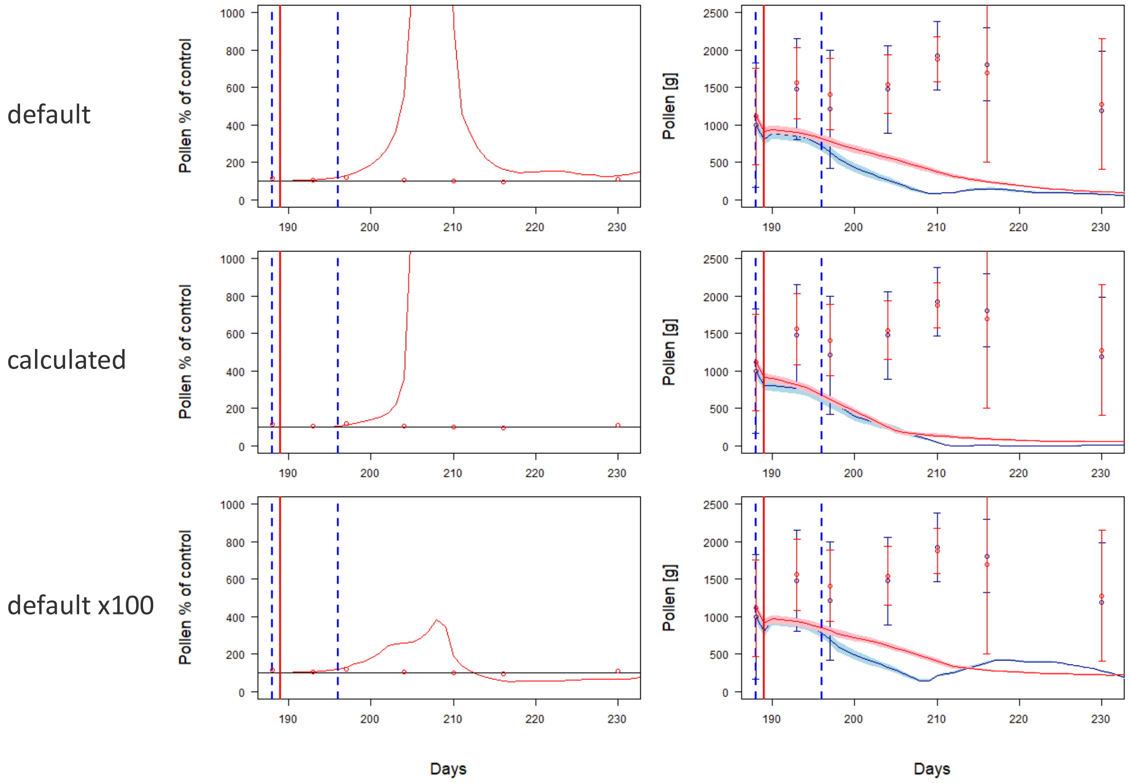
default
calculated
default x100
Pollen % of control
Pollen [g]
Days
Days

2.4 Fenoxycarb: In-hive/forager ratio

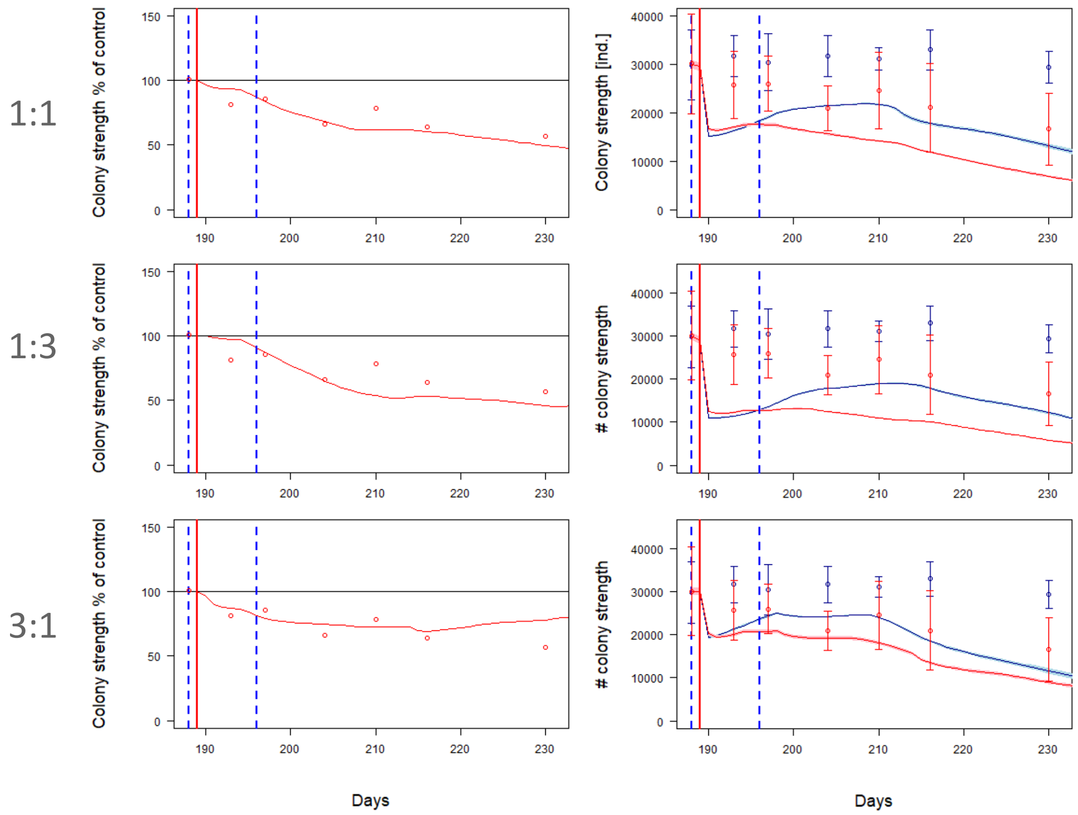

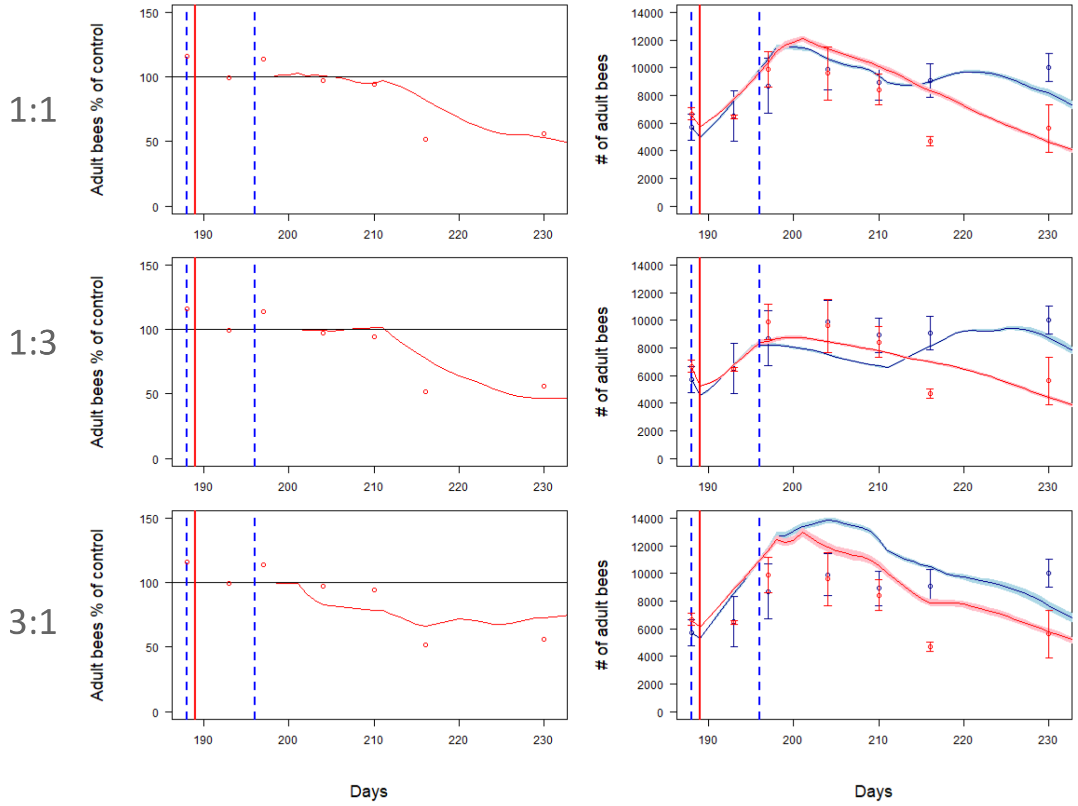

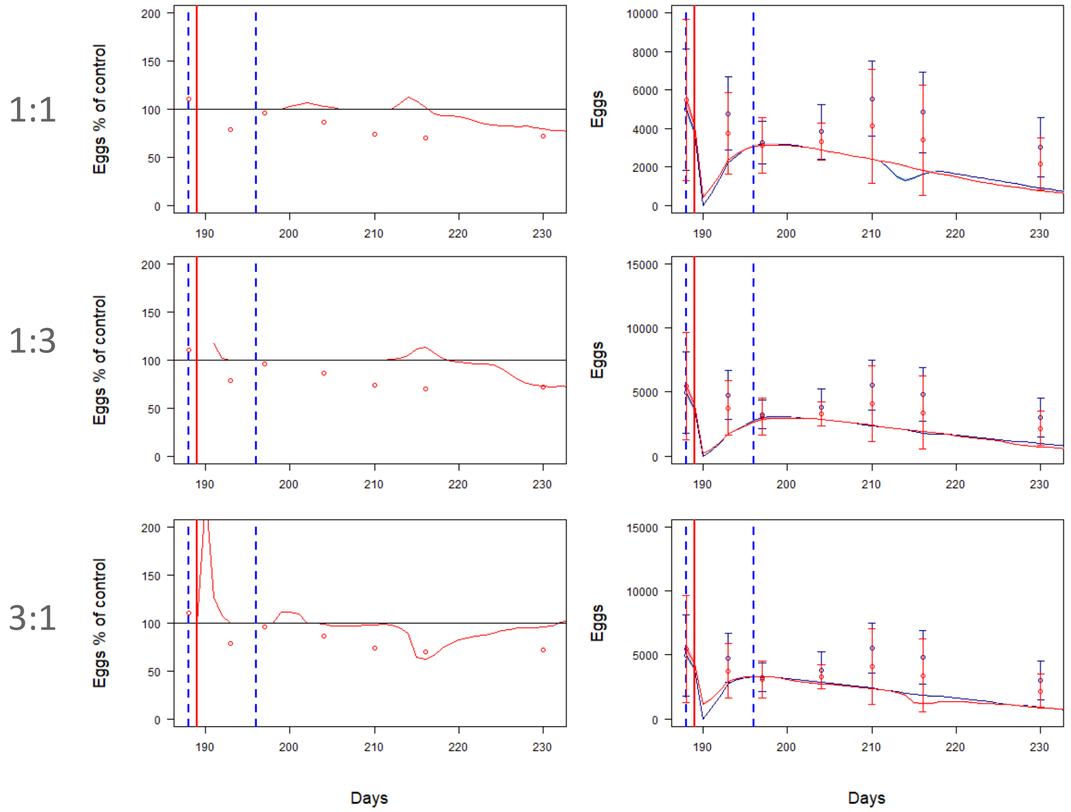
1:1
1:3
3:1
Eggs % of control
Eggs
Days

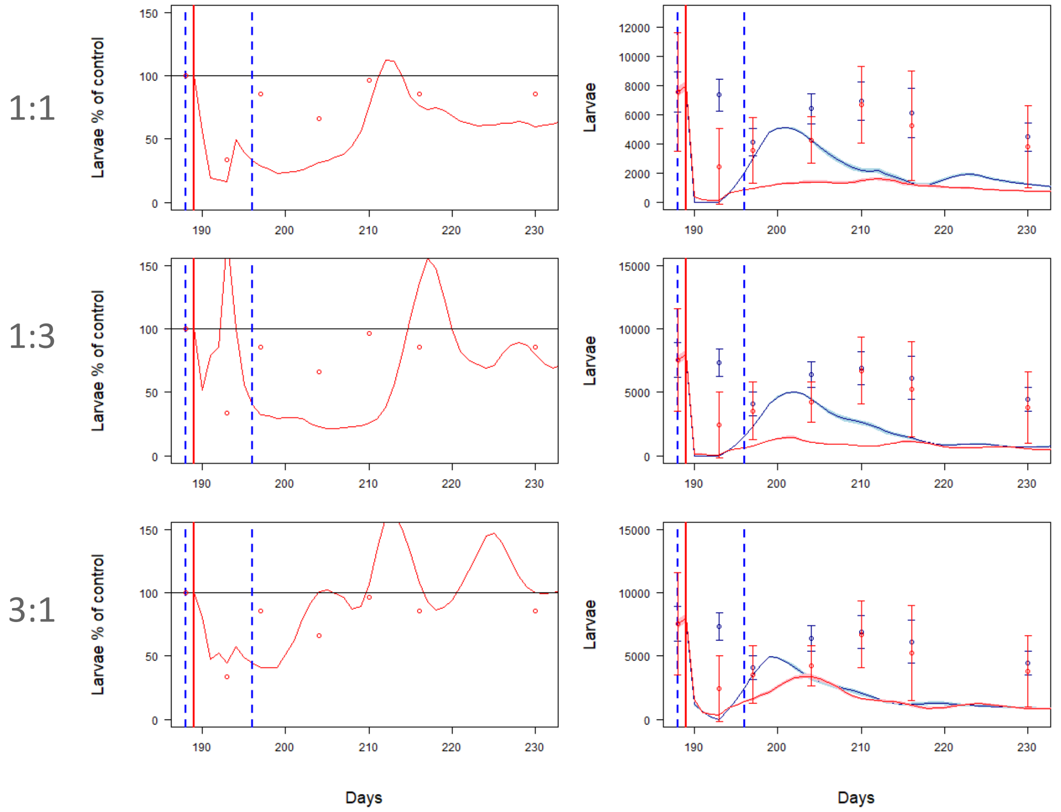
1:1
1:3
3:1
Larvae % of control
Larvae
Days

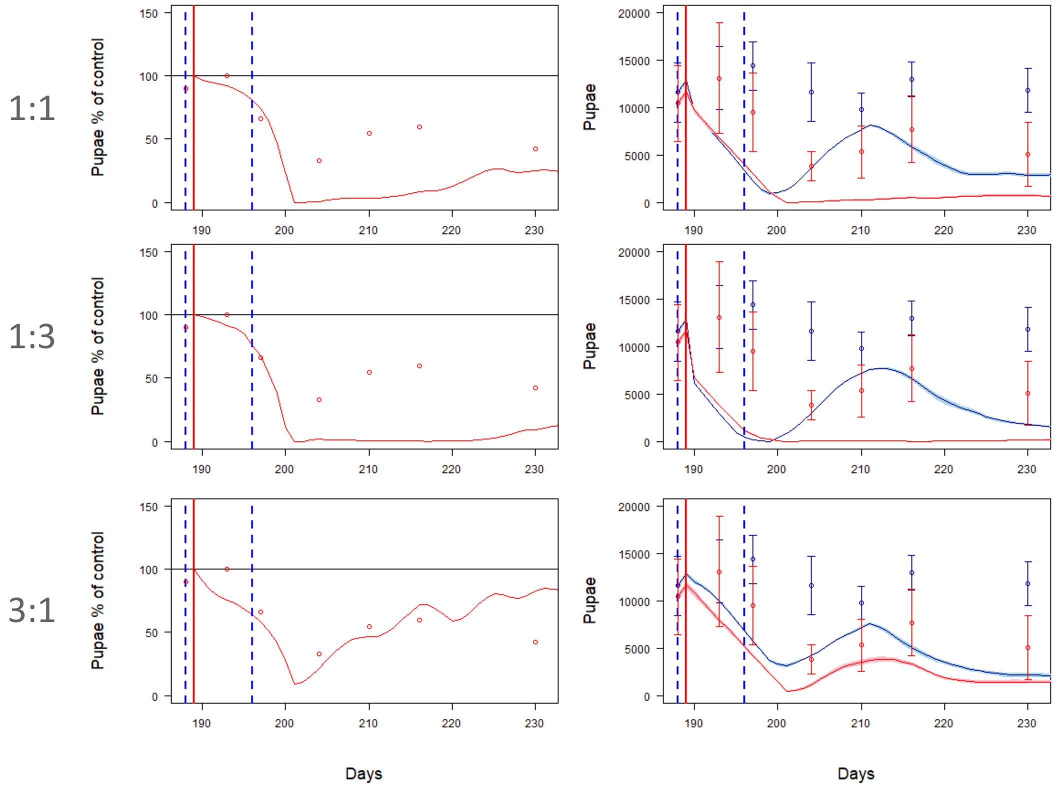
1:1
1:3
3:1
Pupae % of control
Pupae
Days
Days

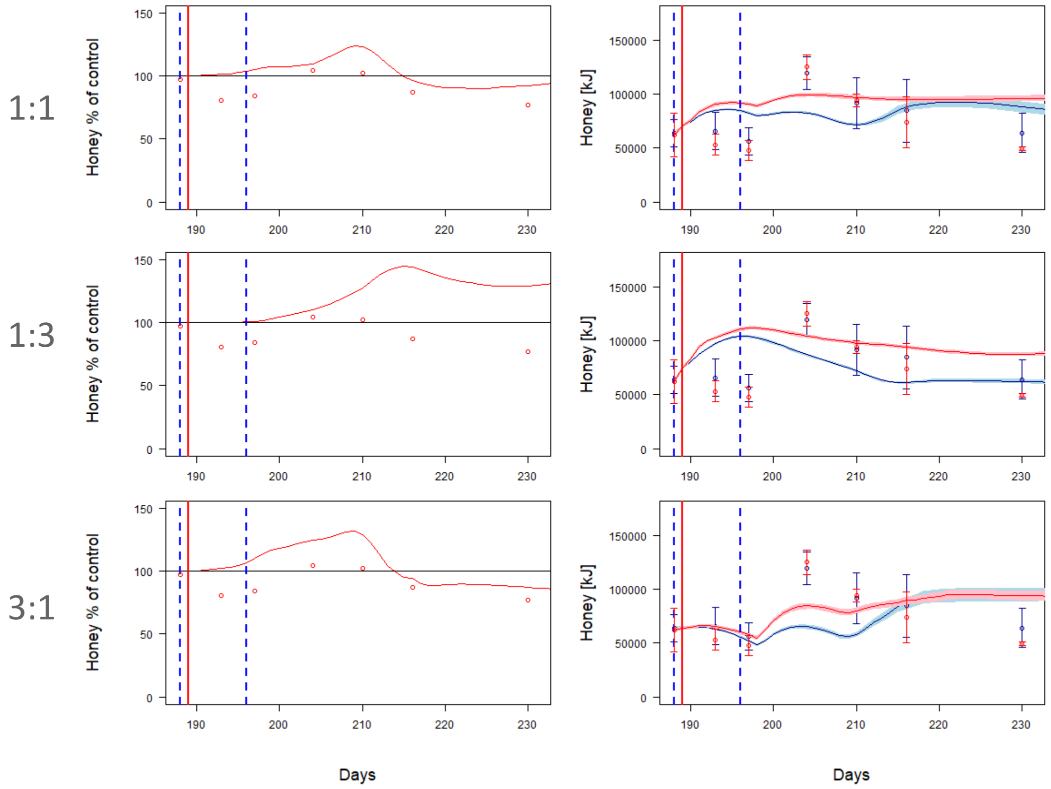
1:1
1:3
3:1
Honey % of control
Honey [kJ]
Days
Days

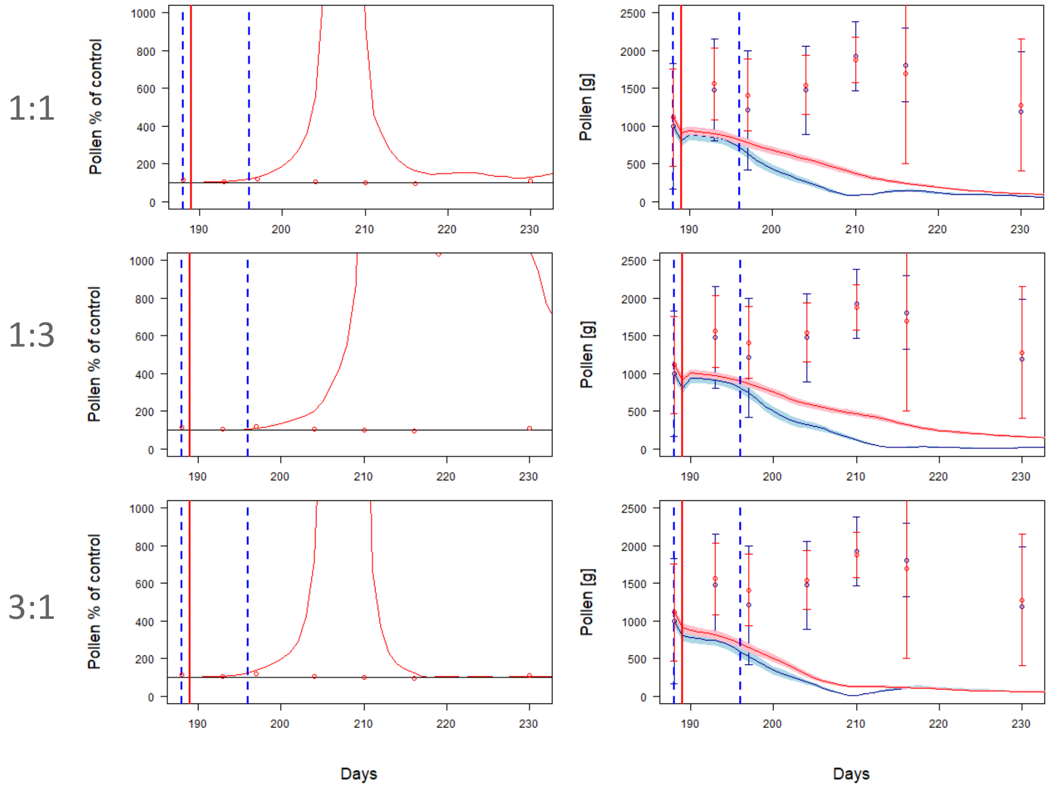

1:1
1:3
3:1
Pollen % of control
Pollen [g]
Days
